# iSCORE-PD: an isogenic stem cell collection to research Parkinson’s disease

**DOI:** 10.1038/s41467-026-74355-8

**Published:** 2026-06-17

**Authors:** Oriol Busquets, Hanqin Li, Khaja Mohieddin Syed, Pilar Alvarez Jerez, Jesse Dunnack, Riana Lo Bu, Yogendra Verma, Gabriella R. Pangilinan, Annika Martin, Jannes Straub, YuXin Du, Vivien M. Simon, Steven Poser, Zipporiah Bush, Jessica Diaz, Atehsa Sahagun, Jianpu Gao, Samantha Hong, Dena G. Hernandez, Kristin S. Levine, Nathalie Pochet, Ezgi O. Booth, Marco Blanchette, Helen S. Bateup, Donald C. Rio, Cornelis Blauwendraat, Dirk Hockemeyer, Frank Soldner

**Affiliations:** 1 Dominick P. Purpura Department of Neuroscience, Rose F. Kennedy Center, Albert Einstein College of Medicine, Bronx, NY USA; 2 Ruth L. and David S. Gottesman Institute for Stem Cell and Regenerative Medicine Research, Albert Einstein College of Medicine, Bronx, NY USA; 3 Aligning Science Across Parkinson’s (ASAP) Collaborative Research Network, Chevy Chase, MD USA; 4 Department of Molecular and Cell Biology, University of California, Berkeley, Berkeley, CA USA; 5 Innovative Genomics Institute, University of California, Berkeley, CA USA; 6 Center for Alzheimer’s and Related Dementias, National Institute on Aging and National Institute of Neurological Disorders and Stroke, National Institutes of Health, Bethesda, MD USA; 7 Department of Neurodegenerative Disease, UCL Queen Square Institute of Neurology, University College London, London, UK; 8 Department of Genetics, Albert Einstein College of Medicine, Bronx, NY USA; 9 Department of Neuroscience, University of California, Berkeley, Berkeley, CA USA; 10 Laboratory of Neurogenetics, National Institute on Aging, National Institutes of Health, Bethesda, MD USA; 11 LiftOff Biosolution, Santa Cruz, CA USA; 12 Chan Zuckerberg Biohub, San Francisco, CA USA

**Keywords:** Parkinson's disease, Embryonic stem cells, Stem-cell biotechnology

## Abstract

Genome-edited human pluripotent stem cells (hPSCs) provide a powerful platform to study complex diseases such as Parkinson’s disease (PD). Here, we describe iSCORE-PD, an isogenic collection of 65 genome-edited hPSC lines carrying disease-causing or high-risk variants in 11 PD-linked genes (*SNCA, PRKN, PINK1, DJ1/PARK7, LRRK2, ATP13A2, FBXO7, DNAJC6, SYNJ1, VPS13C*, and *GBA1*). All lines are derived from a well-characterized female hESC line and subjected to extensive quality control. Whole-genome sequencing reveals that genetic variation between lines, largely confined to non-coding regions, is minimal relative to inter-individual differences in patient-derived hiPSCs, with most variation arising from random mutations acquired during cell culture rather than genome-editing-induced off-target effects. Including multiple independently derived clones per mutation can control for this random genetic drift. Our systematic approach ensures high quality of this publicly available iSCORE-PD resource, highlights the advantages of prime editing over conventional CRISPR/Cas9 methods, and establishes best practices for generating disease-modeling hPSC collections.

## Abstract (web summary)

Researchers generated an isogenic collection of 65 genome-edited hPSC lines with mutations in 11 Parkinson’s disease genes. This resource enables controlled comparisons, improving disease modeling and clarifying how variants drive neurodegeneration.

## Introduction

Parkinson’s disease (PD) is the second most common neurodegenerative disorder with a prevalence of more than 1% in the population over the age of 60^1^. PD is primarily characterized by a progressive loss of dopaminergic neurons in the midbrain and, in most cases, the presence of proteinaceous inclusions (Lewy bodies) in affected cells^2–5^. However, PD-associated pathology is highly variable and can affect a wide range of brain regions^6^. Furthermore, non-neuronal cell types, including astrocytes, oligodendrocytes, and microglia, play important roles in the pathogenesis of the disease^7^. The precise etiology leading to neuronal cell loss is largely unknown. The discovery of mutations in more than 20 genes linked to rare monogenic forms of PD has revealed a broad spectrum of molecular and cellular pathways that can contribute to PD pathology, including vesicle transport, lysosomal function, mitochondrial function, and endoplasmic reticulum (ER) quality control (QC)^4,8^. However, even individuals with PD who carry the same mutation can present with highly heterogeneous clinical and pathological features^5,9^, variable age of onset, and highly diverse or, in some cases, complete absence of Lewy body pathology^6^. Recognizing this variability in penetrance and complex pathology, it is widely acknowledged that additional genetic and environmental modifiers can influence disease pathophysiology, even in monogenic forms of PD^4^. Therefore, distinguishing between common PD-associated phenotypic features from those that are specific to a particular mutation remains challenging.

Genome editing of human pluripotent stem cells (hPSCs), including both human embryonic stem cells (hESCs) and induced pluripotent stem cells (hiPSCs), is increasingly utilized to establish isogenic cellular models for human diseases, such as PD^10–12^. This approach has provided valuable insights into the molecular mechanisms underlying monogenic forms of this disease^11–30^. It utilizes genome editing technologies, such as CRISPR/Cas9 or prime editing systems to generate isogenic cell lines, either by genetically inserting or correcting disease-linked mutations in hPSCs^11,12^. While this approach provides the advantage to analyze the effects of a mutation within a presumably identical genetic background, the extent to which the genomes of edited cell lines, beyond the intended genetic modifications, are truly isogenic remains unclear. Various sources of genetic variability can contribute to genetic alterations in hPSCs. These include off-target effects associated with genome editing, genetic drift during cell culture, and founder effects introduced during subcloning^31–35^. However, to date, we lack a systematic quantification of the relative contribution of these events, raising the important question: to what extent can observed phenotypes be fully attributed to the intended genetic edits, rather than to additional acquired genetic alterations?

In addition, since most isogenic pairs are currently generated in individual hPSCs with distinct, patient-specific genetic backgrounds, cross-comparison of different mutations is confounded by the effect of genetic modifier loci inherent to each individual’s genome^11,36^. Therefore, a unified genetic interaction map of how different monogenic disease-related genes, their pathogenic mutations, and their respective phenotypes interact to drive pathology is still missing. To overcome this challenge, ongoing initiatives for a variety of diseases exist with the goal of streamlining the development of isogenic, disease-relevant hPSC collections derived from a common, thoroughly characterized parental hPSC line^36^. Here, we report the generation of such a resource for PD as part of the Aligning Science Across Parkinson’s (ASAP) research network, which we have termed iSCORE-PD (Isogenic Stem Cell Collection to Research Parkinson’s Disease). We used state-of-the-art genome editing approaches to establish a total of 65 clonal cell lines carrying disease-causing or high-risk PD-associated mutations in 11 genes (*SNCA*, *PRKN*, *PINK1*, *DJ1/PARK7*, *LRRK2*, *ATP13A2*, *FBXO7*, *DNAJC6*, *SYNJ1*, *VPS13C*, and *GBA1*), along with isogenic control lines. All cell lines were derived from a well-characterized and fully sequenced female hESC line (WIBR3; NIH approval number NIHhESC-10-0079)^37^ and underwent rigorous QC. Importantly, we performed whole-genome sequencing (WGS) on all cell lines to address the fundamental question of isogenicity of genome-edited hPSCs by assessing genetic variability within the iSCORE-PD collection. This collection of isogenic hPSCs is accessible to the community to enable the cross-comparison of disease-related phenotypes and accelerate progress in PD research.

## Results

### Characterization of the hESC line WIBR3

**Fig. 1:**
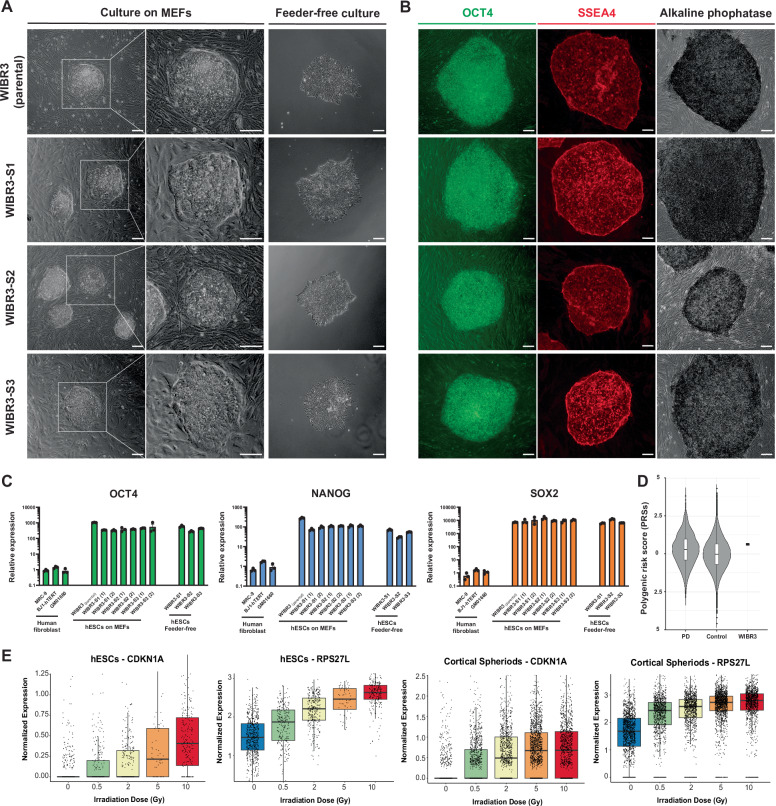
WIBR3 hESC cell line characterization. **A** Representative phase contrast images of parental WIBR3 hESCs and subclones WIBR3-S1, WIBR3-S2, and WIBR3-S3 cultured on MEFs and in feeder-free conditions. Scale bar 100 µm. **B** Immunocytochemistry for pluripotency markers OCT4 (green) and SSEA4 (red), and staining for alkaline phosphatase (black) of WIBR3 (parental) hESCs and subclones WIBR3-S1, WIBR3-S2, and WIBR3-S3 cultured on MEFs. Scale bar 100 µm. **C** qRT-PCR analysis for the relative expression of pluripotency markers OCT4, NANOG, and SOX2 in human primary fibroblasts (MRC-9, BJ1-hTERT, and GM01660), WIBR3 (parental) hESCs, and subclones WIBR3-S1, WIBR3-S2 and WIBR3-S3 hESCs cultured on MEFs and in feeder-free conditions. Relative expression levels were normalized to the expression of these genes in primary fibroblasts. (1) and (2) indicate independent samples. (*N* = 3/group; mean ± SEM; *N* represents technical replicates of the same sample; independent biological replicates are represented side to side in separate bars). **D** Polygenic risk scores (PRSs) for WIBR3 hESCs are compared to the population-centered Z score distribution for PD PRS in 2995 PD cases (PD) and 96,215 normal population individuals (control) from the UK Biobank. Box plots show the median (horizontal line), interquartile range (box; 25th percentile to 75th percentile), whiskers extending to values within 1.5 × IQR, and outliers are shown as individual points. **E** Assessment of p53 pathway activity following irradiation of WIBR3 (parental) hESCs (0 Gy, *N* = 630; 0.5 Gy, *N* = 247; 2 Gy, *N* = 306; 5 Gy, *N* = 59; 10 Gy, *N* = 222; in total 1464 cells) and WIBR3-derived cortical spheroids (0 Gy, *N* = 1307; 0.5 Gy, *N* = 1001; 2 Gy, *N* = 1016; 5 Gy, *N* = 1473; 10 Gy, *N* = 1123; in total 5920 cells) by scRNA-seq analysis for the expression of DNA damage response genes CDKN1A and RPS27L. Box plots show the median (horizontal line), interquartile range (box; 25th–75th percentiles), whiskers extending to values within 1.5 × IQR, and all individual data points, including outliers, are shown. Source data are provided as a Source data file.

A major goal of this work is to complement ongoing initiatives to establish comprehensive collections of hPSC lines that carry mutations associated with PD and related neurodegenerative diseases^36^. The objective is to reduce genetic variability among cell lines to facilitate the identification of disease-relevant pathophysiological signatures. One example of such efforts is the recently described iPSC neurodegenerative disease initiative (iNDI) from the NIH’s Center for Alzheimer’s and Related Dementias (CARD), which utilized the KOLF2.1J (RRID:CVCL_B5P3) hiPSC line^36^. This cell line, derived from a male donor, is currently recognized as a benchmark reference for neurodegenerative disease research and facilitates the comparison of disease-associated phenotypes across different laboratories. Although hiPSCs have proven instrumental for disease modeling, concerns remain regarding the presence of genetic alterations in somatic donor cells before reprogramming, reprogramming-induced genetic alterations, incomplete epigenetic reprogramming, and aberrant genomic imprinting^11,38,39^. Given these considerations, and the necessity for incorporating cells from both sexes, we opted to use the female hESC line WIBR3 of European descent (RRID:CVCL_9767, NIH approval number NIHhESC-10-0079)^37^. This cell line has been previously demonstrated to maintain a stable karyotype over prolonged in vitro culture and has been widely used to model human diseases, including PD^10,30,37,40–45^. For our study, we acquired early passage WIBR3 cells (P14) and initially generated 3 independent single cell-derived subclones (WIBR3-S1, WIBR3-S2, and WIBR3-S3). Both the parental line and its subclones showed regular growth and morphology when cultured on mouse fibroblast feeders (MEFs) and under feeder free conditions in mTeSR™ Plus media (Fig. 1A). We validated the pluripotency of all cell lines through the detection of pluripotency markers using immunocytochemistry and qRT-PCR (*N* = 3/group; mean ± SEM) (Fig. 1B, C, and Supplementary Fig. 1A). Furthermore, we analyzed the genomic integrity of all cell lines using standard array comparative genomic hybridization (aCGH) and a modified high density Illumina infinium global diversity array (GDA) neuro booster array (NBA)^46^. This analysis confirmed a normal karyotype and the absence of larger structural alterations (>~500 kb) in both the parental WIBR3 line and its derived subclones. The complete high-density array genotyping data is available through AMP-PDRD (https://www.amp-pd.org/ via GP2 data sharing agreement).

To determine the presence of insertions and deletions (indels) at higher resolution and to identify potential pathogenic single-nucleotide variants (SNVs), we performed long-read whole genome sequencing (WGS) combining Pacific Biosciences (PacBio, average coverage 28.39×, median read length 18 kb) and Oxford Nanopore Technologies (Nanopore, average coverage 43×, median read length 84 kb). The initial analysis for structural variants applied the Truvari algorithm^47^ (https://github.com/ACEnglish/truvari) to integrate the PacBio and Nanopore datasets and identified a total of 20,561 high-confidence structural variants in the WIBR3 parental line compared to the reference human genome [GRCh38/hg38] (Supplementary Data 1). Among these, 109 were localized to coding exons, impacting 102 genes (Supplementary Data 1). The number and distribution of these structural variants is comparable to those observed in the general human population^48,49^. Considering our goal to model PD and related neurodegenerative diseases, we determined that none of these structural variants affect genes with known pathogenic mutations in PD, Alzheimer’s disease (AD), and AD-related dementias, or risk genes identified in GWAS associated with these diseases^4,50–52^ (Supplementary Data 1). Additionally, we annotated the integrated structural variant calls using SvAnna^53^(https://github.com/TheJacksonLaboratory/SvAnna) to determine if any variant was of high priority for the phenotype terms HP:0002180 (neurodegeneration) and HP:0000707 (abnormality of the nervous system). None of the structural variants analyzed received high priority scores for either term.

Next, we identified the number and distribution of coding, missense, frameshift, and predicted loss-of-function (LOF) SNVs in the parental WIBR3 cell line compared to the reference human genome [GRCh38/hg38] (Supplementary Fig. 1B). This analysis identified 6613 missense SNVs in 3933 coding genes and 120 potential LOF mutations (including 15 start-loss, 54 stop-gain, 13 stop-loss, 18 frameshift deletion, and 20 frameshift insertion). A full list of variants can be found in Supplementary Data 2, and the full genome is available for broad data sharing at AMP-PDRD (https://www.amp-pd.org/ via GP2 data sharing agreement). Overall, the number and distribution of these variants are comparable to that observed in other sequenced hPSC lines^36^ and within the human population found in the gnomAD database (https://gnomad.broadinstitute.org/)^54,55^.

To determine the presence of potentially pathogenic variants in the parental WIBR3 cell line, we annotated all SNVs using ClinVar^47^ (RRID:SCR_006169; https://www.ncbi.nlm.nih.gov/clinvar/). Collectively, these analyses revealed 47 variants reported as pathogenic or with conflicting interpretations of pathogenicity (Supplementary Data 2). In addition, we assessed the gene dosage sensitivity of the potential LOF mutations using the Clinical Genome Resource (www.clinicalgenome.org) and identified four variants with evidence of affecting dosage-sensitive genes or being located within dosage-sensitive genomic regions (Supplementary Data 2). Importantly, none of these potential pathogenic SNVs or dosage sensitive LOF mutations mapped to genes included in the KEGG signatures for Parkinson’s or AD, or to the phenotype terms HP:0001300 (Parkinsonism), HP:0002511 (AD) and HP:0002180 (neurodegeneration) (Supplementary Data 2), except for a heterozygous variant in TREX1 (rs760838030; NM_033629.6(TREX1):c.340C>T [p.Arg114Cys]). This missense mutation has been reported in a homozygous or compound heterozygous state in individuals with autosomal-recessive Aicardi–Goutières syndrome or chilblain lupus, autoinflammatory syndromes that can manifest with cerebral vasculitis^56^.

Because the ClinVar database does not provide pathogenesis information for all SNVs, we also assessed potential pathogenic or deleterious variants using bioinformatics-based predictor tools (Supplementary Data 2). REVEL (rare exome variant ensemble learner)^57^, a machine-learning–based pathogenicity predictor specifically designed to evaluate missense mutations, that integrates commonly used pathogenicity scores and conservation metrics into one model (e.g., PolyPhen-2, MutationTaster, SIFT, MutPred, and CADD, etc.), identified 55 variants with potential pathogenicity (cutoff ≥ 0.50, excluding variants classified as benign in ClinVar). In addition, using CADD (combined annotation-dependent depletion)^58^, a widely used broader and less stringent predictor, we identified 833 potentially deleterious variants (CADD-PHRED ≥ 20, excluding variants classified as benign in ClinVar), the majority of which are common (gnomAD AF ≥ 0.01). Importantly, none of the REVEL or CADD- nominated variants mapped to genes included in the KEGG signatures for PD or AD, or to the phenotype terms HP:0001300 (Parkinsonism) or HP:0002511 (AD). Only two additional common, and therefore unlikely pathogenic variants, one in UQCRHL (rs61744357, gnomAD = 0.1242) and one in RREB1 (rs1334576, gnomAD = 0.427), with CADD-PHRED ≥ 20 but REVEL < 0.5, were linked to the phenotype term HP:0002180 (neurodegeneration) (Supplementary Data 2). Overall, this analysis suggests that the parental WIBR3 cells do not harbor genetic variants that would substantially limit their use in modeling neurodegenerative diseases.

As we aim to provide our cell collection to study PD and related neurodegenerative diseases, we analyzed WGS data to calculate the polygenic risk score (PRS) based on the cumulative number of GWAS risk variants associated with PD (Fig. 1D)^50^. The analysis indicated that the PRS of the parental WIBR3 line falls within the range observed in the normal population. Subsequently, we focused on identifying high-risk variants in known neurodegenerative disease-associated genes. This analysis revealed that WIBR3 is heterozygous for the *APOE* ε4 allele (worldwide allele frequency of *APOE* ε4 ~14%), which is a risk factor for AD^59^, heterozygous for *rs3173615* (TMEM106B p.T185S), which has been reported to be a modifier of frontotemporal dementia^60^, and homozygous for the *MAPT* H1 allele, which is a gene of interest in several neurodegenerative diseases^61,62^.

It is widely recognized that hPSCs can accumulate genetic alterations over time, which provide a growth advantage in cell culture. Notably, mutations in the p53 tumor suppressor pathway have been frequently observed in various hPSC lines^63–65^. To evaluate the function of the p53 pathway in the parental WIBR3 cell line, we analyzed the p53-dependent DNA damage response following irradiation (Fig. 1E). This analysis confirmed a robust p53-mediated response, as indicated by the dose-dependent expression of the DNA damage response genes CDKN1A and RPS27L in both undifferentiated hPSCs and in vitro-derived cortical spheroids (Fig. 1E).

### WIBR3 cells differentiate into PD-relevant cell types

**Fig. 2:**
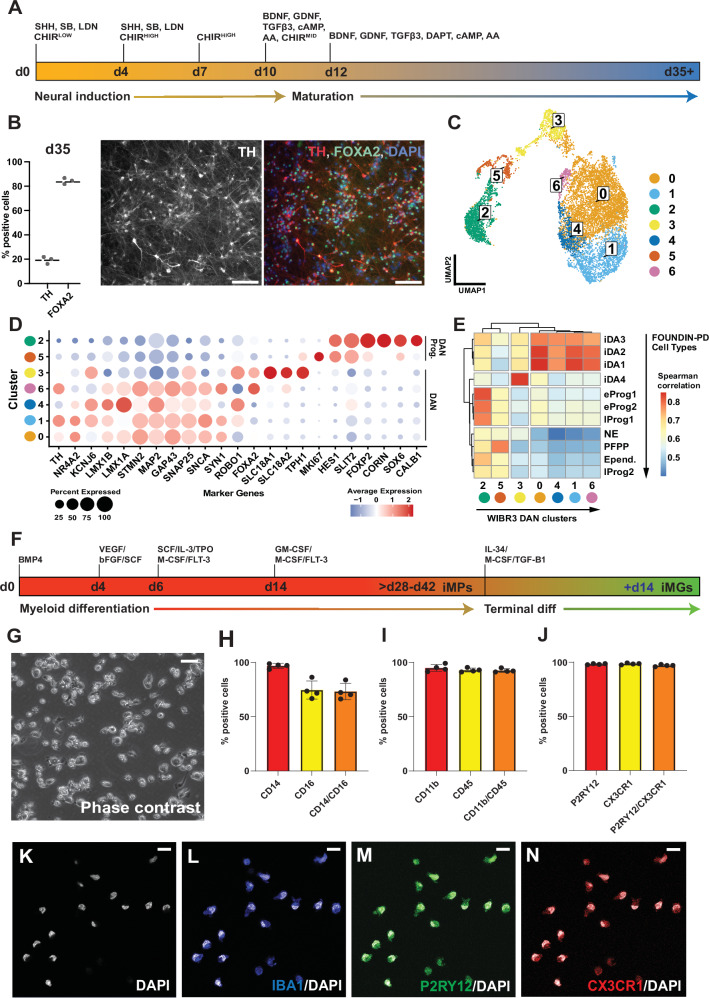
WIBR3 differentiation potential into dopaminergic neurons and microglia in 2D culture. **A** Schematic depicting the protocol for in vitro differentiation of dopaminergic neurons from WIBR3 hESCs. **B** Immunocytochemistry and quantification of TH and FOXA2 expressing cells in WIBR3 (parental) hESC-derived dopaminergic neurons at day 35. Scale bar 100 µm. (*N* = 3; *N* represents biological replicates of individual clones WIBR3-S1,2,3). Check Supplementary Fig. 2A for immunocytochemistry images from WIBR3-S1,2,3. **C** Uniform manifold approximation and projection (UMAP) plot of scRNA-seq analysis at day 35–37 of dopaminergic neuron differentiation from WIBR3-S1, WIBR3-S2, and WIBR3-S3 hESCs, showing 10,097 cells separated into 7 coarse clusters. **D** Dot plot showing expression of key progenitor and mature dopamine neuron marker genes across different cluster identities indicates that clusters identified in (**C**) represent dopamine neuronal progenitors and dopaminergic neurons at different developmental stages. Dot size indicates the proportion of cells in a cluster expressing a given gene, while color intensity indicates its average expression. Although cluster 2 showed *CALB1* expression, we labeled these cells a progenitor population due to the expression of *HES1, SLIT2, CORIN,* and the absence of mature dopamine neuron markers. **E** Heatmap depicting Spearman correlation coefficients between pseudo-bulk expression profiles of 7 WIBR3 clusters identified in (**C**) compared to pseudo-bulk expression profiles of 11 FOUNDIN-PD cell types. **F** Schematic depicting in vitro microglia differentiation protocol from WIBR3 hESCs. **G** Representative phase contrast image of iMGs derived from subclone WIBR3-S1 (terminal differentiation day 14; scale bar 50 µm). Quantification of flow cytometry (FACS) analysis described in Supplementary Fig. 5A for the expression of CD14/CD16 and CD11b/CD45 in iMPs (**H**, **I**) and P2RY12/CX3CR1 in matured iMGs (**J**) derived from the parental WIBR3 hESCs and subclones WIBR3-S1, S3, S3 (*N* = 4; mean ± SD; *N* represents biological replicates WIBR3(parental) and clones WIBR3-S1,2,3). **K**–**N** Representative immunostaining images of in vitro differentiated iMGs derived from subclone WIBR3-S2 showing microglia-specific markers IBA1, P2RY12, and CX3CR1, along with the nuclear marker DAPI (terminal differentiation day 14; scale bar, 10 µm). Source data are provided as a Source data file.

Given that PD is characterized by the chronic progressive loss of dopaminergic neurons in the substantia nigra, the effective generation of these cells is crucial for in vitro modeling of PD. To address this, we implemented a previously established protocol^66,67^ to differentiate the three independently derived WIBR3 subclones (WIBR3-S1, WIBR3-S2, and WIBR3-S3) into midbrain-specified dopaminergic neurons. Briefly, WIBR3 hESCs underwent neural induction via dual SMAD inhibition, combined with sonic hedgehog (SHH) agonist exposure and biphasic WNT activation using the GSK-3 inhibitor CHIR99021, resulting in robust midbrain-specific patterning within the first 11 days. From day 12 onwards, committed midbrain neural progenitors were differentiated into dopaminergic neurons until day 35 using a cocktail to promote terminal differentiation (BDNF, GDNF, TGFß3, DAPT, cAMP, and ascorbic acid) (Fig. 2A). For each subclone, we determined the efficacy of neural induction into neural precursor cells and dopaminergic neurons by analyzing the expression of midbrain-specific markers by immunocytochemistry (TH and FOXA2) and qRT-PCR (FOXA2, LMX1A, NR4A2, KCNJ6, TH, PITX3, EN2, AADC, and SYN1) (Fig. 2B and Supplementary Fig. 2). At early time points (day 11 and day 25), the in vitro differentiated cultures expressed canonical midbrain floor plate genes at levels comparable to those in concurrently differentiated KOLF2.1J hiPSCs (Supplementary Fig. 2B). At day 35 of differentiation, over 80% of cells expressed FOXA2, a marker for early midbrain floorplate neuronal precursors, and approximately 20% expressed the dopaminergic neuron marker tyrosine hydroxylase (TH), indicating the generation of midbrain-specified dopaminergic neurons (Fig. 2B and Supplementary Fig. 2A).

To further characterize the dopaminergic neuron cultures, we performed single-cell RNA sequencing (scRNA-seq) at day 35–37 post-differentiation from hESCs. Cells were profiled across the three independently assayed subclones, yielding an aggregate dataset of 10,097 cells. Using uniform manifold approximation and projection (UMAP) for dimensionality reduction, seven distinct clusters were identified in the integrated dataset, each composed of cells from all three subclones (Fig. 2C and Supplementary Fig. 3A). Among these, clusters 0, 1, 3, 4, and 6 showed strong expression of canonical dopaminergic neuron markers (*KCNJ6, TH, NR4A2*), whereas clusters 2 and 5 displayed strong expression of dopaminergic neuronal progenitor markers (*SLIT2, FOXP2*, *CALB1, SOX6, and CORIN*) (Fig. 2D and Supplementary Fig. 3B–D)^68,69^. To further compare the differentiation propensity of WIBR3 cells against other cell lines with different genetic backgrounds, we compared our dataset with the recently published Foundational Data Initiative for Parkinson’s disease (FOUNDIN-PD) data (Fig. 2E)^69^. The FOUNDIN-PD dataset includes scRNA-seq data from midbrain dopaminergic neuron cultures at day 65, derived from 80 distinct hiPSC lines using a comparable in vitro differentiation protocol^69^. This comparison revealed that the clusters representing dopaminergic neuron populations (clusters 0, 1, 3, 4, and 6) in our dataset showed the highest Spearman correlation scores with iDA1, iDA2, iDA3, and iDA4 neuron clusters identified in the FOUNDIN-PD data, and clusters 2 and 5 from our dataset correlate more strongly with progenitor populations (Supplementary Fig. 4). Together, this analysis indicates a high similarity between the expression profiles of our WIBR3-derived cell types and those in the FOUNDIN-PD dataset, which is currently the most comprehensive and standard data set for in vitro-derived midbrain-specific dopaminergic neurons. This is relevant, as it should allow the integration of data generated from the iSCORE-PD collection with the FOUNDIN-PD datasets, which include 80 hiPSC lines from patients with sporadic and familial PD, as well as age-matched healthy individuals.

Recent data highlighted that the impact of PD-associated mutations extends beyond neurons, affecting cell types, such as microglia, which play a critical role in the pathogenesis of PD^70,71^. Chronic microglial activation is suggested to be a key pathophysiological feature of many neurodegenerative disorders, including PD^72^. Therefore, we followed a previously described protocol^73^ to differentiate the parental WIBR3 and its subclones (WIBR3-S1, WIBR3-S2, and WIBR3-S3) into microglia-like cells (iMGs). In this protocol, hPSCs were initially induced to myeloid intermediates and subsequently differentiated into microglia-like cells through the addition of cytokines, normally secreted from neurons and astrocytes including IL-34, M-CSF, and TGF-β1 (Fig. 2F). All WIBR3 subclones robustly generated microglial precursors (iMPs) and iMGs, evidenced by their morphology (Fig. 2G) and presence of a high percentage of cells expressing markers tied to the microglial lineage (92.5% CD11b/CD45 double positive and 73.2% CD14/CD16 double positive) (Fig. 2H, I, and Supplementary Fig. 5). Moreover, terminal differentiation yielded a high percentage of cells expressing key markers for mature microglia, such as IBA1, CX3CR1, and P2RY12, as determined by FACS analysis (97% CX3CR1/P2RY12 double positive; Fig. 2J and Supplementary Fig. 5) and immunostaining (Fig. 2K–N and Supplementary Fig. 5). Collectively, these findings underscore the suitability of WIBR3 hESCs as a model to study the contribution of different cell types to PD pathology.

### Genetic engineering of PD-associated mutations requires multiple editing modalities, including prime editing

**Fig. 3:**
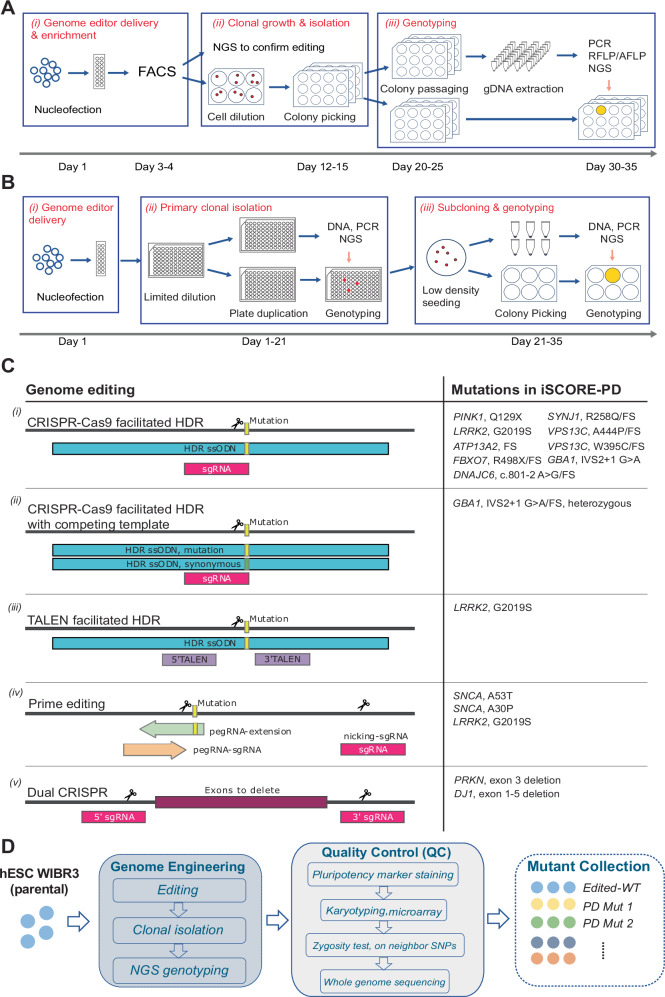
Gene editing workflow to generate iSCORE-PD collection. **A** Schematic illustrating the genome editing pipeline A. This approach involves i FACS-based enrichment of nucleofected cells containing the gene editing reagents, including a fluorescent reporter, ii the isolation of clonally expanded cell lines, and iii the NGS-based genotyping to identify correctly edited cell lines. **B** Schematic illustrating genome editing pipeline B. This approach utilizes a high-throughput cell isolation system. This approach includes i nucleofection of the gene editing reagents, ii the plating of cells in a limited dilution (~10 cells/well) to isolate wells containing correctly targeted cells by NGS, and iii subcloning, expansion, and NGS-based genotyping to isolate correctly targeted clonal cell line. **C** Table summarizing the gene editing strategies used to generate the iSCORE-PD collection. These include: i CRISPR/Cas9 facilitated homology directed repair (HDR) using ssODNs containing the desired genetic modification as a repair template for CRISPR/Cas9 induced double strand break. ii The use of competing HDR templates (ssODNs) containing synonymous mutations in the sgRNA-target site to favor the generation of heterozygous over homozygous mutations. iii TALEN-facilitated HDR using ssODNs containing the desired genetic modification as a repair template for CRISPR/Cas9-induced double-strand break. iv Prime editing approach to insert the PD-associated point mutations into hESCs*.* v Dual CRISPR approach using 3**′** and 5**′** sgRNAs flanking the desired deletion to recreate large genomic structural alterations identified in PD patients. **D** Overview depicting genome engineering and quality control steps in the generation of the iSCORE-PD collection.

The establishment of a large-scale collection of cell lines carrying disease-associated mutations requires precise and robust gene-editing approaches to insert the desired genetic alterations in hPSCs. We previously demonstrated that WIBR3 cells can be genetically modified with high efficiency using either CRISPR/Cas9, TALEN, or prime editing-based genome engineering approaches^40,74,75^. CRISPR/Cas9-based editing is effective for introducing targeted genomic deletions and biallelic alterations, while prime editing is highly efficient in introducing heterozygous modifications, which is necessary for modeling dominantly inherited disease-associated alleles^74^. To create cell lines carrying PD-associated genetic alterations in WIBR3 hESCs, we employed two different editing pipelines. Pipeline A (Fig. 3A) utilizes FACS-enrichment post-nucleofection to purify effectively transfected cells, followed by clonal expansion and genotyping to allow the isolation of correctly targeted lines. The estimated time for the editing pipeline A is 30–35 days. Pipeline B (Fig. 3B) uses nucleofection, limited dilution, and next-generation sequencing (NGS)-based genotyping to identify desired edits in a 96-well plate format. This integrated workflow allows for the efficient isolation and purification of correctly edited clonal cell lines, even at low frequency, within a shorter time frame compared to previous approaches (21–35 days).

Given the identification of several disease-causing mutations within most PD-linked genes, the selection and prioritization of specific alleles for gene editing within each gene were based on confidence in pathogenicity for each mutation, allele prevalence, and feasibility of the editing strategy. As described in detail in the supplementary information for each gene (Supplementary Note 1 and Supplementary Figs. 6–17), we employed three general editing strategies to closely recreate the genomic alterations identified to be causal or high-risk factors for PD. These editing strategies include (1) the precise insertion of point mutations using CRISPR/Cas9, TALEN or prime editing approaches to recreate specific PD-associated missense mutations (heterozygous and/or homozygous), (2) the insertion of small indels to create frameshift (FS) or premature stop mutations using CRISPR/Cas9, and (3) a dual CRISPR/Cas9 approach using 3′ and 5′ single-guide RNAs (sgRNAs) flanking the target region to create genomic deletions identified in PD patients (Fig. 3C).

### iSCORE-PD: a cell line collection of isogenic hPSC lines carrying PD-associated mutations

To establish iSCORE-PD, a collection of isogenic hPSC lines carrying monogenic or high-risk variants linked to PD, we initially prioritized engineering mutations in high-confidence PD genes^4^. The specific modifications for each gene were selected based on information in the MDSgene database^76^ (https://www.mdsgene.org) and the currently available literature, as outlined in detail for each gene in the supplementary information (Supplementary Note 1). Overall, the current iteration of the iSCORE-PD collection includes 65 clonal cell lines carrying high-risk or causal variants in 11 genes linked to PD (*SNCA* A53T, *SNCA* A30P, *PRKN* Ex3del, *PINK1* Q129X, *DJ1/PARK7* Ex1-5del, *LRRK2* G2019S, *ATP13A2* FS, *FBXO7* R498X/FS, *DNAJC6* c.801A>G/FS, *SYNJ1* R258Q/FS, *VPS13C* A444P/FS, *VPS13C* W395C/FS, *GBA1* IVS2+1/FS) and isogenic control lines. All cell lines (Table 1 and Supplementary Data 3) passed all the below-described QC steps (Fig. 3D) and will be available to the scientific community through the WiCell Research Institute (https://www.wicell.org/). A detailed discussion for each cell line can be found in the Supplementary information (Supplementary Note 1, Supplementary Data 3, and Supplementary Figs. 6–17).

**Table 1:** iSCORE-PD cell line collection (overview)

							Cells in collection	
	Mutation	Inheritance	Frequency^b^	Proposed disease mechanism^b^	Note^b^	Editing	Monoallelic - heterozygous	Biallelic - homozygous
*(PARK1) SNCA*	A53T	Autosomal dominant	Very rare	Gain of function	Often with dementia	Prime editing	3	–
	A30P					Prime editing	3	1
*(PARK2) PRKN*	EX3DEL	Autosomal recessive	Rare	Loss of function	Often early onset	CRISPR/Cas9 (dual guide)	–	3
*(PARK6) PINK1*	Q129X	Autosomal recessive	Rare	Loss of function	Often early onset	CRISPR/Cas9 (HDR)	–	3
*(PARK7) DJ1*	EX1-5DEL	Autosomal recessive	Very rare	Loss of function	Often early onset	CRISPR/Cas9 (dual guide)	5	7
*(PARK8) LRRK2*	G2019S	Autosomal dominant	Common	Gain of function	–	Prime editing, TALEN, and CRISPR/Cas9 (HDR)	4	1
*(PARK9) ATP13A2*	FS	Autosomal recessive	Very rare	Loss of function	Atypical PD	CRISPR/Cas9 (HDR)	–	4
*(PARK15) FBXO7*	R498X/FS	Autosomal recessive	Very rare	Loss of function	Often early onset	CRISPR/Cas9 (HDR)	–	2
*(PARK19) DNAJC6*	c.801-2A>G/FS	Autosomal recessive	Very rare	Loss of function	Often early onset	CRISPR/Cas9 (HDR)	–	2
*(PARK20) SYNJ1*	R258Q/FS	Autosomal recessive	Very rare	Loss of function	Often atypical PD	CRISPR/Cas9 (HDR)	–	2
*(PARK23) VPS13C*	W395C/FS	Autosomal recessive	Rare	Loss of function	–	CRISPR/Cas9 (HDR)	–	4
	A444P/FS	Autosomal recessive				CRISPR/Cas9 (HDR)	1	2
*GBA1*	IVS2+1/FS	Autosomal dominant^a^	Common	Likely loss of function	–	CRISPR/Cas9 (HDR)	4	2
Wild type	–	–	–	–	–	–	Parental + 3 independent clones	
Edited wild type (EWT)	–	–	–	–	–	Prime editing, CRISPR/Cas9 (HDR)	Prime editing (6) + CRISPR/Cas9 (2)	

^a^Mixed inheritance.

^b^Adapted from Blauwendraat et. al. 2019^4^.

An important consideration in hPSC-based disease modeling is the selection of appropriate control lines. To address this, we provide a set of subclones derived from the parental WIBR3 cell line (WIBR3-S1, S2, S3) (Fig. 1 and Supplementary Fig. 1). Additionally, we included WIBR3 cell lines that were isolated as part of the standard genome editing experiment but did not exhibit any genetic modifications at the targeted locus, referred to as “edited wildtype” (EWT) cells. We consider these cells as preferred experimental controls, as they most effectively account for any nonspecific changes induced by the gene-editing process. The EWT cell lines include: EWT_S1-3 (prime editing controls-pipeline B), EWT_S4-5 (CRISPR/Cas9 controls-pipeline B), and EWT_S6-8 (prime editing controls-pipeline A) (Table 1, Supplementary Note 1, Supplementary Data 3, and Supplementary Fig. 6).

### Generation of hPSC collections requires rigorous quality control

A significant challenge for any genome editing approach is the risk of introducing unintended on- and off-target genetic modifications in the edited cell lines. Additionally, it is well-established that clonal expansion and in vitro culture of hPSCs can lead to the acquisition of genetic alterations that can provide growth advantages^34,63–65,77–79^. Consequently, there is a consensus in the field that gene-edited hPSC-derived disease models should undergo a rigorous QC process to validate the pluripotency of edited cell lines, and to ensure the absence of major gene editing- or culture-induced genetic alterations. As part of this collection, all genome-edited cell lines underwent a comprehensive QC process, as outlined in Fig. 3D. Following genome editing and subsequent clonal expansion, individual correctly targeted clones were initially identified using either Sanger sequencing or NGS. All correctly targeted cell lines were subsequently expanded, cryopreserved at a low passage, and assayed by immunocytochemistry for the expression of pluripotency markers OCT4, SSEA4, and alkaline phosphatase. To confirm a normal karyotype and assess overall genomic integrity, genome-edited clonal hESC lines underwent standard aCGH karyotyping and were analyzed using a modified high-density Illumina Infinium GDA NBA^46^. This analysis aimed to identify cell lines with large genome editing-induced structural alterations or complete chromosomal loss compared to the genome of the parental WIBR3 cell line. The complete high-density array genotyping data is available through AMP-PDRD (https://www.amp-pd.org/ via GP2 data sharing agreement).

A frequently overlooked challenge associated with genotyping approaches based on PCR amplification of the target locus is the common failure to detect loss of heterozygosity (LOH)^80^. LOH results from large deletions or the loss of entire chromosome fragments distal to the site targeted during the genome-editing process. To rule out LOH in cell lines that appeared to be homozygously edited based on the detection of a single allele by NGS, we introduced an additional QC step. We used either a Southern blot or SNV-PCR-based analysis to validate the presence of two alleles at the targeting site, as described in detail for each gene in the supplementary information (Supplementary Note 1, Supplementary Data 3, and Supplementary Figs. 6–17). Using this analysis, we identified LOH in 2 out of 18 (11.11%) tested clonal cell lines initially classified as correctly edited with two identical alleles at the target site. These data underscore that LOH is a significant complication arising from genome editing and emphasize the importance of incorporating LOH testing as an important component of the QC process for genome-edited hPSC lines. Any cell lines showing alterations in any of the QC assessments described above were removed from the collection.

A summarized list of the genes, mutations, and number of cell lines in the iSCORE-PD collection is provided in Table 1. For detailed information regarding the gene editing process and QC of all analyzed clonal cell lines in the generation of the iSCORE-PD collection, see Supplemental Note 1, Supplementary Data 3, 4, and Supplementary Figs. 6–17. Overall, 19.75% (16 out of 81) of isolated clonal cell lines with correct NGS-confirmed genotype were excluded from our collection. As summarized in Supplementary Data 4, reasons for exclusion include chromosomal and structural alterations (16.05%—13 out of 81 lines analyzed), lack of pluripotency marker expression (1.47%—1 out of 68 lines analyzed), and LOH (11.11%—2 out of 18 lines analyzed). It is important to note that the frequency of chromosomal and large structural abnormalities was higher in clones generated by double-strand break-based genome editing (CRISPR and TALEN, 20.34%—12 out of 59 lines analyzed) compared to prime editing (5.56%—1 out of 18 lines analyzed). Similarly, LOH at the targeted locus was only observed in CRISPR/Cas9 edited cell lines and was absent in prime edited cell lines.

### Genetic variability between cell lines in the collection is largely driven by preexisting spontaneous mutations

**Fig. 4:**
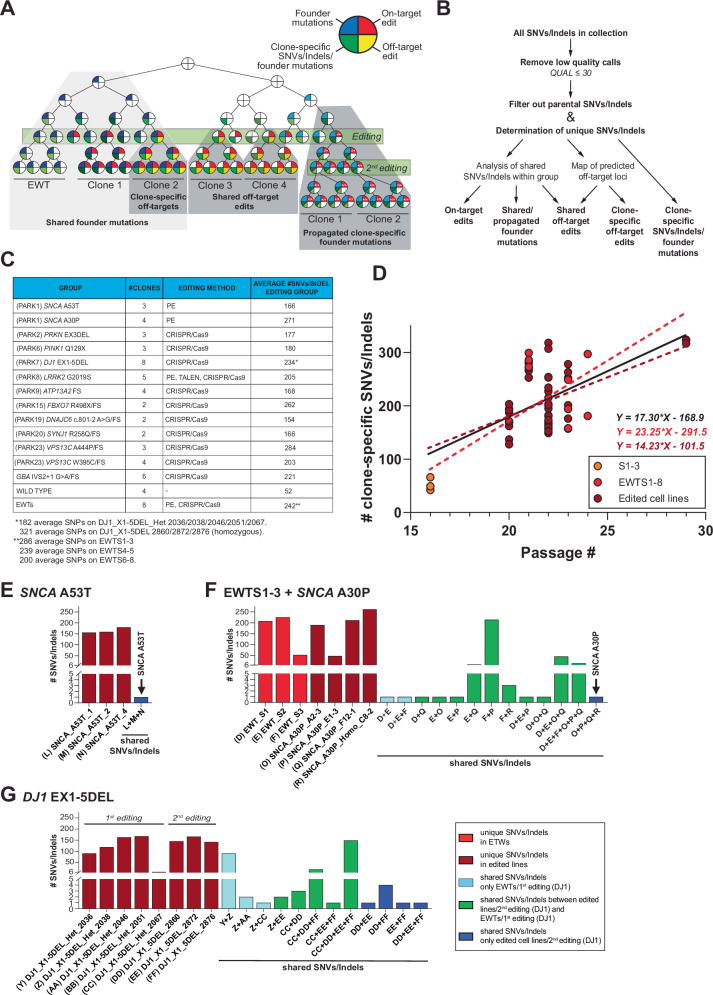
Genetic variation in the iSCORE-PD collection. **A** Schematic representation of the potential source of genetic variability found in the iSCORE-PD collection, including shared and clone-specific founder mutations (SNVs/Indels), propagated founder mutations, and genome editing-induced variations (on- and off-target edits). For clarity of illustration, different shades of blue, green, and yellow represent distinct sets of mutations. **B** Schematic of the WGS analysis pipeline to identify unique and shared SNVs/indels for each editing group. **C** Table of all editing groups in the iSCORE-PD collection and SNVs/Indels related information: number of clones, editing method used to generate them, and average number of SNVs/indels per cell line in each editing group. **D** Correlation between the number of clone-specific SNVs/Indels and passage number determined based on the last clonal event for each cell line. Orange dots indicate parental subclone lines (WIBR3-S1,2,3), light red dots indicate WIBR3_EWTS1-8, and dark red dots indicate all other edited cell lines. Indicated regression lines are calculated based on all cell lines (solid black line; *y* = 17.3x-168.9; *R*^2^: 0.3820; 95% CI: 11.52–23.08; *N* = 60), edited cell lines only (dark red dotted line; *y* = 14.23x-101.5; *R*^2^: 0.2801; 95% CI: 7.537–20.93; *n* = 49) or control lines only (WIBR3_S1-3 and WIBR3_EWT_S1-8; light red dotted line; *y* = 23.25x-291.5; *R*^2^: 0.5564; 95% CI: 7.597–38.90; *N* = 11). **E** Graph showing the number of unique and shared SNVs/indels in the SNCA A53T group. **F** Graph showing the number of unique and shared SNVs/indels in the SNCA A30P group and EWT1-3 isolated from the same editing experiment (editing group). Shared SNVs/indels show evidence for pre-existing mutations in the parental cell line prior to editing (founder mutations). **G** Graph showing the number of unique and shared SNVs in the *DJ1/PARK7* editing group. Data show a significant clonal founder effect between DJ1_X1-5del_Het_2067 and clones DJ1_X1-5del_2860/2872/2876 (compare Supplemental Fig. 10). Different color bars indicate unique or shared SNVs/Indels between different cell lines. Dark red indicates unique SNVs/Indels in edited lines, light red indicates unique SNVs/Indels in EWT lines, light blue indicates shared SNVs/Indels only found on EWTs/1st editing (*DJ1/PARK7*), green indicates shared SNVs/Indels between edited cell lines/2nd editing (*DJ1/PARK7*) and EWTs/1st editing (*DJ1/PARK7*) and dark blue indicates shared SNVs/Indels only found in edited cell lines/2nd editing (*DJ1/PARK7*). Source data are provided as a Source data file.

Various sources of genetic alterations, beyond the intended genome edits, can contribute to genetic variability in hPSCs that potentially can affect the phenotypic analysis of hPSC-derived cells. As outlined in Fig. 4A, genetic variation can arise from either spontaneous mutations that result from imprecise DNA replication or DNA repair after damage^34,81^, as well as from nonrandom off-target effects associated with genome editing^32,33,82^. As most of these mutations, similar to somatic mutations found ubiquitously across normal tissues, do not strongly impact cellular fitness^81^, cell lines comprise a complex mosaicism of subpopulations with fluctuating allele frequencies that are subject to genetic drift during cell culture, and founder effects generated during subcloning. This inherent genetic variability raises a fundamental question for hPSC-derived disease models: how confidently can we attribute observed phenotypes to the intended genetic edits, rather than to additional acquired genetic alterations?

To comprehensively characterize the genomic variability within the iSCORE-PD collection, we performed WGS on the majority of cell lines in the collection (*n* = 61) and developed a novel analysis pipeline (Fig. 4B). The complete WGS data is available through the Aligning Science Across Parkinson’s Collaborative Research Network (ASAP CRN) Cloud (cloud.parkinsonsroadmap.org under the ASAP CRN Cloud Data Use Agreement). Using DeepVariant (https://github.com/google/deepvariant)^83^ for variant calling and Glnexus for joint-genotyping^84^, we mapped all cell line-specific variants (SNVs and indels) relative to the reference genome (GRCh38/hg38). Each genome-edited line was compared to the WIBR3 genome (see “Materials and Methods” for details) to identify all clone-specific SNVs/indels that are not found in the parental cell line (Fig. 4B). This analysis revealed that the genome-edited cell lines in the iSCORE-PD collection, including non-edited controls (WIBR3_EWTS1-8), carry an average of 216.1 ± 55.5 (mean ± SD) SNVs/indels (Fig. 4C and Supplementary Data 5). As validation for the robustness of this approach for detecting novel SNVs/indels, our analysis pipeline identified all but one engineered mutation (WIBR3_DNAJC6_FS_FS_H10_1) in the clonal cell lines (Supplementary Data 5 and 6). Of the identified SNVs/indels other than the targeted mutations, 1.3 ± 1.3 (mean ± SD, excluding synonymous mutations) variants per cell line were localized to protein-coding exons (Supplementary Data 6). Importantly, protein-coding SNVs in only five genes (including synonymous variants as described in detail below) were shared among multiple correctly genome-edited cell lines. Notably, the number of SNVs/indels in each clonal cell line showed a positive correlation with passage number (*R*² =  0.3820) (Fig. 4D), indicating that overall, the clonal lines acquire an estimated 17.3 (95% CI: 11.52–23.08) mutations per passage during standard cell culture. This rate aligns with previously reported numbers for hPSCs^34^. Importantly, SNV/indel numbers in the non-edited control lines (WIBR3-S1-3 and EWT_S1-8) showed a similar trend compared to the genome-edited cell lines (Fig. 4D), indicating that the editing process had minimal impact on the total number of SNVs/indels per cell line.

### Common genetic variants are rare and, when controlled for, do not confound the phenotypic analysis

An important question is whether genome editing introduces common, nonrandom genetic variation into engineered cell lines. To address this, we compared the SNVs/indels in all clonal cell lines isolated from each targeting experiment to edit a specific PD-associated mutation (referred to as editing group) to distinguish unique versus shared variants among the cell lines within each editing group. For some editing groups (*SNCA*-A53T, *DNAJC6*, *SYNJ1*, and *VPS13C* W395C), we found no shared SNVs/indels among the cell lines, apart from the engineered mutation itself (Fig. 4E, Supplementary Fig. 18H, I, K, and Supplementary Data 5). However, in the remaining groups (*LRRK2*, *SNCA*-A30P, *PRKN*, *PINK1, DJ1/PARK7*, *ATP13A2*, *FBX07, VPS13C* A444P, and *GBA1*), we identified some shared SNVs/indels between cell lines (Fig. 4F, G, Supplementary Fig. 18, and Supplementary Data 5).

As outlined above, two primary sources of genetic variability in genome-edited clonal lines are: (1) SNVs/indels that arise within the founder cell population prior to editing and become fixed due to targeting-associated clonal expansion (founder mutations), and (2) nonrandom genome editing-associated off-target effects. The high number of shared SNVs/indels (up to 215) between individual clonal cell lines strongly suggests that the founder mutations in the parental cell line prior to gene editing are the predominant source of common variants. If this hypothesis is correct, common SNVs/indels should be shared between edited clones and non-targeted controls from the same targeting experiment. Indeed, we observed a significant overlap of SNVs/indels between correctly targeted clones and non-targeted controls derived from the same experiments in the *SNCA*-A30P and *GBA1* editing groups (Fig. 4F and Supplementary Fig. 18L). Supporting the effect of founder mutations, clones with shared SNVs/indels exhibited significantly fewer unique variants (Fig. 4F, e.g., comparing EWT_S3 and SNCA-A30P_E1-3). Together, these data suggest that the shared SNVs/indels represent a significant subset of the variations typically acquired during cell culture prior to gene editing, rather than additional variability introduced by the editing process.

Of note, all initially analyzed *DJ1/PARK7* homozygous clones (WIBR3_DJ1_X1-5DEL_2860/2872/2876) share most of their SNVs/indels with the heterozygous WIBR3_DJ1_X1-5DEL_Het_2067 cell line. This is a direct consequence of the targeting strategy, as these homozygous clones were generated through two successive rounds of editing (Supplementary Fig. 10). In this approach, the second clonal editing step propagates the clone-specific founder mutations present in the heterozygous parental line (Fig. 4A, G). Notably, the homozygous cell lines acquired an average of 21.6 mutations per passage (~151 unique mutations/7 passages) between the two clonal targeting steps, further supporting our earlier estimates of acquired mutations resulting from standard cell culture.

To account for the potential impact of the shared variants on phenotypical analyses, we screened for an additional homozygous clone that was generated in a single targeting step (WIBR3_DJ1_X1-5DEL_6235). Additionally, we included three homozygous *DJ1/PARK7* clones (WIBR3_DJ1_EX1-5DEL_6348/6390/6407), which were generated by retargeting a second heterozygous cell line (WIBR3_DJ1_X1-5DEL_2046) that did not share SNVs/indels with the previously described homozygous clones (WIBR3_DJ1_X1-5DEL_2860/2872/2876). As there is currently no evidence that heterozygous genotypes confer an increased risk of developing PD^85^, we included several heterozygous DJ1/PARK7 lines as experimental controls (WIBR3_DJ1_X1-5DEL_Het_2036/2038/2046/2051/2067) that should account for the genetic variability of the homozygous targeted *DJ1/PARK7* clones (Supplementary Fig. 10).

Although most SNVs/indels are noncoding, we analyzed the shared variants that affect protein-coding sequences. As summarized in Supplementary Data 6, we identified heterozygous SNVs in the coding sequence of five genes that were shared across multiple clonal lines within specific editing groups and could affect protein function: (1) *SLC25A51* (nonsense mutation) in WIBR3_SNCA-A30P clones A2-3 and F12-1; (2) *SEPTIN10* (non-synonymous mutation) in WIBR3_DJ1_X1-5DEL_Het_2067, WIBR3_DJ1_X1-5DEL_2860, 2872 and 2876 clones; (3) *SLC35A2* (non-synonymous mutation) in WIBR3_VPS13C_A444P_Homo_C8_2 and WIBR3_VPS13C_FS_Homo_H3_1; (4) *SLC9A4* (synonymous) in WIBR3_LRRK2_G2019S_5_Het, and WIBR3_LRRK2_G2019S_6_Het; and (5) *GUCA2B* (synonymous) in WIBR3_VPS13C_A444P_Homo_C8-2 and WIBR3_VPS13C_A444P_Homo_H3-1. Consistent with the concept of propagated founder mutations, the SNV in SLC25A51 was also detected in non-targeted control clones from the same editing experiment (EWT_S1 and EWT_S2), and the SNV in *SEPTIN10* was already present in the heterozygous WIBR3_DJ1_X1-5DEL_Het_2067 parental clone (Fig. 4G). A full description of all SNVs/indels in protein coding regions (splice sites, promoter region, introns, coding exons, 5′ UTR and 3′ UTR) of each cell line in the iSCORE-PD collection is provided in Supplementary Data 7. In no case are unintended variants affecting protein-coding found in all clones within any specific editing group; thus, they are unlikely to confound phenotypic interpretation when all clones for a given gene are assayed for PD-related pathologies alongside proper control cell lines.

### Off-targets are rare in CRISPR/Cas9 edited and absent in prime edited iSCORE-PD clones

**Fig. 5:**
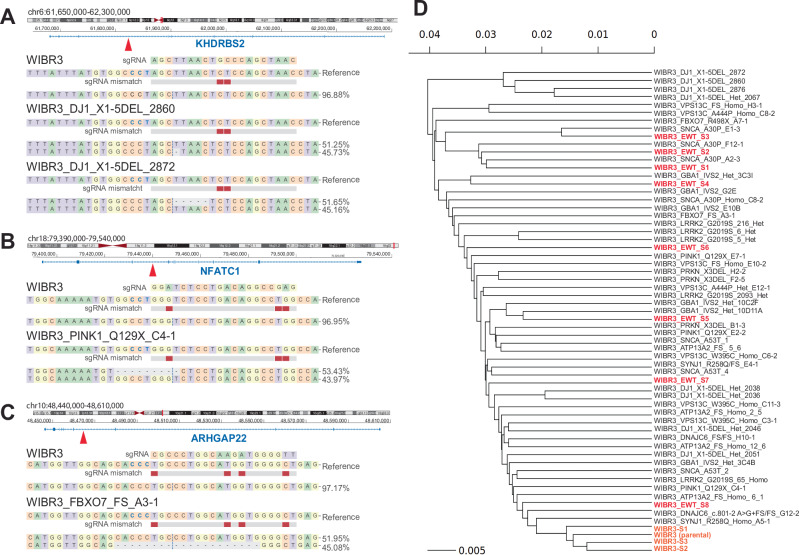
Off-targets and phylogeny. Analysis of off-targets was performed on all gene-edited cell lines and EWT_S1-5. **A**–**C** NGS results of predicted off-target loci (compare Supplementary Data 8) and reference WIBR3 (parental) showing genomic location and nearest gene. All off-target events described are heterozygous, intronic, and noncoding. PAM sequence bases were bolded in blue for clarity. **D** Phylogenetic relationship of all analyzed cell lines in iSCORE-PD. Cell lines displayed in orange represent WIBR3 (parental) + WIBR3-S1,2,3 subclones, and light red represents untargeted EWT_S1-8 cell lines.

Genome editing can induce unintended off-target mutations^32,33,82^; however, the frequency and relevance of these mutations for genetically engineered hPSC-based disease models remain unclear. The above analysis indicates that the genetic variability observed in the iSCORE-PD collection is largely driven by the subcloning process of cells that have spontaneously acquired mutations. Nonetheless, we cannot exclude the potential contribution of off-target effects mediated by CRISPR/Cas9 or prime editing. To investigate potential off-target effects, we used Cas-OFFinder^86^ (RRID:SCR_023390; v2.4.1; https://github.com/snugel/cas-offinder) with a relaxed threshold allowing up to five mismatches to generate a comprehensive list of predicted off-target sites for all sgRNAs and pegRNAs employed in generating the iSCORE-PD lines. We then identified all SNVs/indels within a 100 bp window surrounding these predicted off-target sites for each genome-editing experiment. Despite the low-stringency threshold, this analysis identified only five SNVs/indels near potential off-target sites across 54 assessed cell lines (Supplementary Data 8). Among these, we considered SNVs/indels at three off-target sites in four cell lines (WIBR3_DJ1_X1_5DEL_2860, WIBR3_DJ1_X1_5DEL_2872, WIBR3_FBXO7_FS_A3_1, and WIBR3_PINK1_Q129X_C4_1) as genuine off-target modifications. Consistent with a CRISPR/Cas9-mediated cleavage pattern, these modifications were located 2–5 bases upstream of the NGG protospacer adjacent motif (Fig. 5A–C and Supplementary Data 8). All off-targets resulted in heterozygous modifications. Notably, only one instance of an off-target modification was shared across two cell lines (WIBR3_DJ1_X1_5DEL_2860, WIBR3_DJ1_X1_5DEL_2872). While the number of genuine off-target events was low even in double-strand break-based edited clones (4 in 41 analyzed cell lines), it is important to note that no off-targets were detected in prime-edited cell lines. This is consistent with the above-described observation that prime editing induced less SNVs and LOH at the editing site. Thus, off-target effects can occur in genome-edited cell lines, particularly when using the CRISPR/Cas9 system, however they are not the primary driver of genetic variability observed in gene-edited cell lines.

Our analysis of WGS data clearly demonstrated that the genetic variation between genome-edited cell lines is very small compared to inter-individual variation in classical hPSC-based disease models^87^, where patient-derived cell lines are compared to those from unrelated healthy individuals. However, since it is challenging to predict how the remaining variation could impact cellular phenotypes, it would be desirable that the control cell lines carry a comparable genetic variation. To evaluate how well the control lines (WIBR3_EWT_S1-8) represent the genetic variation within the iSCORE-PD collection, we computed the phylogenetic relationship of all gene edited cell lines (Fig. 5D). Consistent with the results described above, correctly targeted clones were most closely related to untargeted controls from the same experiment (e.g., *SNCA*-A30P and EWT_S1-3), suggesting that control cells derived from the same targeting experiment are best to represent the genetic variability of the edited clones. Importantly, this analysis revealed that WIBR3_EWT_S1-8 are genetically distributed across all gene-edited cell lines, indicating they cover the genetic variability of the entire iSCORE-PD collection. This phylogeny provides a systematic strategy to select the most appropriate controls for a given experiment based on the closest genetic correlation between controls and gene-edited cell lines.

## Discussion

Advances in population genetics and sequencing technologies have greatly enhanced our understanding of the genetic architecture of complex diseases, leading to the identification of numerous genetic variants linked to the development and progression of diseases, such as PD. However, revealing the functional role of these variants within a genetically diverse population remains a significant challenge. To overcome this limitation, we have generated a collection of isogenic hESC lines that carry monogenic or high-risk PD-associated mutations. Like the development of inbred animal models, which have proven instrumental in establishing robust genotype-phenotype correlations and have enabled the comparison of phenotypes across research groups, our isogenic cell line collection offers the opportunity to directly compare the phenotypic effect of PD-associated mutations in a genetically controlled system across genes and laboratories.

The establishment of an isogenic cell line collection involves two components, both crucial for the effective implementation of this approach: (1) a thorough characterization of the parental hPSC line and (2) development of a genome editing platform enabling the efficient engineering of genetic alterations similar to those found in patients. Regarding the hPSC line, we have conducted a comprehensive analysis of the parental WIBR3 hESC line and demonstrated that its genomic integrity can be sustained over extended periods in culture. We also show that WIBR3 cells are amenable to multiple rounds of clonal expansion and genome engineering. In addition, high-density genotyping and long-read sequencing show that the WIBR3 cell line does not carry major structural or genetic alterations impacting genes with known relevance to PD. Importantly, we demonstrate that WIBR3 cells can efficiently generate PD-relevant cell types in vitro using established differentiation protocols. Thus, WIBR3 cells are a highly characterized female hESC line, providing a valuable complement to existing hPSC lines for modeling neurodegenerative diseases.

To generate this collection, we established CRISPR/Cas9, TALEN, and prime editing pipelines in hPSCs, enabling the highly efficient and multiplexed introduction of a broad range of disease-associated genetic alterations, ranging from heterozygous and homozygous SNVs to large structural genomic deletions. During the process of establishing this collection, we made several key observations. Notably, we recognized that all genome editing approaches necessitate a comprehensive QC process beyond the validation of the intended genomic modification. Consequently, all the hESC lines described underwent a rigorous QC procedure, which included the validation of pluripotency and the exclusion of karyotypic and structural aberrations using standard aCGH arrays, high-density genotyping arrays, and zygosity analysis at the targeted genomic locus.

To further test the concept that genome editing can be used to generate isogenic cells that differ exclusively at the intended editing site, we used WGS to comprehensively assess the genetic variation within the iSCORE-PD collection. Our analysis demonstrated that the genetic variation between genome-edited cell lines is neglectable compared to inter-individual differences observed in classical hPSC-based disease models^87^, where patient-derived cell lines are compared to those from unrelated healthy individuals. However, the potential impact of specific variants on cellular and disease phenotypes remains unpredictable even in isogenic experiments and can pose significant problems when comparing just a single pair of genome-edited cell lines.

Our analysis revealed two main findings. First, perfect isogenic cell lines do not exist in in vitro cellular systems due to genetic variation introduced by both cell culture and genome editing. Second, the vast majority of this genetic variation in genome-edited hPSCs arises from preexisting variants in the parental founder cell line acquired during routine cell culture, which become fixed through a founder effect during the clonal expansion process of genome editing (Fig. 4A). The observation that many of the shared variants in genome edited cell lines are already present in the parental cell population has three important implications for the use of genome edited cell lines in disease modeling: (1) the best approach to control for this genetic variation is to include multiple independently targeted disease-associated cell lines and controls. (2) Untargeted, clonally derived cells from the same targeting experiment are the best controls, as they most accurately represent the genetic variability of the edited clones. (3) Multi-step cloning strategies carry the highest risk of generating lines with shared variants, as every consecutive editing step propagates the genetic variation present from the preceding manipulation. Together, these findings emphasize the importance of carefully designing genome-editing experiments to account for and mitigate the effects of shared genetic variants on downstream phenotypic analyses.

Finally, by analyzing mutations that are unique in each cell clone, we estimate that with each passage, hPSCs acquire about 20 additional mutations. Previously, it has been suggested that one way to distinguish mutation-specific phenotypes from alterations coming from unintended genetic variation is to revert the genome-edited cell lines to the wild-type genotype^88^ in a second step. This strategy is highly useful to validate specific mutation-associated phenotypes. However, our analysis suggests that relying on a single cell clone to identify  novel or subtle phenotypes may be insufficient to account for the genetic variation that is introduced de novo by the continues culturing of cells. Moreover, the mutation correction approach bears complications when comparing phenotypes across different disease-causing mutations, as each carries numerous cell line-specific mutations.

In addition, we address the outstanding question whether CRISPR/Cas9 or prime editing is better suited for generating genome-edited hPSC collections. A key finding across the derivation of all cell lines in the iSCORE-PD collection was that the frequency of karyotypic and structural aberrations, as well as LOH of the edited locus, was more frequent in CRISPR/Cas9 than in prime edited cell lines. Moreover, we only detect off-targets in cell lines generated using CRISPR/Cas9. This is the first time that this has been formally reported across a large cohort of gene edits combined with a detailed genotyping approach. This finding is consistent with the known mechanism of CRISPR/Cas9-based genome editing, which introduces a potentially genotoxic double-strand break (DSB) at the target site to facilitate the insertion of genetic modifications that is frequently processed through complex DNA repair reactions. Instead, prime editing only introduces single-strand DNA nicks, a genetic insult that is more readily repaired by a cell without mutations or genomic rearrangements, driving the repair outcome toward the intended genetic modification^89^.

Furthermore, our analysis confirmed that off-target effects, though rare, can occur in genome-edited cell lines. While the number of genuine off-target events was low even in CRISPR/Cas9 edited clones (4 in 41 analyzed cell lines), it is important to note that no off-target effect was detected in prime edited cell lines. Together, these results confirm our previous observations that prime editing has substantial advantages over CRISPR/Cas9-based approaches for introducing point mutations and small structural modifications in hPSCs^74^. Furthermore, we strongly recommend including a zygosity analysis, specifically to exclude LOH at the target locus, as a critical step in the QC pipeline of genome-engineered hPSCs.

Given that any genetic alteration induced by cell culture or genome editing can impact the biological properties of hPSCs and disease phenotypes, our in-depth analysis of the genomic integrity and variability of the iSCORE-PD collection underscores the need for a comprehensive QC in genome engineering^90^ and disease modeling. An additional, well-established concern in female hPSC lines is the phenomenon of X-chromosome erosion, which can emerge over time in culture and lead to aberrant expression of X-linked genes^91–93^, thereby altering cellular phenotypes and confounding the interpretation of experimental outcomes. Monitoring X-inactivation status, therefore, is essential to ensure reproducibility and accurate data interpretation. Because clonally derived cell lines remain susceptible to cell culture-induced genetic drift as well as genetic and epigenetic alterations, including X-chromosome erosion, we advocate using multiple independently gene-edited clonal lines for each genotype to account for this variability. In addition, we recommend using low-passage-number cell lines and performing routine QC analysis, including WGS, to detect culture-induced genetic aberrations. This approach ensures a robust assessment of disease-relevant phenotypes in vitro, acknowledging the potential variability that may arise during prolonged cell culture and genome-editing processes.

While perfect isogeneity remains elusive, the observed genetic variations were minimal compared to those typically found in classical hiPSC experiments comparing cells from patients with those from unaffected individuals. Predicting the impact of this variation on cellular and disease phenotypes is challenging. However, as the majority of these variations are random and predominantly affect noncoding regions of the genome, similar to somatic mutations found ubiquitously across normal tissues, we predict that most of the observed variation is unlikely to strongly impact cellular fitness or disease-associated phenotypes^81^. In parallel work, we have already examined global gene expression and pre-mRNA splicing patterns in in vitro-differentiated, midbrain-specific dopaminergic neurons carrying pathogenic PD mutations in *PRKN, SNCA, LRRK2, PINK1, DNAJC6, FBXO7, SYNJ1, DJ1/PARK7, VPS13C, ATP13A2*, and *GBA1*. This analysis revealed mutation-associated alterations in pre-mRNA splicing that overlap with changes observed in postmortem brain tissue from PD patients^94^. In addition, we used in vitro differentiated midbrain-specific dopaminergic neurons carrying the *LRRK2* G2019S mutation to cooperate findings from PD patient brain samples showing that *TDP-43* LOF drives aberrant splicing in PD^95^. Therefore, we believe that such variants do not compromise the value of genetically controlled hPSC collections like iSCORE-PD in disease research. Importantly, we provide a roadmap for effectively managing these variations through stringent QC measures and careful experimental design.

The cell lines described here currently focus on coding risk variants with a large effect size linked to monogenic PD^4^. We envision that we and other researchers will continue to expand this collection to include additional cell lines with diverse genetic backgrounds and eventually incorporate GWAS-identified risk variants with a lower effect size. Such an expansion could provide functional insights into how these primarily noncoding sequence variants affect similar cellular and molecular pathways as implicated in monogenic PD. To facilitate such efforts, all generated cell lines will be made available with the support of the ASAP initiative and the Michael J. Fox Foundation (MJFF) through the WiCell Research Institute. We anticipate that the subsequent biological analysis of this comprehensive collection and its future expanded forms, involving numerous research groups with diverse expertise, can provide a unified understanding of how genetic risk variants functionally contribute to the pathogenesis of PD. We predict that this collaborative effort has the potential to accelerate the development of novel therapeutic strategies for PD.

## Methods

All research involving hESCs and induced pluripotent stem cells (hiPSCs) or human tissue was conducted in accordance with applicable U.S. federal regulations and institutional guidelines. The study protocol was reviewed and approved by the Institutional Review Board (IRB) and the Embryonic Stem Cell Research Oversight (ESCRO) Committee at Albert Einstein College of Medicine and the University of California, Berkeley. All procedures involving human materials complied with 45 CFR 46 and the NIH Guidelines for Human Stem Cell Research and follow the principles laid out in the 2021 ISSCR Guidelines for Stem Cell Research and Clinical Translation (https://www.isscr.org/guidelines).

### hPSCs culture

hESCs were maintained on irradiated or mitomycin C-inactivated mouse embryonic fibroblast (MEF) monolayers as described previously^74^ with daily changes of hESC media (Dulbecco’s Modified Eagle Medium/Nutrient Mixture F-12 (DMEM/F12; Thermo Fisher Scientific) supplemented with 15% fetal bovine serum (Hyclone), 5% KnockOut Serum Replacement (Thermo Fisher Scientific), 1 mM glutamine (Invitrogen), 1% nonessential amino acids (Thermo Fisher Scientific), 0.1 mM β-mercaptoethanol (Sigma) and 4 ng/ml fibroblast growth factor (FGF) (Thermo Fisher Scientific/Peprotech), 1×Penicillin-Streptomycin (Thermo Fisher Scientific). All hESCs cultures were maintained in a cell culture incubator under low oxygen conditions (5% CO_2_, 5% O_2_). Cultures were passaged as aggregates every 5–7 days using a collagenase IV solution (Gibco) to detach hESC colonies. All cell lines were tested routinely for mycoplasma. Detailed protocols for culturing of hESCs on MEF feeders can be found on protocols.io (10.17504/protocols.io.b4pbqvin; 10.17504/protocols.io.b4msqu6e). All hESCs cultures were adapted to feeder-free culture conditions before starting in vitro differentiation experiments. hESCs were maintained on Geltrex/Matrigel-coated plates in mTeSR plus medium (Stem Cell Technologies) in a cell culture incubator under low oxygen conditions (5% CO_2_, 5% O_2_) as described previously^74^. Cells were passaged regularly as aggregates either manually or using ReLeSR (Stem Cell Technologies) to detach hESC colonies. Detailed protocols for feeder-free culturing of hPSCs can be found on protocols.io (10.17504/protocols.io.b4mcqu2w).

### Collecting cell pellets for DNA and RNA extraction

hESCs colonies cultured on MEFs were harvested by collagenase IV and washed twice through an 80 µm cell strainer to further remove MEFs. Collected colonies were pelleted by centrifugation and snap frozen in liquid nitrogen.

### Array genotyping and data processing

Genomic DNA was isolated from cell pellets using the DNeasy Blood & Tissue Kit (QIAGEN; 69504). Genotyping was performed using the NBA with best practices guidelines for the Infinium GDA^46^. Genotyping data was processed using GenomeStudio (RRID:SCR_010973), and subsequent genotype calls, B-allele frequency, and LogR ratio values were used for genomic integrity assessments. When cell lines carrying a genomic edit were present on the NBA, genotype calls were compared to confirm the edit. Genome-wide genotyping calls were compared with the PacBio HiFi WGS variants to assess large genomic events across the two data types using PLINK (v1.9, RRID:SCR_001757)^96^. The B-allele frequency and LogR ratio values were processed and plotted using the GWASTools package (https://bioconductor.org/packages/release/bioc/html/GWASTools.html) in R (RRID:SCR_001905; v3.6.1, https://www.r-project.org/, 10.18129/B9.bioc.GWASTools)^97^.

### Long-read sequencing and data processing

#### Oxford Nanopore Technologies DNA extraction, library preparation, and sequencing

Ultra-high molecular weight DNA (UHMW) was extracted from the WIBR3 (parental) hESC line (5 × 10^6^ cells) following the Circulomics/Pacific Biosciences (PacBio) UHMW DNA Nanobind Extraction protocol (Circulomics/PacBio, no longer available) with the Nanobind CBB Kit (PacBio, SKU 102-301-900) and the UHMW DNA Aux Kit (Circulomics/PacBio, NB-900-101-01, no longer available). The extracted DNA was checked using the Qubit dsDNA BR assay (Invitrogen, Q32850) to ensure proper extraction occurred. The extracted UHMW DNA was then taken straight into library preparation for sequencing using Oxford Nanopore Technologies (ONT) SQK-ULK001 Kit and the Nanobind Ultra Long Library Preparation Kit (Circulomics/PacBio, NB-900-601-01, no longer available). The library was split into 3 tubes of 75 µl each, and each tube was loaded on a flow cell. After 24 h, 75 µl of the sequencing library was pulled out of each flow cell and reloaded on a fresh flow cell. This process was repeated one more time for a total of 9 separate R9.4.1 PromethION flow cells.

#### Pacific Biosciences DNA extraction, library preparation, and sequencing

High molecular weight (HMW) was extracted using PacBio’s Nanobind CBB Kit (Pacbio, 102-301-900) from 2 × 10^6^ cells with the Nanobind adherent cultured cells protocol. After extraction, DNA concentration was quantified using the Qubit dsDNA BR assay (Invitrogen, Q32850), sized with a Femto Pulse System (Agilent, M5330AA), and size-selected with the PacBio SRE Kit (Pacbio, SKU 102-208-300). Following quality control, the extracted DNA was sheared to a target size of 18–20 kb using the Megaruptor 3 (Diagenode, B060100003). After confirmation of correct sizing, the library preparation was performed the SMRTbell prep kit 3.0 (PacBio, 102-141-700) with a PEG wash. The library was sequenced on a Revio flow cell with a 24 h movie time.

#### Long-read sequencing data analysis

ONT sequencing runs were basecalled on NIH’s HPC (Biowulf) using Oxford Nanopore’s Guppy (v6.1.2, RRID:SCR_022353) in super accuracy mode with the *dna_r9.4.1_450bps_modbases_5mc_cg_sup_prom.cfg* configuration file and the –bam_out option to preserve methylation tags. The basecalled bams were then converted to fastqs using Samtools (v1.17, RRID:SCR_002105)^98^ (samtools fastq -TMm, Ml) and mapped to hg38 using Minimap2 (v2.24, RRID:SCR_018550)^99^ with ONT flags. Data from all flow cells was merged after mapping using samtools (v1.17, RRID:SCR_002105). Then, we used PEPPER-Margin-DeepVariant (v.0.8, https://github.com/kishwarshafin/pepper)^100^ to call small variants (<50 bp) and phase our variant calls and alignments. We then used our phased alignment to produce haplotype-specific methylation calls using Modbamtools (v0.4.8, https://rrazaghi.github.io/modbamtools/)^101^ and Nanopore’s modbam2bed (https://github.com/epi2me-labs/modbam2bed). Lastly, structural variants (SVs) were called using Sniffles2 (v2.2, RRID:SCR_017619)^102^ with default settings. PacBio Revio HiFi data was processed according to general best practices. Data was mapped using Minimap2 (v2.24, RRID:SCR_018550) using PacBio flags. Small variant calls generated by Clair3 (RRID:SCR_026063; v1.0.4) (https://github.com/HKU-BAL/Clair3) with PacBio flags and SV calls were generated by Sniffles2 (v2.2, RRID:SCR_017619)^102^. Small variants were filtered for DP > 15 and GQ > 20 using bcftools (v1.17, RRID:SCR_005227)^98^ and annotated with ANNOVAR (v.2022-06-08, RRID:SCR_012821)^103^ to assess the presence of potential pathogenic variants, specifically, based on variant information (GRCh38), gene-based annotations (refGene), variant identifiers (dbSNP build 150)^104^, deleteriousness and pathogenicity prediction scores (dbNSFP)^105^, genome and exome population allele frequencies (gnomAD v4.1; https://gnomad.broadinstitute.org/news/2024-04-gnomad-v4-1/)^55^, pathogenicity annotations (ClinVar, RRID:SCR_006169; accessed January 12, 2024)^106^, and dosage sensitivity annotations (ClinGen; RRID:SCR_014968; https://clinicalgenome.org/; accessed September 10, 2025)^107,108^. To assess their potential role in neurodegeneration, these variants were further annotated with known functional categories in neurodegeneration from the KEGG^109^ and HPO gene signature collections (https://hpo.jax.org/)^110^ (i.e., KEGG Alzheimer’s and Parkinson’s disease, and HPO AD, Parkinsonism, and neurodegeneration) from the MSigDB (RRID:SCR_016863; v2025.1) database (https://www.gsea-msigdb.org/gsea/msigdb/index.jsp)^111–113^. In addition, AD^114^ and Parkinson’s disease genetic^50^ risk scores (excluding UK Biobank summary statistics) were calculated to assess the cumulative risk score using plink (v2.0, RRID:SCR_001757) for disease and compared with participants from the UK Biobank diagnosed with AD and PD^115^ (https://www.ukbiobank.ac.uk/ via controlled access; application ID: 33601). Only SV calls labeled as “PASS” were kept for both ONT and PacBio data. The “PASS” SV calls were then annotated with ANNOVAR (v.2022-06-08, RRID:SCR_012821)^103^, and coding variants were subset. Then, we used Truvari (v4.4.0)^116^ (https://github.com/ACEnglish/truvari) to merge structural variant calls between the ONT and PacBio datasets, both for all variants as well as only coding variants. Numbers on variant type distribution were generated using SURVIVOR (v1.0.7, RRID:SCR_022995). Lastly, the SV overlaps were further annotated using SVAnna^53^ (v1.0.4) (https://github.com/TheJacksonLaboratory/SvAnna with the phenotype terms HP:0002180 (neurodegeneration) and HP:0000707 (abnormality of the nervous system). SVs of interest were plotted using samplot (v1.3.0) (https://github.com/ryanlayer/samplot).

#### WGS analysis pipeline

DNA samples were sequenced with Illumina short-read WGS at Psomagen (Rockville, MD), with a mean coverage of 30x. Data were processed using standard GP2 WGS pipelines. In brief, 150 bp paired-end reads were aligned to the human reference genome (GRCh38 build) using BWA-mem (RRID:SCR_022192; r1273; https://github.com/lh3/bwa) following the functional equivalence pipeline^117^. Sample processing and variant calling were performed using DeepVariant v.1.6.1^83^. Joint-genotyping was performed using GLnexus v1.4.3 with the preset DeepVariant WGS configuration^84^. A detailed description of the WGS pipeline can be found at https://github.com/GP2code/releases/tree/main/BETA-APR2022/wgs_var_calling and https://github.com/GP2code/GP2-WorkingGroups/tree/main/MN-DAWG-Monogenic-Data-Analysis/Terra_wdl/variant_calling/deepvariant.

### Analysis pipeline to identify clone-specific SNVs/indels

To identify SNVs/indels for each group of cell lines modified by a set of CRISPR/Cas9 or prime editing reagents, the variants identified with the WGS pipeline were first filtered to only consider calls with a GLNexus quality score greater or equal to 30. Then, to identify SNVs/indels specific to only one edited group, the SNVs were further filtered to remove any calls found in any of the other cell lines in the collection. The remaining variants are what account for each editing group’s specific SNVs/indels and were used to determine unique and shared variants for each clonal cell line within each edited group. These unique and shared SNVs/indels were used to further characterize their contribution to coding or noncoding regions as well as their effect on the coding sequences by leveraging tools within the BioConductor DeepVariant package^118^ against the TxDb.Hsapiens.UCSC.hg38.knownGene_3.18.0 human transcript annotation package (based on the UCSC hg38 genome, based on the knownGene table). A detailed description of the pipeline can be found here (https://github.com/hockemeyer-ucb/pd-sv-analysis). The group's unique SNVs/indels were used to generate a phylogeny tree using the BioConductor package fastreeR^119^ and visualized using the R package ape (RRID:SCR_017343; 5.8-1; https://CRAN.R-project.org/package=ape)^120^.

### Off-target analysis

To test whether some of the group-specific SNVs could be linked to potential off-target effects triggered by the CRISPR/Cas9 or prime editing reagents, all putative off-target sites were identified using Cas-OFFinder^86^ (RRID:SCR_023390; v2.4.1; https://github.com/snugel/cas-offinder) and used to identify nearby SNVs/indels that could have been the result of the editing strategies. A detailed description of the pipeline can be found here (https://github.com/hockemeyer-ucb/pd-sv-analysis).

### Molecular cloning

Molecular cloning was carried out as described previously^74^ following standard cloning protocols (https://www.cshlpress.com/pdf/sample/2013/MC4/MC4FM.pdf)^121^. As described^89^, pegRNA plasmids for prime editing were cloned by ligating annealed oligonucleotide pairs (Supplementary Data 9) into the BsaI-digested pU6-peg-GG-acceptor (pU6-pegRNA-GG-acceptor was a gift from David Liu. RRID:Addgene_132777; http://n2t.net/addgene: 132777; RRID:Addgene_132777). Prime editing nicking guide plasmids (ngRNAs) were cloned by ligating annealed oligonucleotide pairs (Supplementary Data 9) into the BsmBI-digested pBPK1520 plasmid (BPK1520 was a gift from Keith Joung. Addgene#65777; http://n2t.net/addgene: 65777; RRID:Addgene_65777)^122^. For CRISPR/Cas9-based genome editing, the Cas9-expressing sgRNA plasmids were cloned by ligating annealed oligonucleotide pairs (Supplementary Data 9) into the BbsI-digested px330-GFP (RRID:Addgene_97084)^40^ or px330-mCherry (RRID:Addgene_98750) as described previously^40^. For TALEN-mediated genome editing, we used previously described heterodimeric TALEN pairs to insert the G2019S into the *LRRK2* gene^74^. Sequence information for all oligonucleotides (Integrated DNA Technologies, IDT) used to generate plasmids can be found in Supplementary Data 9.

### Genome editing of hESCs

As outlined in Fig. 3C, genome editing of WIBR3 hESCs was performed using either plasmid or ribonucleoprotein particle (RNP) based CRISPR/Cas9 or prime editing approaches as described previously^74^ using the following procedures:

### Nucleofection

hESCs cultured on MEFs were pre-treated with 10 µM ROCK inhibitor (Y27632, ToCris) 1-day before nucleofection (2–3 h at a minimum is recommended). Cells were collected by collagenase IV (Thermo Fisher Scientific), followed by Accutase (Thermo Fisher Scientific) to dissociate hESCs into a single cell solution. 5 × 10^5^ to 1 × 10^6^ cells were resuspended in 20 µL of nucleofection solution (P3 Primary Cell 4D-Nucleofector™; Lonza) and nucleofected (Lonza 4D nucleofector TM Core + X Unit, program CA-137) using the following genome editing reagents for the corresponding edits described in Fig. 3: (1) plasmid-based CRISPR-Cas9 facilitated HDR: 200 ng sgRNA plasmids (px330-GFP), 700 ng ssODN. (2) Plasmid based dual CRISPR: 500 ng 3′-sgRNA plasmid (px330-GFP) and 500 ng 5′-sgRNA plasmid (px330-mCherry (RRID:Addgene_98750)). (3) TALEN facilitated HDR: 100 ng LRRK2-TALEN-TA01L and 100 ng LRRK2-TALEN-TA03R, 700 ng ssODN, 100 ng pEGFP-N1 (RRID:Clontech_6085-1). (4) Plasmid based prime editing: 500 ng pCMV-PE2-GFP (a gift from David Liu, RRID:Addgene_132776)^89^, 330 ng pU6-pegRNA (RRID:Addgene_132777) and 170 ng pBPK1520-ngRNA (RRID:Addgene_65777). (5) RNP-based CRISPR-Cas9 facilitated HDR: 80 pmol purified Cas9 protein (QB3 Macrolab, UC Berkely), 300 pmol chemically modified synthetic sgRNA (Synthego), and 100 pmol ssODN HDR template. (6) RNP-based dual CRISPR: 80 pmol purified Cas9 protein, 150 pmol of each chemically modified synthetic 3′-sgRNA and 5′-sgRNA. (7) RNP-based CRISPR-Cas9 facilitated HDR with competing templates: 80 pmol purified Cas9 protein, 300 pmol chemically modified synthetic sgRNAs, 50 pmol ssODN HDR template carrying PD mutation, and 50 pmol ssODN HDR template carrying a synonymous mutation. (8) RNA-based prime editing: 4 μg in vitro transcribed nCas9-RT mRNA, 100 pmol chemically modified synthetic pegRNA (IDT or Synthego) and 50 pmol chemically modified synthetic ngRNA (Synthego). Detailed protocols can be found on protocols.io (10.17504/protocols.io.e6nvwkkewvmk/v2).

### Editing pipelines

We used two different editing pipelines, termed *pipeline A* and *pipeline B,* to generate the iSCORE-PD collection (Fig. 3A, B). The editing pipeline used to create each cell line in the iSCORE-PD collection is included in Supplementary Data 3.

*Pipeline A* utilizes FACS-enrichment post-nucleofection to purify effectively transfected cells, followed by clonal expansion and genotyping to allow the isolation of clonal, correctly targeted lines (estimated time for the editing pipeline is 30–35 days). Following nucleofection, the hESCs are plated on MEFs in 10 µM ROCK inhibitor (Y27632, ToCris) containing hESC media (previously described in hPSC culture section) at high density (1 nucleofection/1 well 6-well plate). Forty-eight to seventy two hours after nucleofection, Accutase-dissociated single cells are FACS-sorted for the expression of the respective fluorescent marker protein and either directly used for bulk NGS-based validation of the desired genome modification or subsequently plated at clonal density (250–350 cells/cm^2^) on MEFs in hESC media supplemented with 10 µm ROCK inhibitor (Y27632, ToCris) for the first 24 h. Individual colonies are picked and grown 7–14 days after electroporation. Correctly targeted clones were subsequently identified by Sanger or NGS sequencing. A detailed protocol can be found on protocols.io (10.17504/protocols.io.b4piqvke).

*Pipeline B* (high-throughput hPSCs genome editing) involves low cell number nucleofection, limited dilution, and NGS-dependent genotyping to identify desirable edits in a 96-well plate system. This integrated workflow allows the efficient isolation of correctly edited clonal cell lines, even at low frequency, within a shorter time frame compared to previous approaches (21–35 days). As described previously^74^, the nucleofected cells are directly seeded onto MEFs in 96-well plates, at seeding densities of 1000 cells/plate in hPSCs media containing 10 µm ROCK inhibitor (Y27632, ToCris). After individual colonies appear around day 14, plates are duplicated for (1) maintenance and (2) DNA extraction for NGS-based identification of wells that contain cells with the desired genetic modification. To duplicate plates, cells are washed with PBS (Corning) and treated with 40 µL 0.25% trypsin for 5 min at 37 °C. Sixty microliters hESC media containing 10 µM Rock inhibitor (Y27632, ToCris) is added to each well to inactivate trypsin. Cells are gently dissociated, and half (50 µL) of the cell suspension is reseeded to a new MEF containing a 96-well plate pre-loaded with 100 µL hPSC media containing 10 µM Rock inhibitor (Y27632, ToCris) and cultured for another 7 days with hPSC media.

### NGS-based identification of validation of targeted clonal lines

Fifty microliters of cell suspension/well obtained during plate duplication is transferred to a 96-well PCR plate pre-loaded with 50 µL 2× lysis buffer (100 mM KCl, 4 mM MgCl_2_, 0.9% NP-40, 0.9% Tween-20, 500 µg/mL proteinase K, in 20 mM Tris-HCl, pH 8) for DNA extraction (50 °C overnight incubation followed by 95 °C 10 min [proteinase K inactivation]). A ~300 bp genomic region covering the designed mutation is amplified (Supplementary Data 9), containing NGS barcode attachment sites (GCTCTTCCGATCT) from 2 µl cell lysis from each well with Titan DNA polymerase. Amplicons were purified at the UC Berkeley DNA Sequencing Facility, then i5/i7 barcoded in indexing PCR, pooled, and sequenced on 150PE iSeq in the NGS core facility at the Innovative Genomics Institute. CRISPResso2 (RRID:SCR_021538)^123^ in prime editing mode was used to analyze the NGS data to identify wells containing the designed mutation, with the following criteria. Heterozygous candidates: number of reads aligned >100, 70% >mutant allele frequency >20%, indels frequency <5%; homozygous candidates: number of reads aligned >100, mutant allele frequency >70%, indels frequency <5%. Wells containing the desired editCells in those identified wells were single-cell subcloned once and genotyped clonally to confirm cell line purity to ensure clonality. Detailed protocols for high-throughput hPSCs genome editing (10.17504/protocols.io.b4mmqu46) and genotyping by NGS 10.17504/protocols.io.b4n3qvgn) can be found on protocols.io. For clarity, NGS results reported in any of the figures of this publication showcase only representative reads. Any NGS reads below 1% of the total result were removed. The full NGS report can be found with the rest of the raw data files (ASAP CRN Cloud: team-rio-hesc-targeted-ngs-mutant-zygosity; 10.5281/zenodo.19600812).

### Zygosity confirmation by SNV detection

The SNV closest to the editing site for each genetic edit was identified from the WGS data of parental WIBR3 hESCs. A genome DNA region flanking the SNV and the editing site was amplified by PCR (Supplementary Data 9) and sequenced by Sanger sequencing or NGS. Clones showing LOH were removed from the final collection.

### Cortical spheroid differentiation

hESCs were differentiated into early cortical spheroids following an adaptation of a published protocol (10.1016/j.tcb.2019.11.004, 10.1038/nmeth.3415)^124,125^. In brief, hESC colonies were dissociated and plated into pre-coated 6-well Aggrewell 800 plates at a concentration of 18 M cells per well in hESC media with 10 µM Rock Inhibitor (ToCris). The next day (day 1), the aggregates were removed, sedimented, and added to an ultralow adherence plate with hESC media supplemented with 5 µM Dorsomorphin (SelleckChem) and 10 µM SB431542 (SelleckChem) (media changed daily). On day 6, media was replaced with Neural Precursor Expansion Media (Neurobasal medium + B27 supplement without vitamin A (2% vol/vol) + Penicillin-Streptomycin (100 U/ml) + GlutaMAX (1% vol/vol) + HEPES buffer (1% vol/vol) + FGF2 (20 ng/ml) + EGF (20 ng/ml)) (media is changed every day until day 16 and then every other day until day 25). For specific details, consult published materials on protocols.io: 10.17504/protocols.io.5jyl8po57g2w/v1.

### Cell irradiation

hESCs were transferred from MEFs to a feeder-free Matrigel substrate with conditioned media for 2 weeks previous to this experiment. hESCs at 50% confluence or cortical spheroids on day 25 of differentiation were irradiated at 0, 0.5, 2, 5, and 10 Gy using a discrete cesium source. Twenty-four hours post-irradiation, cells were collected and dissociated for MULTI-Seq barcoding and sequencing. For specific details, consult published materials on protocols.io (10.17504/protocols.io.bp2l6xwbzlqe/v1).

### MULTI-Seq barcoding and single-cell library preparation of irradiated samples

*hESCs:* each irradiation condition was labeled with a lipid-modified barcoded MULTI-seq oligo following a previously described protocol^126^. In short, cells in PBS were incubated with a 1:1 molar ratio of lipid-modified Anchor Oligo:Barcode Oligo for 5 min on ice. Then an equimolar amount of lipid-modified co-anchor was added for an additional 5 min incubation on ice. Then, cells were washed twice with ice-cold PBS (Corning) + 1% BSA (Fisher) to sequester the anchor oligos, strained, counted, and pooled for single-cell sequencing. 10× single-cell RNA sequencing was performed according to the manufacturer’s instructions using the Chromium Single Cell 3′ Reagent Kits v3 with Feature Barcoding Technology. For specific details, consult published materials on protocols.io (10.17504/protocols.io.kxygx3xzkg8j/v1). Deconvolution of MULTI-Seq barcodes was performed as described previously^126^ using the MULTI-seq package at https://github.com/chris-mcginnis-ucsf/MULTI-seq/.

*Cortical spheroids:* single-cell suspensions were FACS-sorted to remove debris and aggregates, then 10× single-cell RNA sequencing was performed according to the manufacturer’s instructions using the Chromium Single Cell 3′ Reagent Kits v3, targeting 2000 cells per irradiation condition using one 10× lane per condition. Single-cell analysis of all irradiated samples was performed using Seurat v4 (RRID:SCR_016341)^127^ according to default parameters for normalization and integration of data sets. Droplets with more than 15% mitochondrial reads detected were excluded as poor analysis candidates due to the likelihood of cell death resulting in poor RNA representation. Plots were generated using ggplot2 (RRID:SCR_014601) in R.

### Dopaminergic neuron differentiation

Feeder-free adapted WIBR3 hESCs were differentiated into dopaminergic neurons as per previously reported protocols with slight modifications (10.1016/j.stem.2021.01.004, 10.1016/j.stem.2021.01.005)^66,67^. Briefly, hESC colonies were dissociated into single cells and seeded onto Matrigel-coated plates at a density of 400–600k cells per well of a 6-well plate in mTeSR (Stem Cell Technologies) containing 10 µM Rock inhibitor (Y27632, ToCris). Differentiation was induced sequentially with media A—3 days (Neurobasal media (Gibco) + N2 supplement (Gibco; 1% vol/vol) + B27 supplement without vitamin A (Gibco; 2% vol/vol) + L-glutamine (Gibco; 2 mM) + penicillin-streptomycin (Gibco; 100 U/ml) + SHH C25II (R&D systems; 100–200 ng/ml) + CHIR99021 (ToCris; 0.7 µM) + LDN (Stemgent; 250 nM) + SB431542 (SelleckChem; 10 µM)), B—3 days (Neurobasal media (Gibco) + N2 supplement (Gibco; 1% vol/vol) + B27 supplement without vitamin A (Gibco; 2% vol/vol) + L-glutamine (Gibco; 2 mM) + penicillin-streptomycin (Gibco; 100 U/ml) + SHH C25II (R&D Systems; 100–200 ng/ml) + CHIR99021 (ToCris; 7.5 µM) + LDN (Stemgent; 250 nM) + SB431542 (SelleckChem; 10 µM)), C—3 days (Neurobasal media (Gibco) + N2 supplement (Gibco; 1% vol/vol) + B27 supplement (Gibco; 2% vol/vol) + L-glutamine (Gibco; 2 mM) + penicillin-streptomycin (Gibco; 100 U/ml) + CHIR99021 (SelleckChem; 7.5 µM)) and D—1 day (Neurobasal media (Gibco) + B27 supplement (Gibco; 2% vol/vol) + L-glutamine (GIbco; 2 mM) + penicillin-streptomycin (Gibco; 100 U/ml) + BDNF (PeProtech; 20 ng/ml) + GDNF (PeProtech; 20 ng/ml) + ascorbic acid (Sigma; 200 µM) + dibutyryl-cAMP (SelleckChem; 0.5 mM) + TGFβ3 (R&D Systems; 1 ng/ml) + CHIR99021 (SelleckChem; 3 µM)) over an 10 day period. On day 11, cells were dissociated and plated (1:2 ratio) at high density and maintained in maturation media (Neurobasal media (Gibco) + B27 supplement (Gibco; 2% vol/vol) + L-glutamine (GIbco; 2 mM) + penicillin-streptomycin (Gibco; 100 U/ml) + BDNF (PeProtech; 20 ng/ml) + GDNF (PeProtech; 20 ng/ml) + ascorbic acid (Sigma; 200 µM) + dibutyryl-cAMP (SelleckChem; 0.5 mM) + TGFβ3 (R&D Systems; 1 ng/ml) + DAPT (ToCris; 10 µM)) until day 16, when they were replated at the similar high density in 12 well plate and left to mature until day 24. On day 25, cells were dissociated for the final time with Accutase and replated at no less than 1–2 × 10^6^ cells per well of a 12-well plate and left to mature until post-differentiation experiments were carried out. For specific details, consult published materials on protocols.io: 10.17504/protocols.io.3byl4q8yovo5/v1.

### scRNA-seq of dopaminergic neurons—10× genomics library preparation

Dopaminergic neurons were harvested with Accutase on days 35–37 of culture and subsequently labeled with 10× Genomics CellPlex reagents, following the manufacturer's recommendation (10× Genomics CG000391 Rev B). After labeling with Cell Multiplexing Oligos (CMOs), samples were pooled and taken for 10× Genomics library preparation, following manufacturer recommendations with target capture of 30,000 cells per 10× lane (Chromium Single Cell 3’ Reagent Kits v3.1, User Guide CG000388 Rev C).

### scRNASeq of dopaminergic neurons—data analysis

After NGS of 10× Genomics libraries (NovaSeq 6000), FASTQ files were processed with 10× Genomics CellRanger pipeline (v7.0.1, RRID:SCR_017344) to demultiplex and generate count matrices for each sample. Data for each sample were first filtered to remove low-quality cells (cells with fewer than 1500 genes detected, greater than 30,000 RNA counts, and greater than 10% mitochondrial reads were removed). Filtered datasets were each processed individually with Seurat v4 (RRID:SCR_016341)^127^, using the SCTransform function for normalization and variance stabilization. Integration of the SCTransformed data was performed to generate a combined dataset of 10,097 cells. Data used in the preparation of this article (FOUNDIN-PD comparisons) were obtained on [2023-08-17] from the Parkinson’s Progression Markers Initiative (PPMI) database (https://www.ppmi-info.org/access-data-specimens/download-data), RRID:SCR_006431. For up-to-date information on the study, visit http://www.ppmi-info.org.

### Microglia differentiation

To generate in vitro differentiated microglia cells (iMGs), we adapted a previously published protocol^73^. Undifferentiated feeder-free hESC colonies maintained in mTeSR (Stem Cell Technology) were seeded at low density into cell culture flasks coated with reduced growth factor matrigel (30 colonies/T75 flask (Fisher)) using manual passaging. In vitro differentiation was achieved by sequential culture of the cells in the following media: step 1 (mTeSR (Stem Cell Tech) + 80 ng/ml BMP4 (PeProtech)—4 days), step 2 (StemPro-34 SFM (Gibco) + 2 mM GlutaMAX (Gibco) + 80 ng/ml VEGF (PeProtech), 25 ng/ml FGF (PeProtech) + 100 ng/ml SCF (PeProtech)—3 days), step 3 (StemPro-34 SFM (Gibco) + 2 mM GlutaMAX + 50 ng/ml SCF (PeProtech) + 50 ng/ml IL-3 (PeProtech) + 5 ng/ml TPO (PeProtech) + 50 ng/ul M-CSF (PeProtech) + 50 ng/ul Flt3 (PeProtech)—9 days) and step 4 (StemPro-34 SFM (Gibco) + 2 mM GlutaMAX + 50 ng/ml M-CSF (PeProtech) + 50 ng/ml Ftl3 (PeProtech) + 25 ng/ml GM-CSF (PeProtech)—14 days). After ~28 days, microglia progenitors are ready to be isolated and plated on Primaria plates (Corning) for maturation (at least 2 weeks) in microglia maturation media (Neurobasal media (Gibco) + N2 Neuroplex (Gemini; 1× final concentration) + GEM21 Neuroplex (Gemini; 1× final concentration) + 20% AlbuMAX I (Gibco; 0.2% final concentration) + NaCl (Fisher; 5 M) (50 mM final concentration) + sodium pyruvate 100x (Gibco’ 1× final concentration) + glutaMAX 100x (Gibco; 1× final concentration) + penicillin-streptomycin (Gibco; 100 U/ml) + 50 ng/ml TGF-β1 (PeProtech) + 100 ng/ml IL-34 (PeProtech) + 12.5 ng/ml M-CSF (PeProtech)). Detailed protocols for microglial differentiation can be found on protocols.io (10.17504/protocols.io.4r3l22zbjl1y/v1). Microglia cells were evaluated by immunocytochemistry (10.17504/protocols.io.yxmvm3146l3p/v1) and FACS-based analysis (10.17504/protocols.io.81wgbxokqlpk/v1) in order to confirm expression of precursor and mature microglia markers (CD16, CD45, CX3CR1, P2RY12, CD11b, CD14, IBA1, and PU.1). FACS data were analyzed using FlowJo software (RRID:SCR_008520; version 10.8.0; https://www.flowjo.com/solutions/flowjo). Abundance of the desired population was determined from the original sample by gating single cells and using calcein violet as a marker for viable cells. Gating strategy: SSC-A/FSC-A (cells minus debris) --> SSC-A/SSC-W (single cells) --> FSC-A/FSC-W (single cells) --> SSC-A/UV446-A(calcein violet positive population) --> PE/APC. All calculations derived of FACS analysis were properly corrected with negative and single color controls. Plots were generated by GraphPad (*N* = 4; MEAN ± SD).

### Immunocytochemistry

Immunocytochemistry was used to assess biomarker expression to characterize each of the cell types shown in this publication. Briefly, samples were fixed in PFA and permeabilized (0.03% triton when necessary) and blocked (BSA or serum) as required depending on the biomarker being analyzed on hESCs (OCT4 (DSHB: PCRP-POU5F1-1D2), SSEA4 (SSEA4 (DSHB: MC-813-70 (SSEA-4))) or our differentiated cell cultures: dopaminergic neurons (TH (Pel-Freez Biologicals: p60101-150), FOXA2 (R&D Systems: AF2400)) or microglia (IBA1 (Abcam), P2RY12 (Sigma), CX3XR1 (Biolegend), PU.1 (Cell Signaling Tech)). Fluorochrome-conjugated secondary antibodies were used to image our samples in an epifluorescence or confocal microscope. Alkaline phosphatase activity was measured using Vector® Black Substrate Kit, Alkaline Phosphatase (Vector Laboratories). Specific details on the protocol used can be found in protocols.io (10.17504/protocols.io.yxmvm3146l3p/v1). For specific details on our staining of pluripotency markers in our hESCs, consult: 10.17504/protocols.io.b4yyqxxw. OCT4, SSEA4, and AP Images from our hESC cultures were captured using a 10X objective on a fluorescence microscope (Zeiss ZEN 3.8). Magnification may differ depending on which microscope-camera set was used to capture the images. This was a result of which team within the collaboration was in charge of generating a specific cell line and its analysis through the QC steps.

### RNA isolation and qRT-PCR

Total RNA was isolated from cell pellets with the RNeasy kit (Qiagen). One to two micrograms of RNA was used for cDNA synthesis using the High-Capacity Reverse Transcriptase Kit (ThermoFisher Scientific). Real-time qRT-PCR was performed on the QuantStudio 6 Flex thermocycler using PowerUp SYBR green master mix (ThermoFisher Scientific). All reactions were prepared according to the manufacturer's instructions. Results were normalized to GAPDH and compared against human fibroblast samples (MRC-9, BJ1-hTERT, and GM01660). All primer sequences used for qRT-PCR were listed in Supplementary Data 9. For a detailed protocol consult: 10.17504/protocols.io.4r3l22r9pl1y/v1. Plots were generated using GraphPad Prism (RRID:SCR_002798, version 10.6.1 [324]).

### Southern blot

Southern blotting was performed following standard protocols (https://cshprotocols.cshlp.org/content/2021/7/pdb.top100396#cited-by) to validate the structural integrity and exclude the LOH at a genomic locus of interest (*PRKN*, *DJ1/PARK7*, *FBXO7,* and *SYNJ1*) resulting from CRISPR/Cas9 or prime editing-based genome editing experiments in hESCs. Southern blot probes were generated by PCR amplification (AccuPrime™ Taq DNA Polymerase, high fidelity (ThermoFisher)) of a 150 bp to 600 bp large genomic region 3′- and 5′ to the targeted genomic region. Southern blot probes were radiolabeled using the Prime-it Random Primer Labeling Kit (Agilent) according to the manufacturer’s instructions. Restriction-digested genomic DNA isolated from clonally expanded genome-edited hESC lines was separated on a 0.8% agarose (Sigma) gel, transferred to a nylon membrane (Amersham), and hybridized with 32P random primers labeled Southern blot probes. Oligonucleotide sequences and restriction enzyme information can be found in Supplementary Data 9. Detailed protocols for Southern blot analysis can be found on protocols.io (10.17504/protocols.io.bp2l6xe6dlqe/v1).

### Material and cell line availability

All cell lines in this isogenic collection will be made available to academic and/or nonprofit research institutions or researchers for research purposes, through the WiCell Research Institute (https://www.wicell.org/product-category/collection/mjff-iscore-collection/) supported by the ASAP initiative and the MJFF. Material transfer agreements will be required and administered centrally by WiCell for such academic and noncommercial requests. Requests for commercial use of these cell lines must be negotiated directly with the individual provider(s) of the relevant cell line(s). All newly generated plasmids are made available on Addgene (see Supplementary Data 9).

### Statistics and reproducibility

Bar graphs and XY plots were generated using GraphPad Prism 9 (RRID:SCR_002798, version 10.6.1 [324]). The number of biological and technical replicates, the statistical analysis performed, and the error bars are described in the respective figure legends. Representative phase-contrast and immunostaining images for pluripotency, differentiation of dopaminergic neurons, and microglial differentiation were obtained from a single experiment; however, pluripotency and differentiation were continuously monitored by light microscopy, and these findings were independently validated using complementary analyses, including flow cytometry (FACS) and qRT-PCR, performed in at least three independent cultures.

### Reporting summary

Further information on research design is available in the Nature Portfolio Reporting Summary linked to this article.

## Supplementary information

Supplementary Information

Description of Additional Supplementary Files

Supplementary Data 1

Supplementary Data 2

Supplementary Data 3

Supplementary Data 4

Supplementary Data 5

Supplementary Data 6

Supplementary Data 7

Supplementary Data 8

Supplementary Data 9

Reporting Summary

Peer Review File

## Source data

Source Data

## Data Availability

All genotyping and sequencing data are available through either the Accelerating Medicines Partnership in Parkinson’s Disease and Related Disorders (AMP-PDRD; https://www.amp-pd.org/) via GP2 data sharing agreements (https://amp-pdrd.org/register-for-amp-pd) or the Aligning Science Across Parkinson’s Collaborative Research Network (ASAP CRN) Cloud (cloud.parkinsonsroadmap.org) under the ASAP CRN Cloud Data Use Agreement (https://storage.googleapis.com/asap-public-assets/wayfinding/ASAP-CRN-Cloud-User-Manual.pdf). The datasets include: (i) high density Illumina Infinium global diversity array (GDA) neuro booster array (NBA) genotyping data (AMP-PDRD: gp2tier2/release6_21122023/gp2_crn/WIBR3/raw genotypes); (ii) Oxford Nanopore Technologies long-read sequencing (AMP-PDRD: gp2tier2/release6_21122023/gp2_crn/WIBR3/ONT); (iii) Pacific Biosciences long-read sequencing (AMP-PDRD: gp2tier2/release6_21122023/gp2_crn/WIBR3/PacBio); (iv) NGS and Sanger sequencing data for targeted genotyping and zygosity analysis (ASAP CRN Cloud: team-rio-hesc-targeted-ngs-mutant-zygosity; 10.5281/zenodo.19600812); (v) single cell RNA-seq data from dopaminergic neuron differentiation (ASAP CRN Cloud: team-rio-hesc-sc-rnaseq-wt-dopaminergic; 10.5281/zenodo.19600810); (vi) single cell RNA-seq data from WIBR3 hESCs following irradiation (ASAP CRN Cloud: team-rio-hesc-sc-rnaseq-irradiated; 10.5281/zenodo.19600807); single cell RNA-seq data from WIBR3-derived cortical spheroids following irradiation (ASAP CRN Cloud: team-rio-hesc-spheroids-sc-rnaseq-irradiated; 10.5281/zenodo.19600803); and (vii) whole-genome sequencing and variant calling data from the iSCORE-PD collection (ASAP CRN Cloud: team-rio-hesc-wgs-iscore-pd-genotyping-and-snps; 10.5281/zenodo.19600814). AMP-PDRD and ASAP CRN Cloud control data access to protect participant privacy, maintain compliance with ethical standards and legal regulations, and ensure secure, responsible research usage. Data are governed by a Data Use Agreement (DUA) that requires institutional approval for sensitive, granular, or high-tier data, while protecting intellectual property and ensuring data is used only for legitimate research purposes. All other materials can be found in the source data provided with this paper and in the Zenodo repository: 10.5281/zenodo.20085869. This includes (i) aCGH reports, (ii) ICC images (microglia, dopaminergic neurons, hESC), (iii) Southern blot raw film and gel images, (iv) qRT-PCR result files/analysis for dopaminergic neuron differentiation, and (v) FACS-analysis results for microglia. Source data are provided with this paper.

## Code availability

The GitHub links to all previously generated code and scripts used in this work are provided in the respective section in “Materials and Methods.” Newly developed scripts used for the mapping and analysis of scRNASeq data are available at https://github.com/ASAP-Team-Rio/iSCORE-PD_DAN_scRNASeq. (scRNA-seq analysis from dopaminergic neuron 10.5281/zenodo.20449757; scRNA-seq analysis from WIBR3-derived cortical spheroids following irradiation 10.5281/zenodo.20449790; scRNA-seq analysis from WIBR3 hESCs following irradiation 10.5281/zenodo.20449802). Scripts for SNV analysis pipeline and off-target analysis can be accessed at https://github.com/hockemeyer-ucb/pd-sv-analysis (10.5281/zenodo.20449493).
