# Supplementary material for: iSCORE-PD: an isogenic stem cell collection to research Parkinson’s disease
Source: Nat Commun. 2026 Jun 17;17:7682. doi: 10.1038/s41467-026-74355-8 (PMC13434110; doi:10.1038/s41467-026-74355-8)
Supplement: Supplementary file 1 [file 41467_2026_74355_MOESM12_ESM.pdf]

nature portfolio

Corresponding author(s): Frank Soldner & Dirk Hockemeyer

Last updated by author(s): 09/28/2025

Reporting Summary

Nature Portfolio wishes to improve the reproducibility of the work that we publish. This form provides structure for consistency and transparency in reporting. For further information on Nature Portfolio policies, see our Editorial Policies and the Editorial Policy Checklist.

Statistics

For all statistical analyses, confirm that the following items are present in the figure legend, table legend, main text, or Methods section.

n/a	Confirmed	
☐	☒	The exact sample size (*n*) for each experimental group/condition, given as a discrete number and unit of measurement
☒	☐	A statement on whether measurements were taken from distinct samples or whether the same sample was measured repeatedly
☒	☐	The statistical test(s) used AND whether they are one- or two-sided<br>*Only common tests should be described solely by name; describe more complex techniques in the Methods section.*
☒	☐	A description of all covariates tested
☒	☐	A description of any assumptions or corrections, such as tests of normality and adjustment for multiple comparisons
☒	☐	A full description of the statistical parameters including central tendency (e.g. means) or other basic estimates (e.g. regression coefficient) AND variation (e.g. standard deviation) or associated estimates of uncertainty (e.g. confidence intervals)
☒	☐	For null hypothesis testing, the test statistic (e.g. *F*, *t*, *r*) with confidence intervals, effect sizes, degrees of freedom and *P* value noted<br>*Give P values as exact values whenever suitable.*
☒	☐	For Bayesian analysis, information on the choice of priors and Markov chain Monte Carlo settings
☒	☐	For hierarchical and complex designs, identification of the appropriate level for tests and full reporting of outcomes
☒	☐	Estimates of effect sizes (e.g. Cohen's *d*, Pearson's *r*), indicating how they were calculated

Our web collection on statistics for biologists contains articles on many of the points above.

Software and code

Policy information about availability of computer code

Data collection	FACS data was collected using BD FACSDiva™ Software.
Data analysis	Genotyping data was processed using GenomeStudio (RRID:SCR_010973) and subsequent genotype calls, B-allele frequency and LogR ratio values were used for genomic integrity assessments. Genome-wide genotyping calls were compared with the PacBio HiFi WGS variants to assess large genomic events across the two data types using PLINK (v1.9, RRID:SCR_001757). The B-allele frequency and LogR ratio values were processed and plotted using the GWASTools package in R (v3.6.1, https://www.r-project.org/, DOI: 10.18129/B9.bioc.GWASTools). ONT sequencing runs were basecalled on NIH's HPC (Biowulf) using Oxford Nanopore's Guppy (v6.1.2, RRID:SCR_022353) in super accuracy mode with the dna_r9.4.1_450bps_modbases_5mc_cg_sup_prom.cfg configuration file and the –bam_out option to preserve methylation tags. The basecalled bams were then converted to fastqs using Samtools (v1.17, RRID:SCR_002105) (samtools fastq -TMm, Ml) and mapped to hg38 using Minimap2 (v2.24, RRID:SCR_018550) with ONT flags. Data from all flow cells was merged after mapping using samtools (v1.17, RRID:SCR_002105). Then, we used PEPPER-Margin-DeepVariant (v.0.8, https://github.com/kishwarshafin/pepper) to call small variants (<50bp) and phase our variant calls and alignments. We then used our phased alignment, to produce haplotype-specific methylation calls using Modbamtools (v0.4.8, https://rrazaghi.github.io/modbamtools/) and Nanopore's modbam2bed (https://github.com/epi2me-labs/modbam2bed). Lastly, structural variants (SVs) were called using Sniffles2 (v2.2, RRID:SCR_017619) with default settings. Data was mapped using Minimap2 (v2.24, RRID:SCR_018550) using PacBio flags. Small variant calls generated by Clair3 (v1.0.4) (https://github.com/HKU-BAL/Clair3) with PacBio flags and SV calls were generated by Sniffles2 (v2.2, RRID:SCR_017619). Small variants were filtered for DP>15 and GQ>20 using bcftools (v1.17, RRID:SCR_005227) and annotated with ANNOVAR (v.2022-06-08, RRID:SCR_012821) to assess the presence of potential pathogenic variants, specifically, based on variant information (GRCh38), gene-based annotations (refGene), variant identifiers (dbSNP build 150), deleteriousness and pathogenicity prediction scores (dbNSFP), genome and exome population allele frequencies (gnomAD v4.1; https://gnomad.broadinstitute.org/news/2024-04-gnomad-v4-1/)54, pathogenicity annotations (ClinVar), and dosage sensitivity annotations (ClinGen; https://clinicalgenome.org/). To assess their potential role in neurodegeneration, these variants were further annotated with known

functional categories in neurodegeneration from the KEGG (https://www.kegg.jp/) and HPO gene signature collections (https://hpo.jax.org/) (i.e., KEGG Alzheimer's and Parkinson's disease, and HPO Alzheimer's disease, Parkinsonism, and neurodegeneration) from the MSigDB (v2025.1) database (https://www.gsea-msigdb.org/gsea/msigdb/index.jsp). In addition, Alzheimer's disease and Parkinson's disease genetic risk scores (excluding UK Biobank summary statistics) were calculated to assess the cumulative risk score using plink (v2.0, RRID:SCR_001757) for disease and compared with participants from the UK Biobank diagnosed with AD and PD. Only SV calls labeled as "PASS" were kept for both ONT and PacBio data. The "PASS" SV calls were then annotated with ANNOVAR (v.2022-06-08, RRID:SCR_012821)103 and coding variants were subset. Then, we used Truvari (v4.4.0) ((https://github.com/ACEnglish/truvari) to merge structural variant calls between the ONT and PacBio datasets both for all variants as well as only coding variants. Numbers on variant type distribution were generated using SURVIVOR (v1.0.7, RRID:SCR_022995). Lastly, the SV overlaps were further annotated using SVAnna52 (v1.0.4) (https://github.com/TheJacksonLaboratory/SvAnna with the phenotype terms HP:0002180 (neurodegeneration) and HP:0000707 (abnormality of the nervous system). SVs of interest were plotted using samplot (v1.3.0) (https://github.com/ryanlayer/samplot). DNA samples were sequenced with Illumina short-read WGS at Psomagen (Rockville, MD), with a mean coverage of 30x. Data was processed using standard GP2 WGS pipelines. In brief, 150 bp paired-end reads were aligned to the human reference genome (GRCh38 build) using BWA-mem (https://github.com/lh3/bwa) following the functional equivalence pipeline. Sample processing and variant calling were performed using DeepVariant v.1.6.1. Joint-genotyping was performed using GLnexus v1.4.3 with the preset DeepVariant WGS configuration. To test whether some of the group specific SNVs could be link to potential off-target effect triggered by the CRISPR/Cas9 or prime editing reagents, all putative off-target sites were identified using Cas-OFFinder85 (https://github.com/snugel/cas-inder). Single-cell analysis of all irradiated samples was performed using Seurat v4 (RRID:SCR_016341)127 according to default parameters for normalization and integration of data sets. Droplets with more than 15% mitochondrial reads detected were excluded as poor analysis candidates due to likelihood of cell death resulting in poor RNA representation. Plots were generated using ggplot2 (RRID:SCR_014601) in R. After next-generation sequencing of 10x Genomics libraries (NovaSeq 6000), FASTQ files were processed with 10x Genomics CellRanger pipeline (v7.0.1, RRID:SCR_017344) to demultiplex and generate count matrices for each sample. Data for each sample was first filtered to remove low-quality cells (cells with fewer than 1,500 genes detected, greater than 30,000 RNA counts, and greater than 10% mitochondrial reads were removed). Filtered datasets were each processed individually with Seurat v4 (RRID:SCR_016341)127, using the SCTransform function for normalization and variance stabilization. Integration of the SCTransformed data was performed to generate a combined dataset of 10,097 cells. Graph-Pad (RRID:SCR_002798 - version 10.6.1) was used for plotting data. FlowJo software (RRID:SCR_008520 - version 10.8.0) was used to analyze FACS data collected in the BD FACSymphony™ A5 Cell Analyzer.

Code availability

The Github links to all previously generated code and scripts used in this work are provided in the respective section in Materials and Methods. Newly developed scripts used for the mapping and analysis of scRNASeq data are available at https://github.com/ASAP-Team-Rio/iSCORE-PD_DAN_scRNASeq. (scRNA-seq analysis from dopaminergic neuron DOI: 10.5281/zenodo.20449757; scRNA-seq analysis from WIBR3-derived cortical spheroids following irradiation DOI: 10.5281/zenodo.20449790; scRNA-seq analysis from WIBR3 hESCs following irradiation DOI: 10.5281/zenodo.20449802). Scripts for SNV analysis pipeline and Off-target analysis can be accessed at https://github.com/hockemeyer-ucb/pd-sv-analysis (DOI: 10.5281/zenodo.20449493).

For manuscripts utilizing custom algorithms or software that are central to the research but not yet described in published literature, software must be made available to editors and reviewers. We strongly encourage code deposition in a community repository (e.g. GitHub). See the Nature Portfolio guidelines for submitting code & software for further information.

Data

Policy information about availability of data

All manuscripts must include a data availability statement. This statement should provide the following information, where applicable:

- Accession codes, unique identifiers, or web links for publicly available datasets
- A description of any restrictions on data availability
- For clinical datasets or third party data, please ensure that the statement adheres to our policy

All genotyping and sequencing data is available through either the Accelerating Medicines Partnership in Parkinson's Disease and Related Disorders (AMP-PDRD; https://www.amp-pd.org/) via GP2 data sharing agreements (https://amp-pdrd.org/register-for-amp-pd) or the Aligning Science Across Parkinson's Collaborative Research Network (ASAP CRN) Cloud (cloud.parkinsonsroadmap.org) under the ASAP CRN Cloud Data Use Agreement (https://storage.googleapis.com/asap-public-assets/wayfinding/ASAP-CRN-Cloud-User-Manual.pdf).

The datasets include: (i) high density Illumina Infinium Global Diversity Array (GDA) Neuro booster Array (NBA) genotyping data (AMP-PDRD: gp2tier2/release6_21122023/gp2_crn/WIBR3/raw genotypes); (ii) Oxford Nanopore Technologies long-read sequencing (AMP-PDRD: gp2tier2/release6_21122023/gp2_crn/WIBR3/ONT); (iii) Pacific Biosciences long-read sequencing (AMP-PDRD: gp2tier2/release6_21122023/gp2_crn/WIBR3/PacBio); (iv) NGS and Sanger sequencing data for targeted genotyping and zygosity analysis (ASAP CRN Cloud: team-rio-hesc-targeted-ngs-mutant-zygosity; DOI: 10.5281/zenodo.19600812); (v) single cell RNA-seq data from dopaminergic neuron differentiation (ASAP CRN Cloud: team-rio-hesc-sc-rnaseq-wt-dopaminergic; DOI: 10.5281/zenodo.19600810); (vi) single cell RNA-seq data from WIBR3 hESCs following irradiation (ASAP CRN Cloud: team-rio-hesc-sc-rnaseq-irradiated; DOI: 10.5281/zenodo.19600807); single cell RNA-seq data from WIBR3-derived cortical spheroids (ASAP CRN Cloud: team-rio-hesc-spheroids-sc-rnaseq-irradiated; DOI: 10.5281/zenodo.19600803); and (vii) Whole-genome sequencing and variant calling data from the iSCORE-PD collection (ASAP CRN Cloud: team-rio-hesc-wgs-iscore-pd-genotyping-and-snps; DOI: 10.5281/zenodo.19600814).

AMP-PDRD and ASAP CRN Cloud control data access to protect participant privacy, maintain compliance with ethical standards and legal regulations, and ensure secure, responsible research usage. Data is governed by a Data Use Agreements (DUA) that requires institutional approval for sensitive, granular, or high-tier data, while protecting intellectual property and ensuring data is used only for legitimate research purposes.

All other materials can be found in the source data provided with this paper and in the Zenodo repository: 10.5281/zenodo.20085869. This includes (i) aCGH reports, (ii) ICC images (microglia, DA neurons, hESC), (iii) southern blot raw film and gel images, (iv) qRT-PCR result files/analysis for dopaminergic neuron differentiation, and (v) FACS-analysis results for microglia.

Source data are provided with this paper.

Research involving human participants, their data, or biological material

Policy information about studies with human participants or human data. See also policy information about sex, gender (identity/presentation), and sexual orientation and race, ethnicity and racism.

Reporting on sex and gender	For this study we used a female human embryonic stem cell (hESC) line (WIBR3; NIH approval number NIHhESC-10-0079).<br><br>As outlined in our manuscript, our rationale for choosing the female hESC line WIBR3 was: (1) concerns regarding somatic mutations in donors prior to reprogramming, as well as reprogramming-induced genetic alterations, incomplete epigenetic reprogramming, and aberrant genomic imprinting in iPSCs; (2) the importance of including cell lines from both sexes, as male iPSCs are often favored due to concerns about X-chromosome erosion; (3) the fact that WIBR3 has been shown to maintain a stable karyotype over extended culture and has been widely used in modeling human diseases, including PD and (4) access to cells at an early-passage after derivation (P14).
Reporting on race, ethnicity, or other socially relevant groupings	The parental cell line used in this study is of European descent (RRID:CVCL_9767, NIH approval number NIHhESC-10-0079).
Population characteristics	NA
Recruitment	NA
Ethics oversight	NA

Note that full information on the approval of the study protocol must also be provided in the manuscript.

Field-specific reporting

Please select the one below that is the best fit for your research. If you are not sure, read the appropriate sections before making your selection.

☒ Life sciences ☐ Behavioural & social sciences ☐ Ecological, evolutionary & environmental sciences

For a reference copy of the document with all sections, see nature.com/documents/nr-reporting-summary-flat.pdf

Life sciences study design

All studies must disclose on these points even when the disclosure is negative.

Sample size	Sample size is only relevant to specific assays for this publication. Sample size was determined on necessary statistical power.
Data exclusions	NA
Replication	NA
Randomization	NA
Blinding	NA

Reporting for specific materials, systems and methods

We require information from authors about some types of materials, experimental systems and methods used in many studies. Here, indicate whether each material, system or method listed is relevant to your study. If you are not sure if a list item applies to your research, read the appropriate section before selecting a response.

Materials & experimental systems

n/a	Involved in the study	
☐	☒	Antibodies
☐	☒	Eukaryotic cell lines
☒	☐	Palaeontology and archaeology
☒	☐	Animals and other organisms
☒	☐	Clinical data
☒	☐	Dual use research of concern
☒	☐	Plants

Methods

n/a	Involved in the study	
☐	☐	ChIP-seq
☐	☒	Flow cytometry
☐	☐	MRI-based neuroimaging

Antibodies

Antibodies used	OCT4 (DSHB - PCRP-POU5F1-1D2), SSEA4 (DSHB - MC-813-70 (SSEA-4)), TH (Pel-Freez Biologicals - p60101-150), FOXA2 (R&D Systems - AF2400)
Validation	OCT4: https://dshb.biology.uiowa.edu/PCRP-POU5F1-1D2<br>SSEA4: https://dshb.biology.uiowa.edu/MC-813-70-SSEA-4<br>TH: https://www.pel-freez.com/sheep-tyrosine-hydroxylase-p60101-150<br>FOXA2: https://www.rndsystems.com/products/human-hnf-3beta-foxa2-antibody_af2400

Eukaryotic cell lines

Policy information about cell lines and Sex and Gender in Research

Cell line source(s)	Whitehead Institute for Biomedical Research; Boston; USA
Authentication	WIBR3; NIH approval number NIHhESC-10-0079, CVCL_9767 (Cellosaurus), WIBRe001-A (hPSCreg)
Mycoplasma contamination	Negative
Commonly misidentified lines<br>(See ICLAC register)	NA

Plants

Seed stocks	*Report on the source of all seed stocks or other plant material used. If applicable, state the seed stock centre and catalogue number. If plant specimens were collected from the field, describe the collection location, date and sampling procedures.*
Novel plant genotypes	*Describe the methods by which all novel plant genotypes were produced. This includes those generated by transgenic approaches, gene editing, chemical/radiation-based mutagenesis and hybridization. For transgenic lines, describe the transformation method, the number of independent lines analyzed and the generation upon which experiments were performed. For gene-edited lines, describe the editor used, the endogenous sequence targeted for editing, the targeting guide RNA sequence (if applicable) and how the editor was applied.*
Authentication	*Describe any authentication procedures for each seed stock used or novel genotype generated. Describe any experiments used to assess the effect of a mutation and, where applicable, how potential secondary effects (e.g. second site T-DNA insertions, mosiacism, off-target gene editing) were examined.*

ChIP-seq

Data deposition

☐ Confirm that both raw and final processed data have been deposited in a public database such as GEO.

☐ Confirm that you have deposited or provided access to graph files (e.g. BED files) for the called peaks.

Data access links<br>*May remain private before publication.*	*For "Initial submission" or "Revised version" documents, provide reviewer access links. For your "Final submission" document, provide a link to the deposited data.*
Files in database submission	*Provide a list of all files available in the database submission.*
Genome browser session<br>(e.g. UCSC)	*Provide a link to an anonymized genome browser session for "Initial submission" and "Revised version" documents only, to enable peer review. Write "no longer applicable" for "Final submission" documents.*

Methodology

Replicates	*Describe the experimental replicates, specifying number, type and replicate agreement.*
Sequencing depth	*Describe the sequencing depth for each experiment, providing the total number of reads, uniquely mapped reads, length of reads and whether they were paired- or single-end.*
Antibodies	*Describe the antibodies used for the ChIP-seq experiments; as applicable, provide supplier name, catalog number, clone name, and lot number.*
Peak calling parameters	*Specify the command line program and parameters used for read mapping and peak calling, including the ChIP, control and index files used.*
Data quality	*Describe the methods used to ensure data quality in full detail, including how many peaks are at FDR 5% and above 5-fold enrichment.*
Software	*Describe the software used to collect and analyze the ChIP-seq data. For custom code that has been deposited into a community repository, provide accession details.*

Flow Cytometry

Plots

Confirm that:

- [x] The axis labels state the marker and fluorochrome used (e.g. CD4-FITC).
- [x] The axis scales are clearly visible. Include numbers along axes only for bottom left plot of group (a 'group' is an analysis of identical markers).
- [x] All plots are contour plots with outliers or pseudocolor plots.
- [x] A numerical value for number of cells or percentage (with statistics) is provided.

Methodology

Sample preparation	https://www.protocols.io/view/microglia-validation-by-facs-analysis-c66vzhe6
Instrument	BD FACSymphony™ A5 Cell Analyzer
Software	BD FACSDiva™ Software
Cell population abundance	Abundance of the desired population was determined from the original sample by gating single cells and using calcein violet as a marker for viable cells.
Gating strategy	SSC-A/FSC-A (cells minus debris) --> SSC-A/SSC-W (single cells) --> FSC-A/FSC-W (single cells) --> SSC-A/UV446-A(calcein violet positive population) --> PE/APC.

- [x] Tick this box to confirm that a figure exemplifying the gating strategy is provided in the Supplementary Information.

Magnetic resonance imaging

Experimental design

Design type	*Indicate task or resting state; event-related or block design.*
Design specifications	*Specify the number of blocks, trials or experimental units per session and/or subject, and specify the length of each trial or block (if trials are blocked) and interval between trials.*
Behavioral performance measures	*State number and/or type of variables recorded (e.g. correct button press, response time) and what statistics were used to establish that the subjects were performing the task as expected (e.g. mean, range, and/or standard deviation across subjects).*

Acquisition

Imaging type(s)	*Specify: functional, structural, diffusion, perfusion.*
Field strength	*Specify in Tesla*
Sequence & imaging parameters	*Specify the pulse sequence type (gradient echo, spin echo, etc.), imaging type (EPI, spiral, etc.), field of view, matrix size, slice thickness, orientation and TE/TR/flip angle.*
Area of acquisition	*State whether a whole brain scan was used OR define the area of acquisition, describing how the region was determined.*

Diffusion MRI ☐ Used ☐ Not used

Preprocessing

Preprocessing software	*Provide detail on software version and revision number and on specific parameters (model/functions, brain extraction, segmentation, smoothing kernel size, etc.).*
Normalization	*If data were normalized/standardized, describe the approach(es): specify linear or non-linear and define image types used for transformation OR indicate that data were not normalized and explain rationale for lack of normalization.*
Normalization template	*Describe the template used for normalization/transformation, specifying subject space or group standardized space (e.g. original Talairach, MNI305, ICBM152) OR indicate that the data were not normalized.*
Noise and artifact removal	*Describe your procedure(s) for artifact and structured noise removal, specifying motion parameters, tissue signals and physiological signals (heart rate, respiration).*
Volume censoring	*Define your software and/or method and criteria for volume censoring, and state the extent of such censoring.*

Statistical modeling & inference

Model type and settings | *Specify type (mass univariate, multivariate, RSA, predictive, etc.) and describe essential details of the model at the first and second levels (e.g. fixed, random or mixed effects; drift or auto-correlation).*

Effect(s) tested | *Define precise effect in terms of the task or stimulus conditions instead of psychological concepts and indicate whether ANOVA or factorial designs were used.*

Specify type of analysis: ☐ Whole brain ☐ ROI-based ☐ Both

Statistic type for inference | *Specify voxel-wise or cluster-wise and report all relevant parameters for cluster-wise methods.*

(See Eklund et al. 2016)

Correction | *Describe the type of correction and how it is obtained for multiple comparisons (e.g. FWE, FDR, permutation or Monte Carlo).*

Models & analysis

n/a	Involved in the study
☐	☐ Functional and/or effective connectivity
☐	☐ Graph analysis
☐	☐ Multivariate modeling or predictive analysis

Functional and/or effective connectivity | *Report the measures of dependence used and the model details (e.g. Pearson correlation, partial correlation, mutual information).*

Graph analysis | *Report the dependent variable and connectivity measure, specifying weighted graph or binarized graph, subject- or group-level, and the global and/or node summaries used (e.g. clustering coefficient, efficiency, etc.).*

Multivariate modeling and predictive analysis | *Specify independent variables, features extraction and dimension reduction, model, training and evaluation metrics.*
