# Supplementary material for: iSCORE-PD: an isogenic stem cell collection to research Parkinson’s disease
Source: Nat Commun. 2026 Jun 17;17:7682. doi: 10.1038/s41467-026-74355-8 (PMC13434110; doi:10.1038/s41467-026-74355-8)
Supplement: Supplementary file 2 [file 41467_2026_74355_MOESM13_ESM.pdf]

nature portfolio

Peer Review File

iSCORE-PD: an isogenic stem cell collection to research Parkinson's Disease

Corresponding Author: Dr Frank soldner

This file contains all reviewer reports in order by version, followed by all author rebuttals in order by version.

Version 0:

Reviewer comments:

Reviewer #1

(Remarks to the Author)
Summary
Busquets, Li, Syed, Jerez, Dunnack, and colleagues describe an isogenic human embryonic stem cell-based resource for modelling Parkinson's Disease (PD). To do so, they first subcloned and characterised the genomic integrity of the parental WIBR3 hESC line, then characterised the efficiency of dopaminergic (DA) neuron production from each subclone by single-cell sequencing and comparison to previous datasets of iPSC-derived DA neurons, as well as microglial-like cells as revealed by marker gene analysis. The WIBR3 cell line was then subjected to CRISPR/Cas9-, TALEN-, and prime-based gene editing to generate 65 clonal cell lines carrying homozygous or heterozygous mutations in 13 causal/high-risk variants across 11 PD-linked genes (e.g., SNCA, LRRK2, GBA1), alongside non-edited subclones and cells that had undergone gene editing protocols but retained the parental genotype at the targeted locus. Edited clones were expanded, sequenced at the targeted locus and characterised for pluripotency, genomic stability, loss of heterozygosity, and whole genome sequencing to generate the iSCORE-PD resource. This deep characterisation led to the identification of clone-specific genetic variants that were either present in the founding cell population and/or had arisen during gene editing. The authors suggest that off-target effects did not substantially contribute to these clonal variants, which were instead likely derived in vitro.

The manuscript has many strengths. It is clearly written, and the work is logically structured. The deep characterisation of parental and gene-edited cell lines makes this resource very likely to be useful to the community. However, there are some areas where the manuscript could be improved:

Major comments
• The authors articulate the benefits of using WIBR3, since it is a female cell line that complements the male lines used in other large-scale gene editing initiatives, and is derived from the inner cell mass and therefore likely does not suffer from somatic mutations that have accumulated in parental fibroblasts prior to reprogramming. However, regulatory barriers can restrict embryonic stem cell research in many countries. How accessible will this hESC-based resource be to investigators around the world? Based on consent forms and MTAs, what restrictions are there that might limit the distribution and modification of these cell lines, or their use in commercial rather than academic settings? Do MTAs have to be negotiated with the original institution and/or WiCell, or are gene-edited clones obtainable without this red tape?
• One strength of the study is the detailed genetic characterisation of the parental (subcloned) WIBR3 cell line and its gene-edited derivates by long-read whole genome sequencing, and analysis of this sequence information for potentially deleterious CNVs and SNVs. The authors very appropriately make the WGS data available to the community for further study, but most groups will not have the capacity to carry out their own functional annotation of these data. Since ClinVar is not a comprehensive database, users of this cell resource would be interested in other potentially deleterious or PD-modifying variants. What deleterious SNVs are identified by bioinformatic predictors such as CADD, FATHMM, REVEL, or many other that have been developed? Are there any variants that have been identified in COSMIC? Are there copy number or sequence variants present in dosage-sensitive genes?
• The authors took great care to characterise the genomic integrity of gene-edited cell clones. However, there can be different constellations of founder mutations in clones derived from a heterogeneous starting population, and/or epigenetic variability between clones (or other factors) can alter differentiation potential in a manner not directly related to on-target or off-target mutations. How do the authors plan to account for these effects to ensure that they do not potentially mask the effects of the engineered mutations they wish to study? Some consortia such as iNDI have addressed this issue by

correcting the mutant allele back to a wild-type allele in ‘revertant’ clones. Do the authors have any plans to generate such clones or use alternative approaches to increase the value of their resource? If not, why?
• Validation that at least one of the gene-edited clones recapitulates PD-relevant phenotypes known to exist in iPSC-derived cellular models would increase reader confidence in the utility of the resource.
• It is not totally clear if all gene editing experiments used the WIBR3 cell line or a subclone of WIBR3, given the deep characterisation of sub-clones and the narrative flow. If a non-subcloned culture of WIBR3 formed the basis of gene editing experiments, what’s the rationale for that choice given that subclones had been previously generated and functionally validated? We agree with the authors’ interpretation that shared variants among gene-edited clones were likely due to founder effects. Depending on the degree of the heterogeneity present in the WIBR3 culture, it difficult to accurately assess the ‘mutation rate’ that the authors propose, since unique variants present at low fraction among the starting population can become fixed during cloning. To assess mutation rate accurately, many clonal lines would have to be generated and propagated from a sub-clone, not the parental WIBR3 line. These laborious studies seem beyond the scope of the study, so it seems most appropriate to tone down the strength of statements to those that are clearly supported, and to clearly articulate potential confounds to the reader. The value of the manuscript mainly derives from the resource and the careful QC done on clones, and the section on genetic analysis could be shortened and clarified without loss of impact.

Minor comments
• Though data from the differentiation WIBR3 subclones S1-3 is shown (Fig. S3) and suggest some variability in the production of clusters 3 and 4, these data are not quantified. Similarly, microglial production experiments from sub-clones rely on a few markers and no quantification is shown.
• The authors state that there is a high level of genomic instability (Page 13), which could be misleading to the reader, since previous studies suggest that other cell lines accumulate mutations in culture and upon gene editing at similar or higher rates than hPSCs (10.1038/s41467-020-15271-3).
• Having line numbers in the manuscript would have helped point out specific comments in the text.
• Other groups have shown that “perfect isogenic clones do not exist in vitro”, and have argued for using recently derived sub-clones to reduce sequence heterogeneity and resulting founder effects. While these are important confirmation by the authors, it’s over-reaching to declare them as novel Major Findings.
• Readers might benefit from a brief clarification of the clinical relevance of each type of introduced mutation, perhaps summarised in tabular form in a main figure (e.g. extension of Fig 3C or Fig. 4C), to provide an overview of the resource.
• How do the authors plan to ensure that gene-edited cell clones show similar differentiation potential as the parental cell line or sub-clones? It’s not reasonable to request that the authors do this for all clones, but articulating the strategy by which they will provide evidence of validation to the broader community would be helpful.
• What are the plans to extend the iSCORE-PD resource to other genetic backgrounds, or to model additional PD-associated genetic variants?

(Remarks on code availability)
I do not have the necessary expertise to review the code.

Reviewer #3

(Remarks to the Author)
The article represents a significant contribution to Parkinson's disease (PD) research by providing an innovative resource—a collection of isogenic stem cell lines carrying PD-associated mutations (iSCORE-PD). The study is executed at a high scientific level, with meticulous experimental design and comprehensive analysis. This work holds great value for the scientific community, as it offers a standardized platform for studying the molecular mechanisms of PD, based on the well-characterized human embryonic stem cell line WIBR3.

The authors employed advanced genome-editing techniques (CRISPR/Cas9, TALEN, prime editing) to generate 65 cell lines with mutations in 11 PD-related genes. This enables the study of specific mutations under controlled conditions. Notably, the authors demonstrated that prime editing is more precise than CRISPR/Cas9 for introducing mutations into human pluripotent stem cells (hPSCs).

The study included rigorous validation steps, such as whole-genome sequencing (combining ONT, PacBio, and Illumina methods), karyotype analysis, and pluripotency verification, ensuring the reliability of the results. Additionally, the authors thoroughly investigated sources of genetic variability, which is crucial for interpreting phenotypic differences.

The authors confirmed the ability of the parental WIBR3 cell line and its subclones to differentiate into dopaminergic neurons. Through their comprehensive work, they demonstrated the advantages of their cell collection for studying the molecular mechanisms of PD pathogenesis caused by mutations in key target genes in vitro under controlled conditions.

The findings were supported by extensive imaging data with clear and well-labeled annotations.

Since the cell collection was derived from female embryonic stem cells, the authors should consider the phenomenon of X-chromosome erosion (Kilens et al., 2018, DOI: 10.1038/s41467-017-02107-w; Vallot et al., 2015, DOI: 10.1016/j.stem.2015.03.016). During in vitro culturing of female cells, partial or complete reactivation of the inactive X-chromosome frequently occurs, which may affect cell proliferation and differentiation. This phenomenon is important to account for, as it could play a key role in the development of pathogenic phenotypes in PD modeling in vitro. Authors should discuss this aspect in the article.

The study also has some limitations. For example, in describing the hESC culture conditions, the authors stated "95% CO_2 and 5% O_2" which is incorrect. Furthermore, the authors did not demonstrate the ability of the edited hESCs to differentiate into dopaminergic neurons. This ability should be shown at least on several lines. This aspect is important when modeling PD.

Overall, the article marks an important step in PD research by providing a unique resource for scientific investigation. The work is distinguished by its high methodological rigor and data transparency. Despite some limitations, this cell collection will serve as a valuable tool for understanding PD mechanisms and developing novel therapeutic strategies.

(Remarks on code availability)
The results of the work are reproducible and the code is a usable resource for the community. The README file contains sufficient instructions to install and run the application.

Reviewer #4

(Remarks to the Author)
The authors evaluated the characteristics of human embryonic stem (hES) cells derived from a Caucasian female (WIBR3) and employed multiple genome editing techniques to introduce pathogenic mutations associated with hereditary Parkinson's disease, thereby generating an isogenic collection of Parkinson's disease hES cells. By thoroughly characterizing the introduced mutations, the authors provide a valuable resource for Parkinson’s disease research utilizing stem cells. Furthermore, while previous studies have separately reported off-target mutation rates associated with genome editing and the insertion of mutations over successive passages, this study stands out by systematically conducting direct comparative analyses. These findings represent an important contribution to the field. The presented data appear robust and have the potential to significantly advance Parkinson’s disease research. However, I would like to pose a few questions and offer some suggestions.
１． The detailed genetic analysis presented in this study supports the validity of the genome-edited lines and the parental WIBR3 cells as a hereditary Parkinson's disease model and its corresponding control. However, considering the broader context of reference cell utility in stem cell research (DOI: 10.1016/j.stem.2025.05.004), the justification for selecting WIBR3 as the parental cell line appears somewhat weak. If there is a specific reason for selecting WIBR3 over other available cell lines, please provide an explanation.
２． Related to the previous question, would it be feasible to evaluate the similarity between the WIBR3 cells and KOLF2.1J cells, which are currently considered a reference cell line, in terms of genomic sequence, gene expression, and differentiation efficiency? Given the differences in donor origin and the inherent distinctions between iPSCs and ESCs, I understand that such a comparison may be challenging. However, conducting this analysis could further enhance the value of the generated cell library.
３． In Figure 4D, overlapping dots of similar colors make it difficult to distinguish individual data points. To effectively support the claims presented in this figure, rather than displaying a single shared trend line, it would be necessary to illustrate that EWT_S1_8 and the other genome-edited lines exhibit similar tendencies.

(Remarks on code availability)

Reviewer #5

(Remarks to the Author)
I co-reviewed this manuscript with one of the reviewers who provided the listed reports. This is part of the Nature Communications initiative to facilitate training in peer review and to provide appropriate recognition for Early Career Researchers who co-review manuscripts.

(Remarks on code availability)

Version 1:

Reviewer comments:

Reviewer #1

(Remarks to the Author)
The authors provide an updated manuscript describing an isogenic series of cell lines on the WIBR3 genetic background carrying mutations associated with Parkinson's Disease (PD). This resource is likely to be of considerable interest to the research community modelling PD with human embryonic stem cells (hESCs). This revised manuscript addresses some of the key concerns noted by multiple reviewers, but a few points remain.
1) If this is to be a stand-alone manuscript describing the generation of a resource meant to be of value to the community seeking to model PD, it seems critical to demonstrate that the resource can indeed usefully model PD. The authors state that in-depth characterisation of the resource is outside of the scope of this study and instead offer some analysis in separate

pre-prints. While in-depth characterisation of all of the mutant lines would indeed be excessive, it seems reasonable to either include some of the work cited in the preprints in this manuscript, or to include a small handful of additional examples to show that the resource is indeed useful for modelling PD by recapitulating phenotypes previously shown in the literature.
2) For clarity, the inaccessibility of the resource to investigators in countries where embryonic stem cell research is not permitted would be more appropriate to include in the manuscript main text when discussing potential limitations, and not just in the Methods section. Similarly, given that the parental cell line was not subcloned prior to gene editing, the issue of many founder mutations becoming fixed along with the intended mutation during the gene editing process, including potentially cancer-associated mutations that were not tested for directly but are known to recur during stem cell culture, should be clearly pointed out to readers as potential limitations in the Discussion.

(Remarks on code availability)

Reviewer #3

(Remarks to the Author)
Dear editor and authors of the article "iSCORE-PD: An Isogenic Stem Cell Collection to Research Parkinson's Disease."

The authors of the article responded in detail to the comments of all reviewers and made appropriate changes to the text. The changes and clarifications undoubtedly improved the article. I have no further comments.

(Remarks on code availability)
Unfortunately, I don't have enough competence to evaluate the code.

Reviewer #4

(Remarks to the Author)
The authors have appropriately addressed the concerns I previously raised, and the revised manuscript shows clear improvement. I appreciate their thoughtful responses and the additional data provided.

(Remarks on code availability)

Reviewer #5

(Remarks to the Author)
I co-reviewed this manuscript with one of the reviewers who provided the listed reports. This is part of the Nature Communications initiative to facilitate training in peer review and to provide appropriate recognition for Early Career Researchers who co-review manuscripts.

(Remarks on code availability)

Version 2:

Reviewer comments:

Reviewer #1

(Remarks to the Author)
I thank the authors for their thoughtful point-by-point response and for incorporating suggested changes that have made the revised manuscript of greater utility to the community. I have no further comments and wish them good luck with their future studies with this helpful resource.

(Remarks on code availability)

Open Access This Peer Review File is licensed under a Creative Commons Attribution 4.0 International License, which permits use, sharing, adaptation, distribution and reproduction in any medium or format, as long as you give appropriate credit to the original author(s) and the source, provide a link to the Creative Commons license, and indicate if changes were made.
In cases where reviewers are anonymous, credit should be given to 'Anonymous Referee' and the source.
The images or other third party material in this Peer Review File are included in the article's Creative Commons license, unless indicated otherwise in a credit line to the material. If material is not included in the article's Creative Commons license and your intended use is not permitted by statutory regulation or exceeds the permitted use, you will need to obtain permission directly from the copyright holder.
To view a copy of this license, visit https://creativecommons.org/licenses/by/4.0/

Dear Reviewers,

We would like to thank the reviewers for their insightful and constructive feedback. We appreciate the reviewers' thoughtful evaluation of our work and have carefully revised the manuscript to address all points raised. In response to the reviewers' comments, we have incorporated several new analyses and datasets, as detailed in our point-by-point responses below. We believe these revisions substantially strengthen the manuscript. The main new data and revisions are summarized below.

One main concern raised by the reviewers was to ensure that gene-edited cell clones retain similar differentiation potential to the parental line and to validate whether these clones recapitulate Parkinson's disease (PD)-relevant phenotypes. As discussed in our detailed response below, we consider in-depth functional characterization beyond the scope of this resource paper. However, in parallel work, we have analyzed global gene expression and pre-mRNA splicing in in vitro-differentiated, midbrain dopaminergic neurons carrying pathogenic PD mutations in PRKN, SNCA, LRRK2, PINK1, DNAJC6, FBXO7, SYNJ1, DJ1, VPS13C, ATP13A2, and GBA1 indicating the robust in vitro differentiation of all cell lines. These analyses revealed mutation-associated splicing alterations overlapping with those observed in postmortem PD brains (Lee et al., 2025; bioRxiv: https://doi.org/10.1101/2024.02.28.582420). In addition, we used *in vitro*–differentiated midbrain dopaminergic neurons carrying the *LRRK2* G2019S mutation to corroborate findings from PD patient brain samples showing that TDP-43 loss of function drives aberrant splicing in PD (Brenton et al., 2025; bioRxiv: https://doi.org/10.1101/2025.09.04.673943). This information, including references has been added to the revised manuscript (Page 15, lines 576–583).

Reviewer 1 also suggested performing a more comprehensive analysis of potentially pathogenic SNVs and indels in the parental WIBR3 cell line, noting that ClinVar alone is not exhaustive. Following the reviewer's suggestion, we performed an expanded analysis using bioinformatic predictor tools, including REVEL (rare exome variant ensemble learner), a machine learning–based pathogenicity predictor integrating multiple commonly used scores (PolyPhen-2, MutationTaster, SIFT, MutPred, CADD, etc.) and CADD (combined annotation-dependent depletion). We also evaluated gene dosage sensitivity of all loss-of-function variants using the Clinical Genome Resource. This analysis revealed no potentially pathogenic variants mapping to genes in KEGG Parkinson's or Alzheimer's disease pathways or to the human phenotype ontology (HPO) terms for Parkinsonism or Alzheimer's disease. Only two common, and therefore unlikely pathogenic variants in *UQCRHL* (rs61744357) and *RREB1* (rs1334576), and one rare heterozygous variant in *TREX1* (rs760838030), have previously been linked to neurodegeneration phenotypes. The *TREX1* missense variant is reported to cause disease only in the homozygous or compound heterozygous state (Ekinci et al., 2017). Overall, these results indicate that the parental WIBR3 cells do not harbor variants that would limit their utility for modeling neurodegenerative diseases. This analysis is now presented in Supplementary Data 2 and discussed in the revised manuscript (Page 4-5, lines 147 – 177).

In addition, we have:

1. Included Nathalie Pochet as additional author as she contributed to the classification of pathogenic variants which is included in the revised manuscript. We have included the author list change form signed by all authors in the submission.
2. Included the quantification of both the proportion and number of cells within each cluster following the differentiation into dopaminergic neurons for all WIBR3 subclones in Supplementary Figure 3A.
3. Included FACS-based quantification of microglial differentiation for each subclone; demonstrating that a high percentage of cells express the key mature microglial markers CX3CR1 and P2RY12 (>90% CX3CR1+/P2RY12+). These new results indicate that all WIBR3 subclones efficiently and robustly generate mature iMGs are presented in Figure 2H–J, Supplementary Figure 5A and discussed in the revised manuscript (Page 7, lines 242-248).
4. Included additional data supporting our estimate of a culture-associated mutation rate of ~20 mutations per passage (Page 11, lines 407–408).
5. Included a discussion of X-chromosome erosion in female hPSCs and its potential impact on X-linked gene expression and cellular phenotypes (Page 15, lines 558-565).
6. Revised wording referring to WIBR3 as a "reference" cell line, acknowledging that hESCs cannot formally be a reference lines, as their donor health status cannot be fully predicted.
7. Expanded Table 1 to include additional information on the clinical relevance of each introduced mutation. Detailed descriptions and supporting citations are provided in Supplementary Note 1.
8. Overall, we revised the manuscript to improve clarity and ensure adherence to formatting requirements. In particular, we have included a revised abstract.

Below, we provide a point-by-point response to the reviewer comments. All corresponding revisions are highlighted in blue in the revised manuscript.

REVIEWER COMMENTS

Reviewer #1

Reviewer 1 - comment 1 (major):

The authors articulate the benefits of using WIBR3, since it is a female cell line that complements the male lines used in other large-scale gene editing initiatives, and is derived from the inner cell mass and therefore likely does not suffer from somatic mutations that have accumulated in parental fibroblasts prior to reprogramming. However, regulatory barriers can restrict embryonic stem cell research in many countries. How accessible will this hESC-based resource be to investigators around the world? Based on consent forms and MTAs, what restrictions are there that might limit the distribution and modification of these cell lines, or their use in commercial rather than academic settings? Do MTAs have to be negotiated with the original institution and/or WiCell, or are gene-edited clones obtainable without this red tape?

Response:

We fully agree with the reviewer that accessibility of such stem cell collections is essential for their value and broad adaption as research tools within the scientific community. As outlined in our manuscript,

the parental WIBR3 cell line is an NIH-approved human embryonic stem cell line, enabling use in the US with governmental funding and therefore is already widely applied to model human diseases, including Parkinson's disease. We apologize for not providing sufficient details regarding the MTA process in our initial submission. All lines in this isogenic collection will be made available for research purposes to academic and non-profit institutions through the WiCell Research Institute (https://www.wicell.org/), supported by the Aligning Science Across Parkinson's (ASAP) initiative and the Michael J. Fox Foundation (MJFF). Material transfer agreements (MTAs) will be required and administered centrally by WiCell for academic and other non-commercial requests. Requests for commercial use of these lines must be negotiated directly with the provider(s). As the reviewer correctly points out, the use of human embryonic stem cells is subject to country-specific regulations and requires local approvals (e.g. IRB and ESGRO in the US) of the relevant cell line(s). This information has been added to the *Methods* section of the revised manuscript under "Cell line availability" (Page 26, line 984 – 990).

Reviewer 1 - comment 2 (major):

One strength of the study is the detailed genetic characterisation of the parental (subcloned) WIBR3 cell line and its gene-edited derivates by long-read whole genome sequencing, and analysis of this sequence information for potentially deleterious CNVs and SNVs. The authors very appropriately make the WGS data available to the community for further study, but most groups will not have the capacity to carry out their own functional annotation of these data. Since ClinVar is not a comprehensive database, users of this cell resource would be interested in other potentially deleterious or PD-modifying variants. What deleterious SNVs are identified by bioinformatic predictors such as CADD, FATHMM, REVEL, or many other that have been developed? Are there any variants that have been identified in COSMIC? Are there copy number or sequence variants present in dosage-sensitive genes?

Response:

We thank the reviewer for this comment. As suggested, we have included a more comprehensive analysis to determine the presence of potentially pathogenic variants in the parental WIBR3 cell line, noting that ClinVar (https://www.ncbi.nlm.nih.gov/clinvar/) alone is not exhaustive. Following the reviewer's suggestion, we evaluated gene dosage sensitivity of predicted loss-of-function variants using the Clinical Genome Resource (www.clinicalgenome.org). These analyses revealed 47 variants reported as pathogenic or with conflicting interpretations of pathogenicity and four variants with evidence of affecting dosage-sensitive genes or being located within dosage-sensitive genomic regions (Supplementary Data 2). Importantly, none of these potential pathogenic SNVs or dosage sensitive loss-of-function mutations mapped to genes included in the KEGG signatures for Parkinson's or Alzheimer's disease, or to the phenotype terms HP:0001300 (Parkinsonism), HP:0002511 (Alzheimer's disease) and HP:0002180 (neurodegeneration) (Supplementary Data 2), except for a heterozygous variant in TREX1 (rs760838030; NM_033629.6(TREX1):c.340C>T [p.Arg114Cys]). This missense mutation is reported only in a homozygous or compound heterozygous state in individuals with autosomal-recessive Aicardi–Goutières syndrome or chilblain lupus, autoinflammatory syndromes that can manifest with cerebral vasculitis (Ekinci et al., 2017).

Although ClinVar is currently a widely accepted standard for assessing the relevance of genomic variants, we agree with the reviewer that the database does not provide pathogenicity information for all SNVs. As suggested by the reviewer, we also assessed potential pathogenic or deleterious variants using bioinformatic-based predictor tools (Supplementary Data 2). REVEL (rare exome variant ensemble learner), a machine-learning–based pathogenicity predictor specifically designed to evaluate missense mutations, that integrates commonly used pathogenicity scores and conservation metrics into one model (e.g., PolyPhen-2, MutationTaster, SIFT, MutPred, CADD, etc.), identified 55 variants with potential pathogenicity (cutoff ≥ 0.50, excluding variants classified as benign in ClinVar). In addition, using CADD (combined annotation-dependent depletion), a widely used broader and less stringent predictor, we identified 833 potentially deleterious variants (CADD-PHRED ≥ 20, excluding variants classified as benign in ClinVar), the majority of which are common (gnomAD AF ≥ 0.01). Importantly, none of the REVEL or CADD nominated variants mapped to genes included in the KEGG signatures for Parkinson's or Alzheimer's disease, or to the phenotype terms HP:0001300 (Parkinsonism) or HP:0002511 (Alzheimer's disease). Only two additional common, and therefore unlikely pathogenic variants, one in UQCRHL (rs61744357, gnomAD = 0.1242) and one in RREB1 (rs1334576, gnomAD = 0.427), with CADD-PHRED ≥ 20 but REVEL < 0.5 were linked to the phenotype term HP:0002180 (neurodegeneration) (Supplementary Data 2).

Overall, this analysis suggests that the parental WIBR3 cells do not harbor genetic variants that that would limit their use for modeling neurodegenerative diseases. This analysis is now presented in Supplemental Table 2 and discussed in the revised manuscript (Page 4-5, lines 147 – 177).

We appreciate the reviewer's suggestion to consider the COSMIC database for assessing cancer-associated mutations. While COSMIC comprehensively catalogs somatic mutations identified in cancers, it provides limited information regarding pathogenicity or functional relevance. Because our cell line collection is designed to model neurodegenerative disease, interpreting COSMIC-identified variants in this context would be challenging and unlikely to yield biologically meaningful insights. Therefore, we consider such an analysis beyond the scope of the current study.

Reviewer 1 - comment 3 (major):

The authors took great care to characterise the genomic integrity of gene-edited cell clones. However, there can be different constellations of founder mutations in clones derived from a heterogeneous starting population, and/or epigenetic variability between clones (or other factors) can alter differentiation potential in a manner not directly related to on-target or off-target mutations. How do the authors plan to account for these effects to ensure that they do not potentially mask the effects of the engineered mutations they wish to study? Some consortia such as iNDI have addressed this issue by correcting the mutant allele back to a wild-type allele in 'revertant' clones. Do the authors have any plans to generate such clones or use alternative approaches to increase the value of their resource? If not, why?

Response:

We fully agree with the reviewer that distinguishing engineered mutation–specific phenotypes from those arising due to genetic or non-genetic variation, independent of the engineered genotype,

remains one of the major challenges in genetic engineering and pluripotent stem cell–based disease modeling. As the reviewer noted, reverting genome-edited cell lines to a wild-type genotype has been suggested as one approach to help separate mutation-specific effects from alterations caused by unintended variation.

However, we believe this strategy has limitations. Our analysis shows that hPSCs acquire ~20 additional mutations with each passage, which would be further propagated and fixed during any additional clonal reversion step. Such changes could themselves influence phenotypic outcomes, complicating interpretation. Moreover, even when revertant lines are generated, multiple independently targeted clonal lines would still be required to account for de novo variation introduced during culture. While we think that an extensive comparison of different approaches is beyond the scope of this work, we briefly addressed this issue in the Discussion (Page 14, lines 527 – 535). As outlined in our manuscript, we therefore advocate analyzing multiple independently gene-edited clones for each genotype, combined with comprehensive quality control, to robustly account for this variability.

A central goal of the iSCORE-PD collection is to establish a unified genetic interaction map to understand how high-risk PD-associated mutations, and their phenotypes interact to drive pathology. Given the genetic drift and accumulation of genetic and epigenetic alterations during culture, using multiple independent clones is essential when comparing phenotypes across different disease-causing mutations. For these reasons, we currently do not generate revertant (mutation-corrected) lines as part of the iSCORE-PD collection, since doing so across multiple clones per genotype is beyond the practical scope of this resource. Nevertheless, we recognize the value of revertant lines for addressing specific mechanistic questions and have established genome-editing pipelines to support this approach if needed in the future.

<u>Reviewer 1 - comment 4 (major):</u>

Validation that at least one of the gene-edited clones recapitulates PD-relevant phenotypes known to exist in iPSC-derived cellular models would increase reader confidence in the utility of the resource.

<u>Response:</u>

The main focus of this work is to establish a rigorously quality-controlled collection of human stem cell lines genetically engineered to harbor high-risk or causal variants in genes associated with PD. This resource is intended to support the scientific community in dissecting the pathology of PD. Given the scope and scale of this unique collection, we consider in-depth functional characterizations beyond the scope of the current manuscript. However, in parallel work, we have analyzed global gene expression and pre-mRNA splicing patterns in *in vitro* differentiated, midbrain-specific dopaminergic neurons carrying pathogenic PD mutations in *PRKN, SNCA, LRRK2, PINK1, DNAJC6, FBXO7, SYNJ1, DJ1, VPS13C, ATP13A2,* and *GBA1*. These analyses revealed mutation-associated splicing alterations overlapping with those observed in postmortem PD brains (Lee et al., 2025, bioRxiv: https://doi.org/10.1101/2024.02.28.582420). In addition, we used *in vitro* differentiated midbrain dopaminergic neurons carrying the *LRRK2* G2019S mutation to corroborate findings from PD patient brain

samples showing that TDP-43 loss of function drives aberrant splicing in PD (Brenton et al., 2025; bioRxiv: https://doi.org/10.1101/2025.09.04.673943). This information, including references has been added to the revised manuscript (Page 15, lines 576–583).

Reviewer 1 - comment 5 (major):

It is not totally clear if all gene editing experiments used the WIBR3 cell line or a subclone of WIBR3, given the deep characterisation of sub-clones and the narrative flow. If a non-subcloned culture of WIBR3 formed the basis of gene editing experiments, what's the rationale for that choice given that subclones had been previously generated and functionally validated? We agree with the authors' interpretation that shared variants among gene-edited clones were likely due to founder effects. Depending on the degree of the heterogeneity present in the WIBR3 culture, it difficult to accurately assess the 'mutation rate' that the authors propose, since unique variants present at low fraction among the starting population can become fixed during cloning. To assess mutation rate accurately, many clonal lines would have to be generated and propagated from a sub-clone, not the parental WIBR3 line. These laborious studies seem beyond the scope of the study, so it seems most appropriate to tone down the strength of statements to those that are clearly supported, and to clearly articulate potential confounds to the reader. The value of the manuscript mainly derives from the resource and the careful QC done on clones, and the section on genetic analysis could be shortened and clarified without loss of impact.

Response:

While the reviewer is correct that we could have used a subcloned WIBR3 line as the starting point for our gene-targeting experiments, we instead chose the earliest available passage of the parental WIBR3 hESC line. Our reasoning was twofold: (1) as our analysis shows, any additional subcloning step carries the risk of introducing additional founder mutations through cell culture, and (2) the expansion and banking required to generate sufficient cells from a subclone for a cell collection at this scale would require multiple passages, thereby reintroducing culture-induced variation comparable to that of using the non-subcloned early-passage parental line. To minimize genetic drift and reduce the genetic distance to the originally derived parental line, we prioritized using the earliest available passage of WIBR3.

We agree that not starting from a single-cell-derived clonal line complicates the precise estimation of the mutation rate. However, given the large number of clonal cell lines analyzed, we believe our dataset provides sufficient statistical power to estimate a rate of ~20 mutations per passage (17.3; 95% CI: 11.52 to 23.08, Figure 4D). To further address this point, we analyzed homozygous DJ1/PARK7 clones, which, consistent with the reviewer's suggestion, were generated through successive targeting from a heterozygous clonal line. Although limited in number, these clones accumulated an average of 21.6 mutations per passage (~151 mutations over 7 passages) between the two clonal targeting steps, supporting our estimates of acquired mutations resulting from standard cell culture. We have revised the text to clarify that our reported value represents an estimate (Page 10, line 372) and to include this additional analysis (Page 11, lines 407–408). Importantly, while the exact mutation rate is not be central to the manuscript, the key conclusion remains, as the reviewer notes, that the majority of genetic variation observed in gene-edited hPSCs arises from preexisting variants in the parental founder line acquired during

routine culture, rather than from CRISPR-mediated editing itself. We view this as an important finding, as it provides a practical roadmap for generating such collections and managing variation through rigorous quality control and careful experimental design, as discussed in detail in the discussion of our manuscript.

Reviewer 1 - comment 6 (minor):

Though data from the differentiation WIBR3 subclones S1-3 is shown (Fig. S3) and suggest some variability in the production of clusters 3 and 4, these data are not quantified. Similarly, microglial production experiments from sub-clones rely on a few markers and no quantification is shown.

Response:

We thank the reviewer for this helpful comment. In response, we have included the quantification of both the proportion and number of cells within each cluster following the differentiation into dopaminergic neurons for all WIBR3 subclones in Supplemental Figure 3A.

Regarding microglia, we used a previously described protocol to validate the efficient and robust differentiation of the parental WIBR3 and its subclones (WIBR3-S1, WIBR3-S2, WIBR3-S3) into microglia-like cells (iMGs). Following the reviewer's suggestion, we have now incorporated FACS-based quantification of microglial differentiation for each subclone, demonstrating that a high percentage of cells express the key mature microglial markers CX3CR1 and P2RY12 (>90% CX3CR1+/P2RY12+). These new results indicate that all WIBR3 subclones efficiently and robustly generate mature iMGs are presented in Figure 2H–J, Supplementary Figure 5A and discussed in the revised manuscript (Page 7, lines 242-248).

Reviewer 1 - comment 7 (minor):

The authors state that there is a high level of genomic instability (Page 13), which could be misleading to the reader, since previous studies suggest that other cell lines accumulate mutations in culture and upon gene editing at similar or higher rates than hPSCs (10.1038/s41467-020-15271-3).

Response:

We thank the reviewer for this comment, and to prevent any potential misunderstanding, we have removed the sentence from the discussion in the revised manuscript.

Reviewer 1 - comment 8 (minor):

Having line numbers in the manuscript would have helped point out specific comments in the text.

Response:

We have included line numbers in the revised manuscript.

Reviewer 1 - comment 9 (minor):

Other groups have shown that "perfect isogenic clones do not exist in vitro", and have argued for using recently derived sub-clones to reduce sequence heterogeneity and resulting founder effects. While these are important confirmation by the authors, it's over-reaching to declare them as novel Major Findings.

Response:

While the concepts of genetic drift during cell culture, founder effects introduced during subcloning, and CRISPR/Cas9-induced off-target modifications are widely accepted and supported by some experimental evidence, the extent to which each of this processes contributes to genetic variability of genome edited hPSC lines, and whether this variability follows a random or systematic distribution pattern, has not yet been thoroughly explored in sufficiently powered larger-scale sample sets. However, following the reviewer's suggestion, we have changed the wording from "major" to "main" findings in the revised manuscript (Page 14, line 513).

Reviewer 1 - comment 10 (minor):

Readers might benefit from a brief clarification of the clinical relevance of each type of introduced mutation, perhaps summarised in tabular form in a main figure (e.g. extension of Fig 3C or Fig. 4C), to provide an overview of the resource.

Response:

We thank the reviewer for this comment. Following the reviewer's suggestion, we have now included additional information (frequency, proposed disease mechanism and additional clinical information/notes in Table 1). The detailed description of all mutations including the most relevant citations can be found in the Supplementary Note 1 in the Supplementary Information.

Reviewer 1 - comment 11 (minor):

How do the authors plan to ensure that gene-edited cell clones show similar differentiation potential as the parental cell line or sub-clones? It's not reasonable to request that the authors do this for all clones, but articulating the strategy by which they will provide evidence of validation to the broader community would be helpful.

Response:

As mentioned above, the main focus of this work is to establish a rigorously quality-controlled collection of human stem cell lines genetically engineered to harbor high-risk or causal variants in genes associated with PD. This resource is intended to support the scientific community in dissecting the pathology of PD. Given the scope and scale of this unique collection, we believe that in-depth functional analyses are beyond the scope of the current manuscript. However, in parallel work, we have analyzed global gene expression and pre-mRNA splicing patterns in *in vitro* differentiated, midbrain-specific dopaminergic neurons carrying pathogenic PD mutations in *PRKN, SNCA, LRRK2, PINK1, DNAJC6, FBXO7,*

SYNJ1, DJ1, VPS13C, ATP13A2, and *GBA1*, indicating the efficient and robust in vitro differentiation of all cell lines. These analyses revealed mutation-associated splicing alterations overlapping with those observed in postmortem PD brains (Lee et al., 2025, bioRxiv: https://doi.org/10.1101/2024.02.28.582420). In addition, we used *in vitro* differentiated midbrain dopaminergic neurons carrying the *LRRK2* G2019S mutation to corroborate findings from PD patient brain samples showing that TDP-43 loss of function drives aberrant splicing in PD (Brenton et al., 2025; bioRxiv: https://doi.org/10.1101/2025.09.04.673943). This information, including references has been added to the revised manuscript (Page 15, lines 576–583).

Reviewer 1 - comment 12 (minor):

What are the plans to extend the iSCORE-PD resource to other genetic backgrounds, or to model additional PD-associated genetic variants?

Response:

In this work, we established a high-throughput gene-editing pipeline that can be easily applied to other cell lines and enables the rapid and consistent generation of new mutations using the same rigorous characterization workflow described in this study. As noted in the discussion section of our manuscript (Page 15, lines 588–590), we hope, with input and collaboration from other laboratories, to expand the iSCORE-PD collection to include additional PD-associated variants, both common and rare, as well as to introduce mutations into diverse genetic backgrounds to address variability across different populations.

Reviewer #1 (Remarks on code availability):

I do not have the necessary expertise to review the code.

Reviewer #3

Reviewer 3 - comment 1:

Since the cell collection was derived from female embryonic stem cells, the authors should consider the phenomenon of X-chromosome erosion (Kilens et al., 2018, DOI: 10.1038/s41467-017-02107-w; Vallot et al., 2015, DOI: 10.1016/j.stem.2015.03.016). During in vitro culturing of female cells, partial or complete reactivation of the inactive X-chromosome frequently occurs, which may affect cell proliferation and differentiation. This phenomenon is important to account for, as it could play a key role in the development of pathogenic phenotypes in PD modeling in vitro. Authors should discuss this aspect in the article.

Response:

We thank the reviewer for his comment, and we totally agree that this is an important concern of using female human embryonic stem cells. We have therefore included the following paragraph in the discussion of our revised manuscript (Page 15, lines 558-565):

"An additional, well-established concern in female hPSC lines is the phenomenon of X-chromosome erosion which can emerge over time in culture and lead to aberrant expression of X-linked genes[90-92], thereby altering cellular phenotypes and confounding the interpretation of experimental outcomes. Monitoring X-inactivation status therefore is essential to ensure reproducibility and accurate data interpretation. Because clonally derived cell lines remain susceptible to cell culture-induced genetic drift as well as genetic and epigenetic alterations including X-chromosome erosion, we advocate using multiple independently gene-edited clonal lines for each genotype to account for this variability."

Reviewer 3 - comment 2:

The study also has some limitations. For example, in describing the hESC culture conditions, the authors stated "95% CO_2 and 5% O_2" which is incorrect. Furthermore, the authors did not demonstrate the ability of the edited hESCs to differentiate into dopaminergic neurons. This ability should be shown at least on several lines. This aspect is important when modeling PD.

Response:

We thank the reviewer for pointing this out. We have corrected this typo in the revised manuscript which now reads "… in a cell culture incubator under low oxygen conditions (5% CO2, 5% O2) …" (Page 16, lines 608 and lines 614).

As already described in detail in response to reviewer 1's comments, the main focus of this work is to establish a rigorously quality-controlled collection of human stem cell lines genetically engineered to harbor high-risk or causal variants in genes associated with PD. This resource is intended to support the scientific community in dissecting the pathology of PD. Given the scope and scale of this unique collection, we consider in-depth functional characterizations beyond the scope of the current manuscript. However, in parallel work, we have analyzed global gene expression and pre-mRNA splicing patterns in *in vitro* differentiated, midbrain-specific dopaminergic neurons carrying pathogenic PD mutations in *PRKN, SNCA, LRRK2, PINK1, DNAJC6, FBXO7, SYNJ1, DJ1, VPS13C, ATP13A2,* and *GBA1*. This analysis revealed that all included cell lines efficiently and consistently generate dopaminergic neurons. Furthermore, we identified mutation-associated splicing alterations overlapping with those observed in postmortem PD brains (Lee et al., 2025, bioRxiv: https://doi.org/10.1101/2024.02.28.582420). In addition, we used *in vitro* differentiated midbrain dopaminergic neurons carrying the *LRRK2* G2019S mutation to corroborate findings from PD patient brain samples showing that TDP-43 loss of function drives aberrant splicing in PD (Brenton et al., 2025; bioRxiv: https://doi.org/10.1101/2025.09.04.673943). This information, including references has been added to the revised manuscript (Page 15, lines 576–583).

Reviewer #3 (Remarks on code availability):

The results of the work are reproducible and the code is a usable resource for the community. The README file contains sufficient instructions to install and run the application.

Reviewer #4

Reviewer 4 - comment 1:

The detailed genetic analysis presented in this study supports the validity of the genome-edited lines and the parental WIBR3 cells as a hereditary Parkinson's disease model and its corresponding control. However, considering the broader context of reference cell utility in stem cell research (DOI: 10.1016/j.stem.2025.05.004), the justification for selecting WIBR3 as the parental cell line appears somewhat weak. If there is a specific reason for selecting WIBR3 over other available cell lines, please provide an explanation.

Response:

We thank the reviewer for pointing us to a recent publication discussing the concept of reference iPSC lines. We agree with the authors of this article that the characteristics of reference lines may vary depending on the intended applications, and that there is an important conceptual distinction between a “control” and a “reference” cell line. Given the genetic variability in the human population, with more than 3 million SNVs, including potentially pathogenic variants, present in any individual, the authors suggest that no universal line is suitable for the study of all tissue types. Even KOLF2.1J, which is currently widely recognized as a benchmark reference for neurodegenerative disease research, has limitations as it was primarily selected for efficient gene editing, raising the possibility that genetic or epigenetic modifications are present in this cell line that contributing to higher editing efficiency.

As outlined in our manuscript (Page 3, lines 103–110), our rationale for choosing the female hESC line WIBR3 was: (1) concerns regarding somatic mutations in donors prior to reprogramming, as well as reprogramming-induced genetic alterations, incomplete epigenetic reprogramming, and aberrant genomic imprinting in iPSCs; (2) the importance of including cell lines from both sexes, as male iPSCs are often favored due to concerns about X-chromosome erosion; (3) the fact that WIBR3 has been shown to maintain a stable karyotype over extended culture; (4) WIBR3 hESCs have been previously widely used in modeling human diseases, including PD and (5) access to cells at an early-passage after derivation (P14).

In line with the authors’ argument, our main goal was to provide additional diverse cell lines that could be compared with existing reference lines. While we agree that an hESC line cannot formally fulfill the definition of a ‘reference’ line, since it is impossible to predict whether WIBR3 represents a healthy individual, the same argument applies to human iPSCs as it is hard to predict whether an individual would have develop a neurodegenerative disease in the future. Importantly, the here described quality control

and characterization of WIBR3 follows the standards expected of reference lines currently in use. Our in-depth functional and genetic analyses further suggest that WIBR3 does not harbor functional limitations or variants that would substantially restrict its utility for modeling neurodegenerative disease.

Nevertheless, in response to the reviewer's concern, we have revised wording referring to WIBR3 as a "reference" cell line, acknowledging that hESCs cannot formally be a reference lines, as their donor health status cannot be fully predicted. The revised discussion (Page 13, lines 493-495) now states: "Thus, WIBR3 cells are a highly characterized female hESC line, providing a valuable complement to existing hPSC lines for modeling neurodegenerative diseases."

Reviewer 4 - comment 2:

Related to the previous question, would it be feasible to evaluate the similarity between the WIBR3 cells and KOLF2.1J cells, which are currently considered a reference cell line, in terms of genomic sequence, gene expression, and differentiation efficiency? Given the differences in donor origin and the inherent distinctions between iPSCs and ESCs, I understand that such a comparison may be challenging. However, conducting this analysis could further enhance the value of the generated cell library.

Response:

As outlined in our response to Reviewer 1, we have substantially strengthened our genomic analyses to exclude the presence of SNVs and CNVs that could be pathogenic in the context of modeling neurodegenerative diseases (Page 4-5, lines 147 – 177). These analyses indicate that the parental WIBR3 cells do not harbor variants that would significantly limit their utility for modeling of neurodegenerative diseases. As comparable information is available for KOLF2.1J and other reference cell lines, this new analysis will help investigators to assess whether this cell line is suitable for their specific intended applications.

Given the genetic variability in the human population, with over 3 million SNVs, including potentially pathogenic variants, present in any individual, a genetic comparison between the two lines, while technically feasible, would be primarily descriptive and unlikely to provide meaningful biological or functional insights. Moreover, given the widely recognized functional variability among hPSC lines, we believe that functional and/or gene expression comparisons are informative only when conducted across a large and genetically diverse set of lines, rather than between just two.

As noted in our earlier response, we consider the parental WIBR3 line and its clonal derivatives (WIBR-S1–S3, EWT1–8) to serve as experimental control for the genetically modified iSCORE-PD lines rather than a true reference line, a concept that is itself debatable. Nonetheless, the WIBR3 cells described here have undergone rigorous quality control to the standards expected of any reference line, making them well suited for inclusion in larger-scale functional comparisons as discussed above.

Reviewer 4 - comment 3:

In Figure 4D, overlapping dots of similar colors make it difficult to distinguish individual data points. To

effectively support the claims presented in this figure, rather than displaying a single shared trend line, it would be necessary to illustrate that EWT_S1_8 and the other genome-edited lines exhibit similar tendencies.

Response:

We thank the reviewer for pointing this out. We have modified Figure 4D accordingly in the revised manuscript.

Reviewer #5:

I co-reviewed this manuscript with one of the reviewers who provided the listed reports. This is part of the Nature Communications initiative to facilitate training in peer review and to provide appropriate recognition for Early Career Researchers who co-review manuscripts.

Dear Reviewers,

Thank you very much for your thoughtful assessment of our revised manuscript, "iSCORE-PD: an isogenic stem cell collection to research Parkinson's Disease." We are grateful for the constructive feedback, and we appreciate the opportunity to submit an additional revision. We are pleased that all reviewers—except Reviewer 1—were fully satisfied with our previous responses.

While Reviewer 1 was satisfied with most of our revisions, their remaining concern relates to demonstrating the utility of the iSCORE-PD collection for modelling Parkinson's disease by recapitulating phenotypes previously described in the literature. We would like to clarify our perspective on this important point.

As we understand it, the reviewer's concern has two components:

(*i*) providing evidence, that gene-edited cell lines efficiently can generate dopaminergic neurons and

(*ii*) demonstrating the iSCORE-PD collection can model Parkinson's disease by recapitulating phenotypes previously described in the literature.

With respect to point (*i*), as indicated in our previously revised manuscript (Page 15, lines 581–585), our collaborators have already differentiated the majority of our cell lines, including multiple clones for PRKN, SNCA, LRRK2, PINK1, DNAJC6, FBXO7, SYNJ1, DJ1, VPS13C, ATP13A2, and GBA1 into midbrain-specific dopaminergic neurons. The analysis of global gene expression and pre-mRNA splicing patterns in these in vitro–differentiated neurons carrying pathogenic PD mutations revealed mutation-associated splicing alterations that overlap with changes observed in postmortem PD patient brain tissue (Lee et al., 2025; https://doi.org/10.1101/2024.02.28.582420). In a second study, another collaborator used midbrain-specific dopaminergic neurons carrying the *LRRK2 G2019S* mutation to corroborate findings from PD patient samples showing that TDP-43 loss of function drives aberrant splicing in PD (Brenton et al., 2025; https://doi.org/10.1101/2025.09.04.673943). Together, these independent studies demonstrate (*1*) that all tested cell lines efficiently differentiate into midbrain-specific dopaminergic neurons, and (*2*) that *in vitro* phenotypes observed in these cell lines are recapitulated in patient brain tissue, thereby clearly supporting the utility and scientific relevance of the iSCORE-PD collection.

It is important to note that these studies were led by independent research groups. Because our funding agency ASAP mandates rapid dissemination of reagents through preprints and early distribution across the ASAP Collaborative Research Network, which includes more than 200 Parkinson's research laboratories, we made these lines broadly available from an early stage. As these experiments are being conducted independently, it is not feasible at present to incorporate data from these ongoing external studies directly into our manuscript. Nevertheless, we believe that these results strongly support the usefulness and scientific relevance of the iSCORE-PD resource for the broader research community.

Regarding point (*ii*), there are currently only a few universally accepted "gold-standard" PD phenotypes in iPSC-derived neuronal systems, and no standardized differentiation protocol is applied consistently across laboratories. The primary motivation behind iSCORE-PD, and similar initiatives such as

the iNDI (iPSC Neurodegenerative Disease Initiative), is precisely to address this challenge by providing genetically controlled, standardized starting materials and harmonized procedures that enable meaningful comparisons across studies, experiments, and institutions.

Our review of the PD iPSC literature revealed that very few cellular phenotypes are reproducibly observed across laboratories. Published findings often rely on small sample sizes, non-isogenic controls, or laboratory-specific differentiation protocols. As a result, many phenotypes remain controversial or challenging to reproduce. Under these circumstances, repeating a small selection of PD phenotypes in our system would not necessarily strengthen the manuscript nor provide novel insight into the pathogenesis of PD. Instead, systematic and unbiased cross-line comparisons, already underway in related work by collaborating groups, are better positioned to identify robust and reproducible PD-associated biology across genotypes.

A further practical consideration is that many reported PD phenotypes require complex, custom, and highly specialized differentiation protocols, often taking months to establish, along with technically demanding assays that are not standardized across laboratories. Because these phenotypes can be strongly influenced by genetic background, failure to recapitulate a published phenotype would require additional work, potentially including reanalysis of the original donor line, to distinguish technical variation from biological variability or genetic modifiers. It is therefore not clear which specific experiments would be most scientifically meaningful to include. Given these conceptual and practical limitations, we believe that such efforts fall outside the scope of this resource-focused manuscript and would not provide new mechanistic insight into PD pathogenesis.

Moreover, while this manuscript primarily focuses on the generation and validation of the iSCORE-PD resource, an equally important scientific objective of this work is to better understand the sources of genetic variation that inevitably arise in stem cell–based systems, and to provide a roadmap for establishing comparable collections and managing such variation through stringent quality control and thoughtful experimental design.

Finally, we have incorporated Reviewer 1's suggested revisions in the revised discussion as outlined in our point-by-point response. All corresponding revisions are highlighted in blue in the revised manuscript.

REVIEWER COMMENTS

Reviewer #1

<u>Reviewer 1 - comment 1:</u>

If this is to be a stand-alone manuscript describing the generation of a resource meant to be of value to the community seeking to model PD, it seems critical to demonstrate that the resource can indeed usefully model PD. The authors state that in-depth characterisation of the resource is outside of the scope of this study and instead offer some analysis in separate pre-prints. While in-depth characterisation of all of the mutant lines would indeed be excessive, it seems reasonable to either include some of the work

cited in the preprints in this manuscript, or to include a small handful of additional examples to show that the resource is indeed useful for modelling PD by recapitulating phenotypes previously shown in the literature.

Response:

The reviewer's concern relates to demonstrating the utility of the iSCORE-PD collection for modelling Parkinson's disease by recapitulating phenotypes previously described in the literature. As already outlined above, we would like to clarify our perspective on this important point. As we understand it, the reviewer's concern has two components:

(*i*) providing evidence, that all gene-edited cell lines efficiently can generate dopaminergic neurons and

(*ii*) demonstrating the iSCORE-PD collection can model Parkinson's disease by recapitulating phenotypes previously described in the literature.

With respect to point (*i*), as indicated in our previously revised manuscript (Page 15, lines 581–585), our collaborators have already differentiated the majority of our cell lines, including multiple clones for PRKN, SNCA, LRRK2, PINK1, DNAJC6, FBXO7, SYNJ1, DJ1, VPS13C, ATP13A2, and GBA1 into midbrain-specific dopaminergic neurons. The analysis of global gene expression and pre-mRNA splicing patterns in these in vitro–differentiated neurons carrying pathogenic PD mutations revealed mutation-associated splicing alterations that overlap with changes observed in postmortem PD patient brain tissue (Lee et al., 2025; https://doi.org/10.1101/2024.02.28.582420). In a second study, another collaborator used midbrain-specific dopaminergic neurons carrying the *LRRK2 G2019S* mutation to corroborate findings from PD patient samples showing that TDP-43 loss of function drives aberrant splicing in PD (Brenton et al., 2025; https://doi.org/10.1101/2025.09.04.673943). Together, these independent studies demonstrate (*1*) that all tested cell lines efficiently differentiate into midbrain-specific dopaminergic neurons, and (*2*) that *in vitro* phenotypes observed in these cell lines are recapitulated in patient brain tissue, thereby clearly supporting the utility and scientific relevance of the iSCORE-PD collection.

It is important to note that these studies were led by independent research groups. Because our funding agency ASAP mandates rapid dissemination of reagents through preprints and early distribution across the ASAP Collaborative Research Network, which includes more than 200 Parkinson's research laboratories, we made these lines broadly available from an early stage. As these experiments are being conducted independently, it is not feasible at present to incorporate data from these ongoing external studies directly into our manuscript. Nevertheless, we believe that these results strongly support the usefulness and scientific relevance of the iSCORE-PD resource for the broader research community.

Regarding point (*ii*), there are currently only a few universally accepted "gold-standard" PD phenotypes in iPSC-derived neuronal systems, and no standardized differentiation protocol is applied consistently across laboratories. The primary motivation behind iSCORE-PD, and similar initiatives such as the iNDI (iPSC Neurodegenerative Disease Initiative), is precisely to address this challenge by providing genetically controlled, standardized starting materials and harmonized procedures that enable meaningful comparisons across studies, experiments, and institutions.

Our review of the PD iPSC literature revealed that very few cellular phenotypes are reproducibly observed across laboratories. Published findings often rely on small sample sizes, non-isogenic controls, or laboratory-specific differentiation protocols. As a result, many phenotypes remain controversial or challenging to reproduce. Under these circumstances, repeating a small selection of PD phenotypes in our system would not necessarily strengthen the manuscript nor provide novel insight. Instead, systematic and unbiased cross-line comparisons, already underway in related work by collaborating groups, are better positioned to identify robust and reproducible PD-associated biology across genotypes.

A further practical consideration is that many reported PD phenotypes require complex, custom, and highly specialized differentiation protocols, often taking months to establish, along with technically demanding assays that are not standardized across laboratories. Because these phenotypes can be strongly influenced by genetic background, failure to recapitulate a published phenotype would require additional work, potentially including reanalysis of the original donor line, to distinguish technical variation from biological variability or genetic modifiers. It is therefore not clear which specific experiments would be most scientifically meaningful to include. Given these conceptual and practical limitations, we believe that such efforts fall outside the scope of this resource-focused manuscript and would not provide new mechanistic insight into PD pathogenesis.

Reviewer 1 - comment 2 (a):

For clarity, the inaccessibility of the resource to investigators in countries where embryonic stem cell research is not permitted would be more appropriate to include in the manuscript main text when discussing potential limitations, and not just in the Methods section.

Response:

We thank the reviewer for his comment, and we agree that this is information should be included in the main text of the manuscript. We have therefore included the following paragraph in the discussion of our revised manuscript (Page 16, lines 596-600):

”To facilitate such efforts, all generated cell lines will be made available with the support of the Aligning Science Across Parkinson's (ASAP) initiative and the Michael J. Fox Foundation (MJFF) through the WiCell Research Institute. However, we note as a limitation that the use of human embryonic stem cell–derived resources is subject to local regulatory and ethical frameworks and may therefore be restricted or not permitted in certain countries, potentially limiting accessibility for some researchers.”

Reviewer 1 - comment 2 (b):

Similarly, given that the parental cell line was not subcloned prior to gene editing, the issue of many founder mutations becoming fixed along with the intended mutation during the gene editing process, including potentially cancer-associated mutations that were not tested for directly but are known to recur during stem cell culture, should be clearly pointed out to readers as potential limitations in the Discussion.

Response:

We thank the reviewer for his comment, and we agree that this limitation should be clearly pointed out in the discussion of the manuscript. We have therefore included the following paragraph in the discussion of our revised manuscript (Page 15, lines 570-580):

"While perfect isogeneity remains elusive, the observed genetic variations were minimal compared to those typically found in classical hiPSC experiments comparing cells from patients with those from unaffected individuals. Predicting the impact of this variation on cellular and disease phenotypes is challenging. However, as the majority of these variations are random and predominantly affect non-coding regions of the genome, similar to somatic mutations found ubiquitously across normal tissues, we predict that most of the observed variation is unlikely to have a strong impact cellular fitness or disease-associated phenotypes. One limitation of our study is that we cannot entirely exclude the possibility that some of these mutations affect cellular fitness, including potentially cancer-associated mutations, which are known to occur during regular cell culture and can sometimes become fixed during sub-cloning. We have not directly tested for such mutations, as there is currently no comprehensive method to determine which specific coding or non-coding variants influence cellular fitness."
