# Supplementary material for: iSCORE-PD: an isogenic stem cell collection to research Parkinson’s disease
Source: Nat Commun. 2026 Jun 17;17:7682. doi: 10.1038/s41467-026-74355-8 (PMC13434110; doi:10.1038/s41467-026-74355-8)
Supplement: Supplementary file 3 [file 41467_2026_74355_MOESM1_ESM.pdf]

FIGURES

iSCORE-PD: an isogenic stem cell collection to research Parkinson's Disease

Oriol Busquets[1,2,3,13], Hanqin Li[3,4,5,13], Khaja Mohieddin Syed[3,4,13], Pilar Alvarez Jerez[6,7,13], Jesse Dunnack[3,4,13], Riana Lo Bu[1,3], Yogendra Verma[3,4], Gabriella R. Pangilinan[3,4], Annika Martin[4], Jannes Straub[3,4], YuXin Du[3,4], Vivien M. Simon[4], Steven Poser[1,3], Zipporiah Bush[3,8], Jessica Diaz[1,3], Atehsa Sahagun[3,4,9], Jianpu Gao[4], Samantha Hong[6], Dena G. Hernandez[10], Kristin S. Levine[6], Nathalie Pochet[1], Ezgi O. Booth[3,4], Marco Blanchette[11], Helen S. Bateup[3,4,9,12], Donald C. Rio[3,4], Cornelis Blauwendraat[6,10], Dirk Hockemeyer[3,4,5,12*], Frank Soldner [1,2,,3,8*]

[1]Dominick P. Purpura Department of Neuroscience, Albert Einstein College of Medicine, Rose F. Kennedy Center, Albert Einstein College of Medicine, 1410 Pelham Parkway South, Bronx, NY 10461, USA.

[2]Ruth L. and David S. Gottesman Institute for Stem Cell and Regenerative Medicine Research, Albert Einstein College of Medicine, 1301 Morris Park Ave., Bronx, NY 10461, USA.

[3]Aligning Science Across Parkinson's (ASAP) Collaborative Research Network, Chevy Chase, MD, 20815, USA.

[4]Department of Molecular and Cell Biology, University of California, Berkeley, Berkeley, CA 94720, USA.

[5]Innovative Genomics Institute, University of California, Berkeley, CA, 94720, USA.

[6]Center for Alzheimer's and Related Dementias, National Institute on Aging and National Institute of Neurological Disorders and Stroke, National Institutes of Health, Bethesda, MD, 20892, USA.

[7]Department of Neurodegenerative Disease, UCL Queen Square Institute of Neurology, University College London, London, UK.

[8]Department of Genetics, Albert Einstein College of Medicine, 1301 Morris Park Ave., Bronx, NY 10461, USA.

[9]Department of Neuroscience, University of California, Berkeley, Berkeley, CA 94720, USA.

[10]Laboratory of Neurogenetics, National Institute on Aging, National Institutes of Health, Bethesda, MD, 20892, USA.

[11]LiftOff Biosolution. 813 Isbel Ct, Santa Cruz 95060, USA.

[12]Chan Zuckerberg Biohub, San Francisco, CA, 94158, USA.

[13] These authors contributed equally.

[*]Corresponding authors: hockemeyer@berkeley.edu, frank.soldner@einsteinmed.edu.

Supplementary Figure 1, Busquets et al.

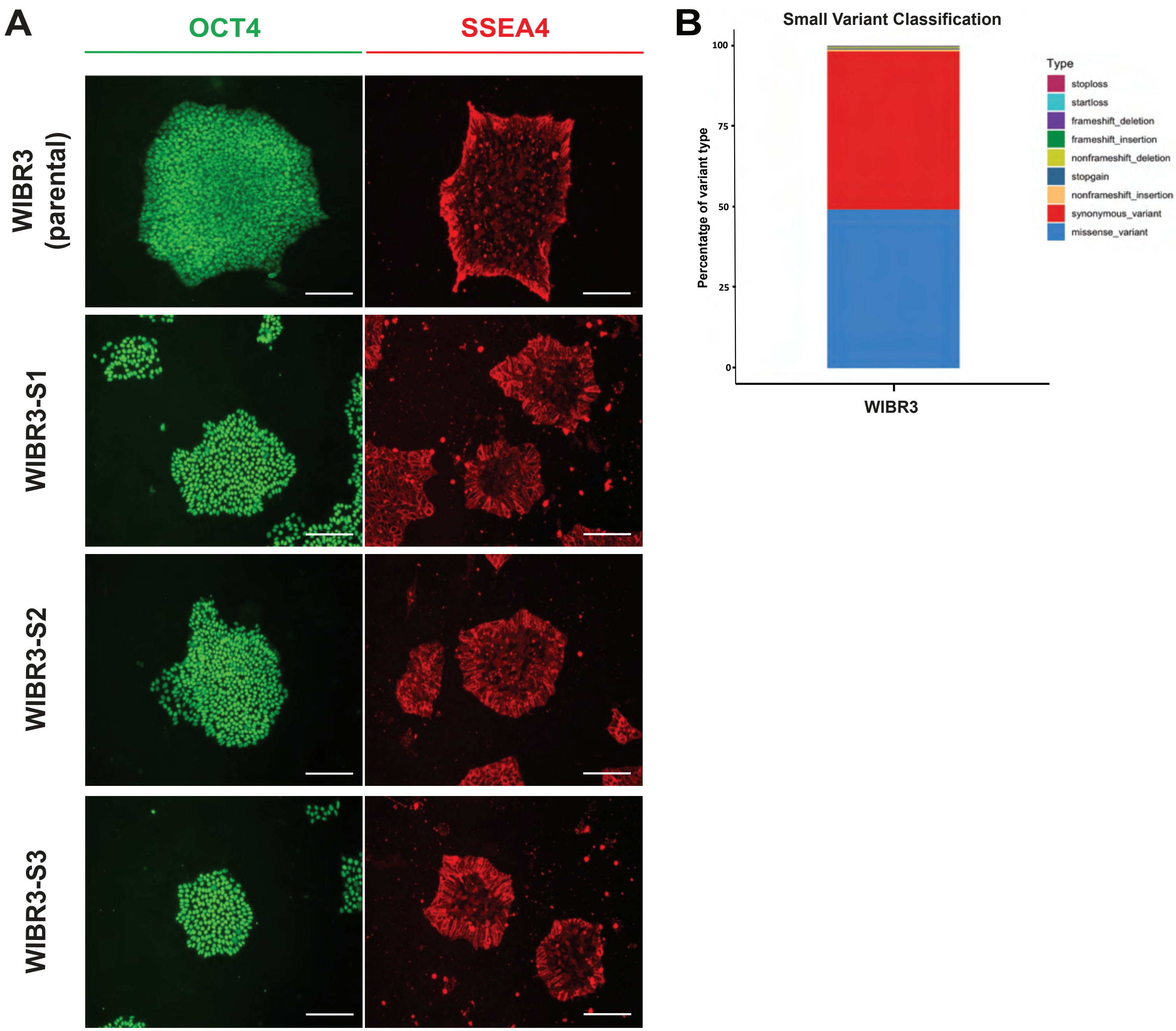

Supplementary Figure 1. Characterization of WIBR3 in feeder free conditions.

(A) Immunocytochemistry for pluripotency markers OCT4 (green) and SSEA4 (red) on hESC colonies in feeder free cultures from parental WIBR3 hESC and the clonal lines WIBR3-S1, WIBR3-S2 and WIBR3-S3. Scale bar 100 µm.

(B) The percentage of genetic variant types present in WIBR3 grouped by their predicted consequences on coding sequences.

Source data are provided as a Source Data file.

Supplementary Figure 2, Busquets et al.

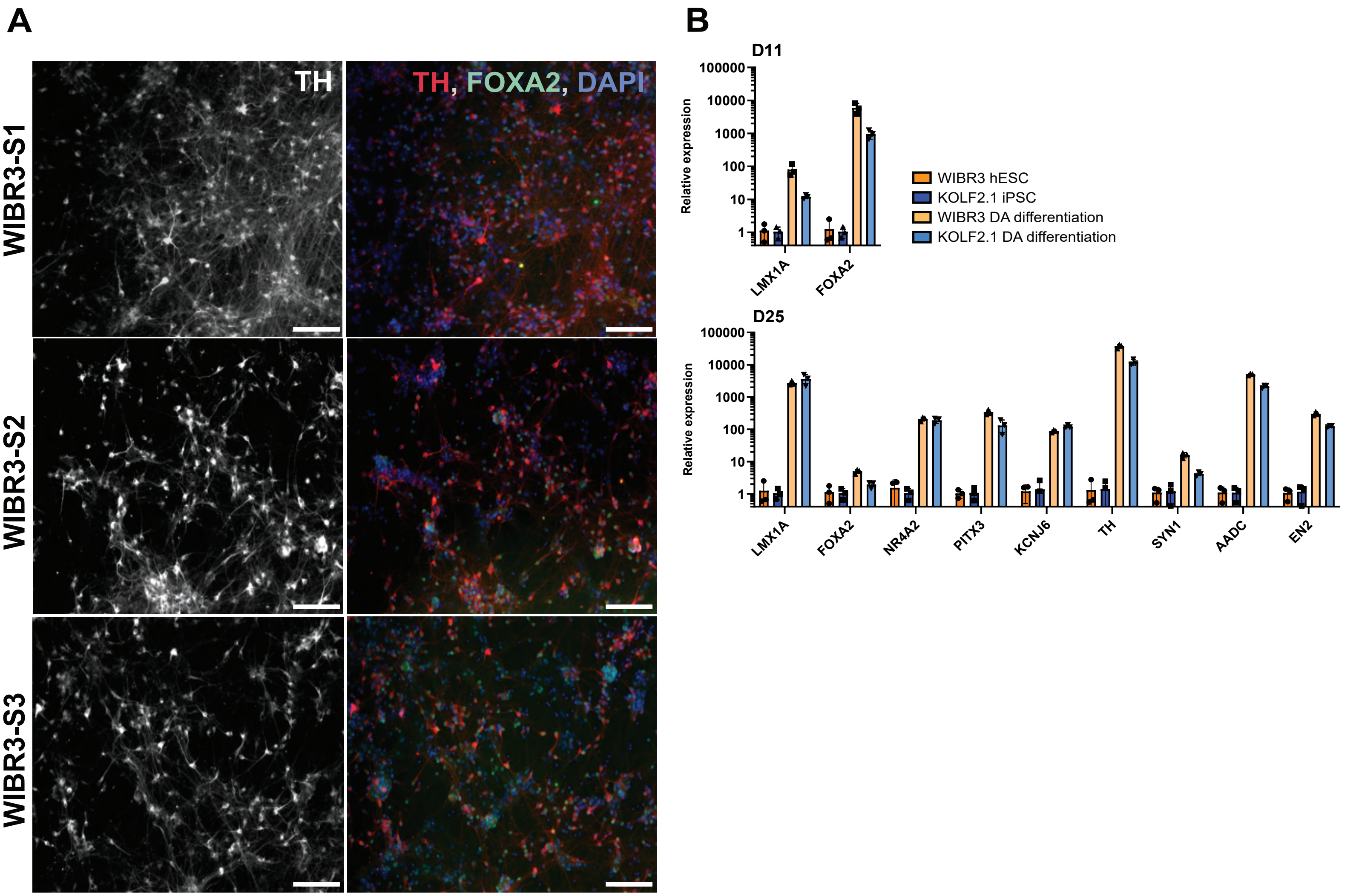

Supplementary Figure 2. Dopaminergic neuron immunostaining and gene expression analysis.
(A) Immunofluorescence of TH and FOXA2 in dopaminergic neurons (at day 35) derived from WIBR3-S1, WIBR3-S2 and WIBR3-S3, scale bar 100 µm.
(B) qRT-PCR quantification of midbrain floor plate progenitor and dopaminergic neuron markers at day 11 and day 25 of differentiation of WIBR3 and KOLF2.1 - derived cells. Relative gene expression is calculated relative to the expression of GAPDH and compared against their corresponding hPSC expression levels. (N = 3; MEAN ± SD).
Source data are provided as a Source Data file.

Supplementary Figure 3, Busquets et al.

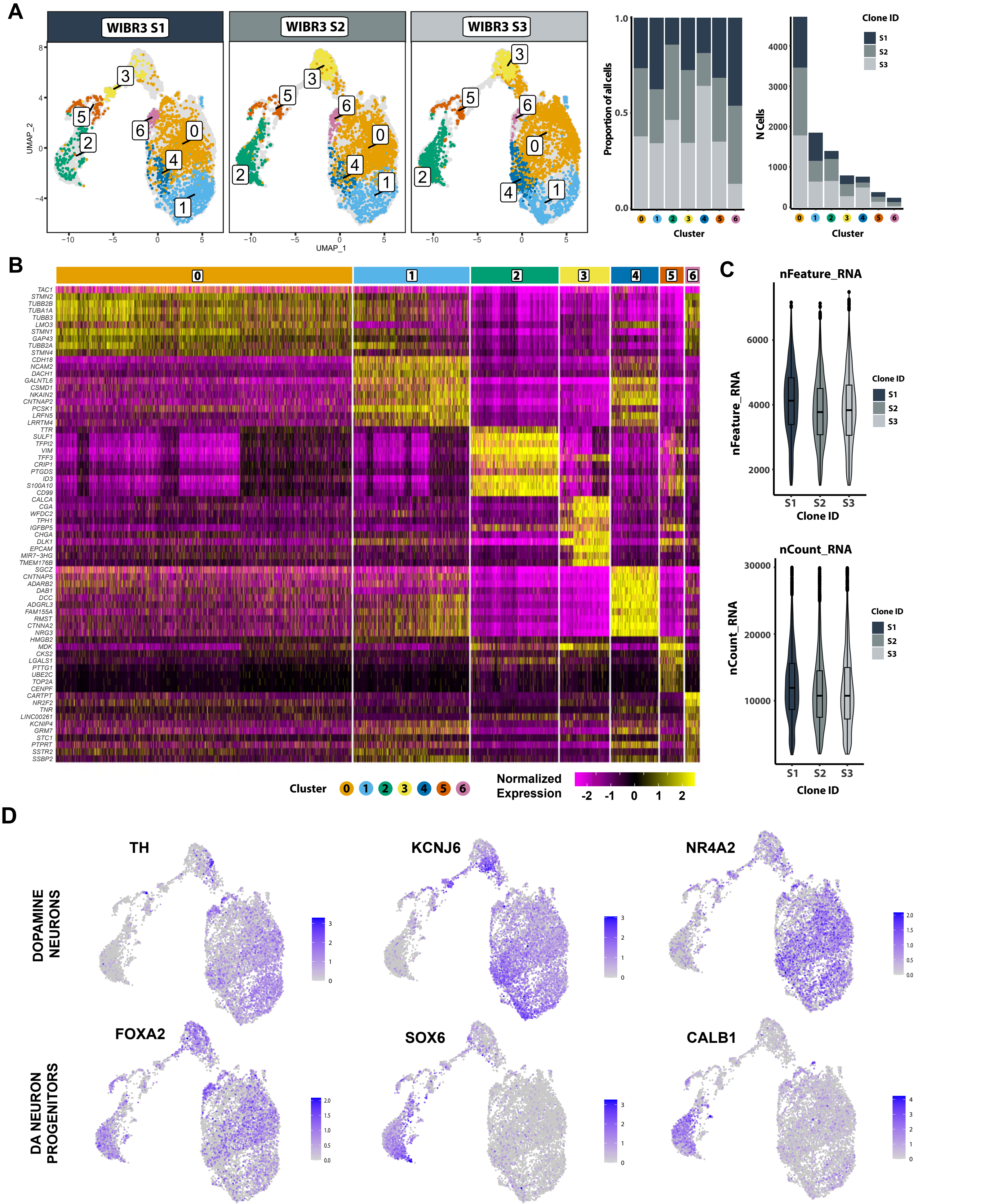

Supplementary Figure 3. Quality control metrics of single cell data.
(A) UMAPs of dopamine neurons derived from WIBR3-S1, WIBR3 -S2 and WIBR3-S3, to visualize the distribution of cells in each cluster from an integrated seurat single cells dataset.
(B) Heatmap showing top 10 genes differentially expressed in each cluster.
(C) Quality control plots of nCount RNA reads (UMIs) and feature RNA reads (genes) for each subclone. Box plots show the median (horizontal line), interquartile range (box; 25th percentile to 75th percentile), whiskers extending to values within 1.5×IQR, and outliers are shown as individual points.
(D) UMAP feature plots showing expression patterns across cell clusters of dopamine neuronal progenitor and mature dopamine neuron specific marker genes.

Supplementary Figure 4, Busquets et al.,

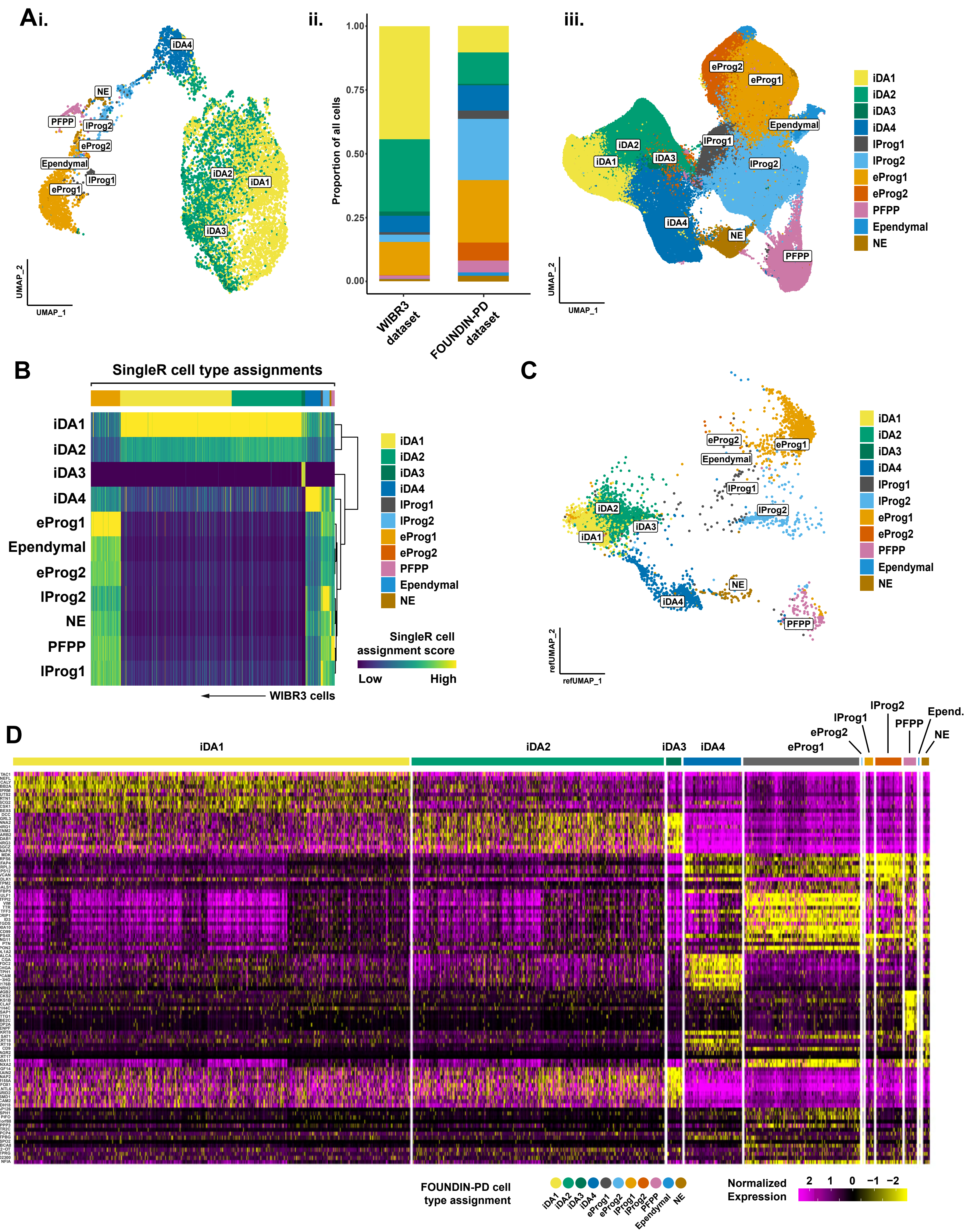

Supplementary Figure 4. scRNASeq comparison of WIBR3 cells to the FOUNDIN-PD reference.

(A) *(i)* UMAP plot of all 10,097 WIBR3 cells profiled, with labels representing a cell's assignment to a corresponding cell type as defined in the FOUNDIN-PD reference dataset. Labels for each cell are identified using the SingleR classifier in R (Wilcoxon rank sum test). *(ii)* Stacked barplot depicting the proportion of cell type identities across the entire 10,097 cell WIBR3 dataset or the 416,216 cells in the FOUNDIN-PD reference dataset. *(iii)* UMAP of the 416,216 cells in the FOUNDIN-PD reference dataset, with the author's default cell type labels applied.

(B) Heatmap depicting the cell type classification of 10,097 WIBR3 cells (rows) into a list of cognate cell type labels as defined in the FOUNDIN-PD reference dataset. Plotted values represent normalized cell type assignment scores as calculated by the SingleR package in R (Wilcoxon rank-sum test). Any cells retaining undefined identities after SingleR classification ("NA") are omitted from downstream analyses.

(C) All 10,097 WIBR3 cells depicted in the UMAP space of the FOUNDIN-PD dataset shown in panel *A iii*, with each cell depicted with a FOUNDIN-PD cell type label applied by SingleR.

(D) Heatmap of WIBR3 cells (columns) grouped by FOUNDIN-PD labels as applied by SingleR. Rows depict the top 10 marker genes that define the WIBR3 cells assigned to each FOUNDIN-PD cell type identity. Markers lists are identified with the "FindAllMarkers" function in Seurat, using the "MAST" test.

Supplementary Figure 5, Busquets et al.

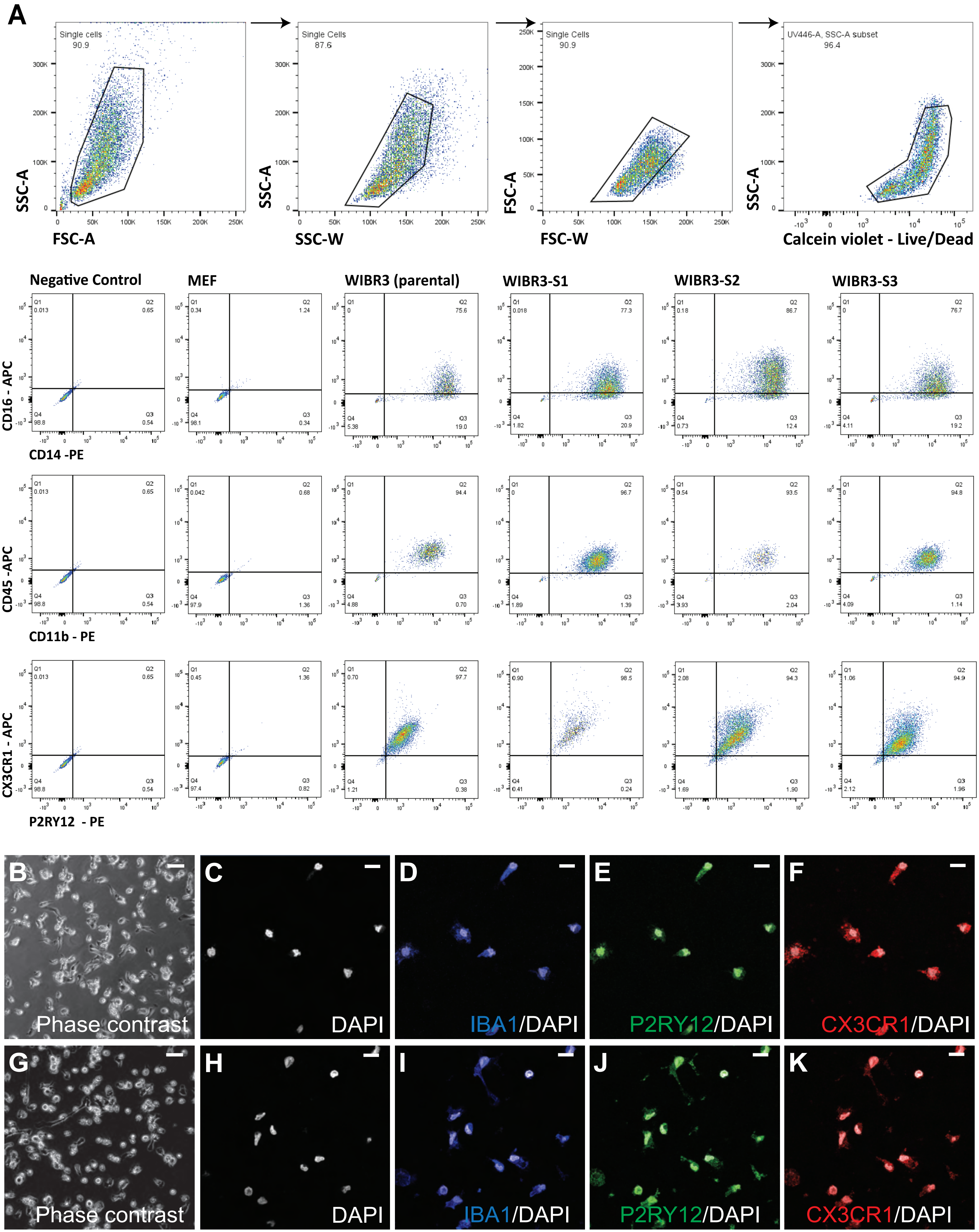

Supplemental Figure 5. ***In vitro*** **differentiation of microglia from WIBR3-S2 and WIBR3-S3**

(A) Representative image of the gating strategy used to analyze the presence of microglia lineage-specific markers (CD14/CD16, CD11b/CD45 and CX3CR1/P2RY12), and individual plots for all analyzed cell lines. Positive values were determined fr om the base value of unstained negative control sampl es and stained MEF cells who do not express the markers of interest.

(B-F) Representativ e phase contrast (B) and immunostaining (C-F) images of in vitro differentiated iMGs derived from subclone WIBR3-S2 for microglia-specific markers IBA1, P2RY12, and CX3CR1 (terminal diff day 14). Scale bar (phase contrast): 50 µm; Scale bar (ICC): 10 µm.

(G-K) Representative phase contrast (G) and immunostaining (H-K) images of in vitro differentiated iMGs derived from subclone WIBR-S3 for microglia-specific markers IBA1, P2RY12, and CX3CR1 (terminal diff day 14). Scale bar (phase contrast): 50 µm; Scale bar (ICC): 10 µm.

Source data are provided as a Source Data file.

Supplementary Figure 6, Busquets et al.

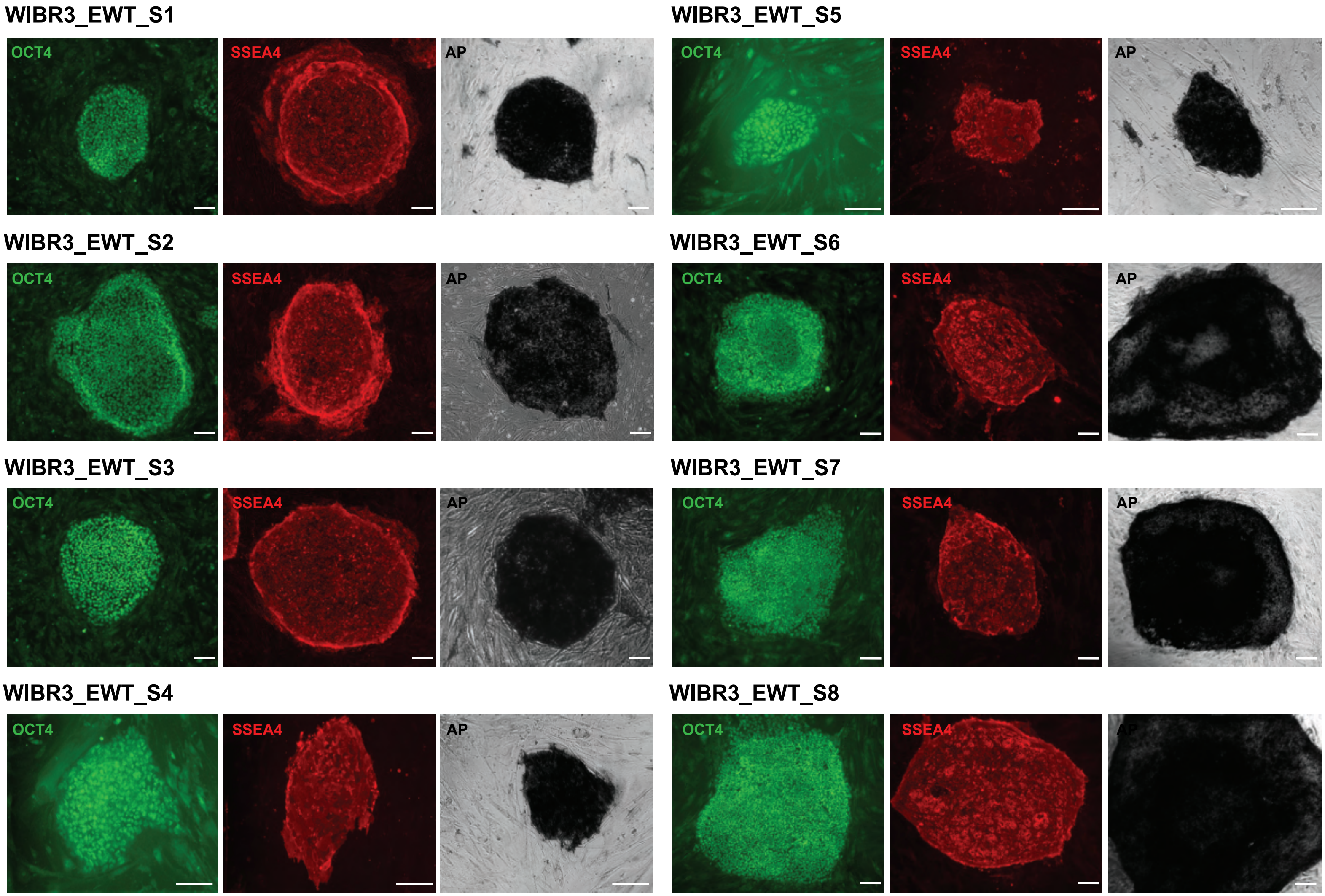

Supplementary Figure 6. Characterization of edited wild type cell lines.

Immunocytochemistry of edited wild-type (EWT) cells lines for pluripotency markers OCT4 (green), SSEA4 (red) and alkaline phosphatase (black). EWT refers to cell lines that have undergone the editing pipelines but were not genetically modified and remain genotypically wild type. WIBR3_EWT_S1-3 were isolated from a prime editing experiment using Pipeline B, WIBR3_EWT_S4-5 were isolated from CRISPR/Cas9-facilitated HDR experiments using Pipeline B and WIBR3_EWT_S6-8 were isolated from a prime editing experiment using Pipeline A. Scale bar 100 µm.

Source data are provided as a Source Data file.

Supplementary Figure 7, Busquets et al.

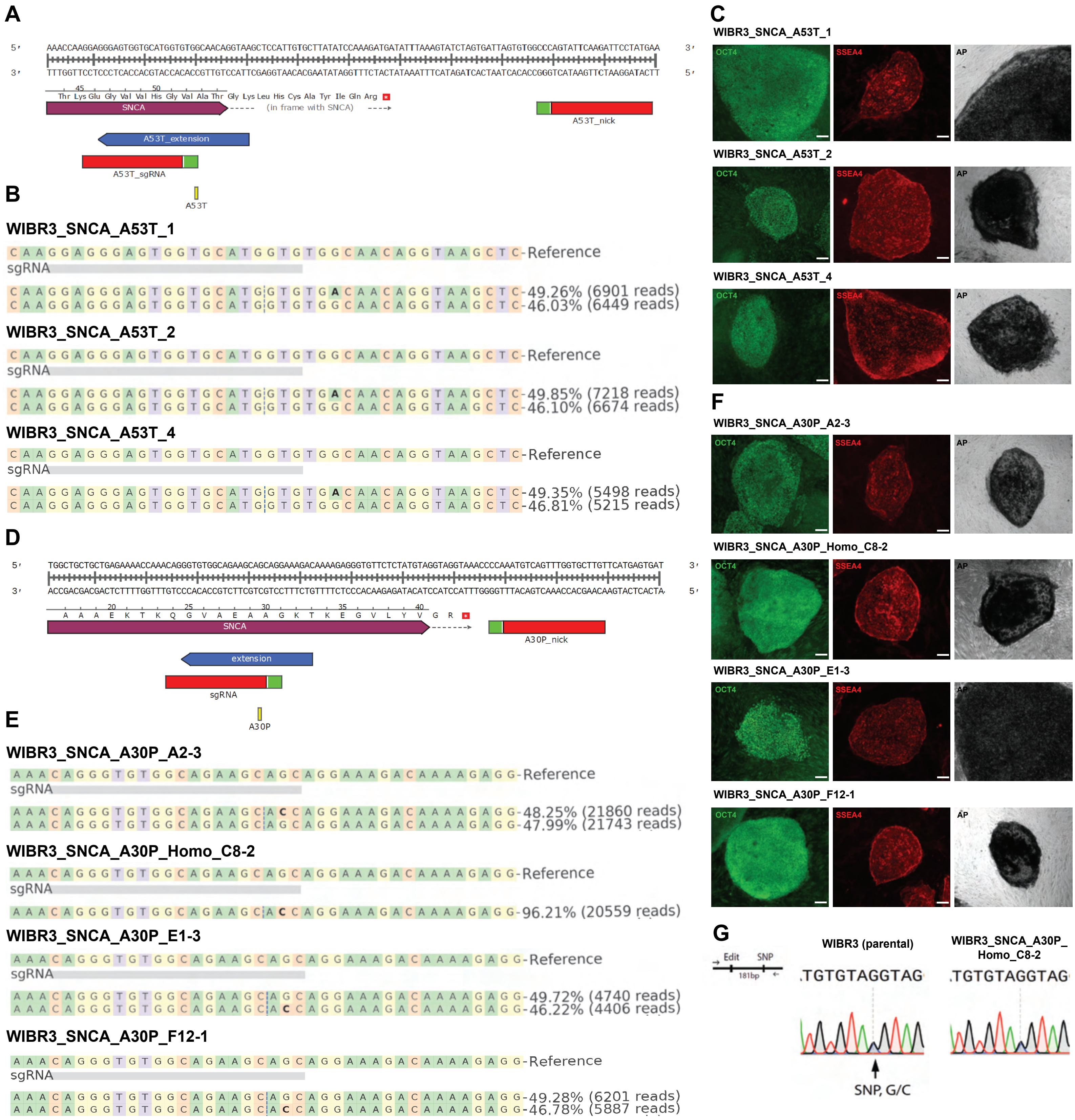

Supplemental Figure 7. Genome editing and quality control of WIBR3 hESCs carrying PD-associated mutations in *SNCA*.

(A) Targeting strategy to generate SNCA A53T mutation using prime editing.

(B) NGS-based genotyping to confirm correct editing for SNCA A53T in clones WIBR3_SNCA_A53T_1, WIBR3_SNCA_A53T_2 and WIBR3_SNCA_A53T_4. Bold bases indicate base substitutions.

(C) Immunocytochemistry of hESC cultures for pluripotency markers OCT4 (green), SSEA4 (red), and alkaline phosphatase (black). Scale bar 100 µm.

(D) Targeting strategy to generate SNCA A30P mutation by prime editing.

(E) NGS-based genotyping to confirm correct editing for SNCA A30P in clones WIBR3_SNCA_A30P_A2-3, WIBR3_SNCA_A30P_Homo_C8-2, WIBR3_SNCA_A30P_E1-3 and WIBR3_SNCA_A30P_F12-1. Bold bases indicate base substitution. The generation of the WIBR3_SNCA_A30P cell lines was previously reported[73]

(F) Immunocytochemistry of hESC cultures for pluripotency markers OCT4 (green), SSEA4 (red) and alkaline phosphatase (black). Scale bar 100 µm.

(G) Zygosity analysis using Sanger sequencing to detect heterozygous neighboring SNV to exclude LOH in clone WIBR3_SNCA_A30P_Homo_C8-2.

Source data are provided as a Source Data file.

Supplementary Figure 8, Busquets et al.

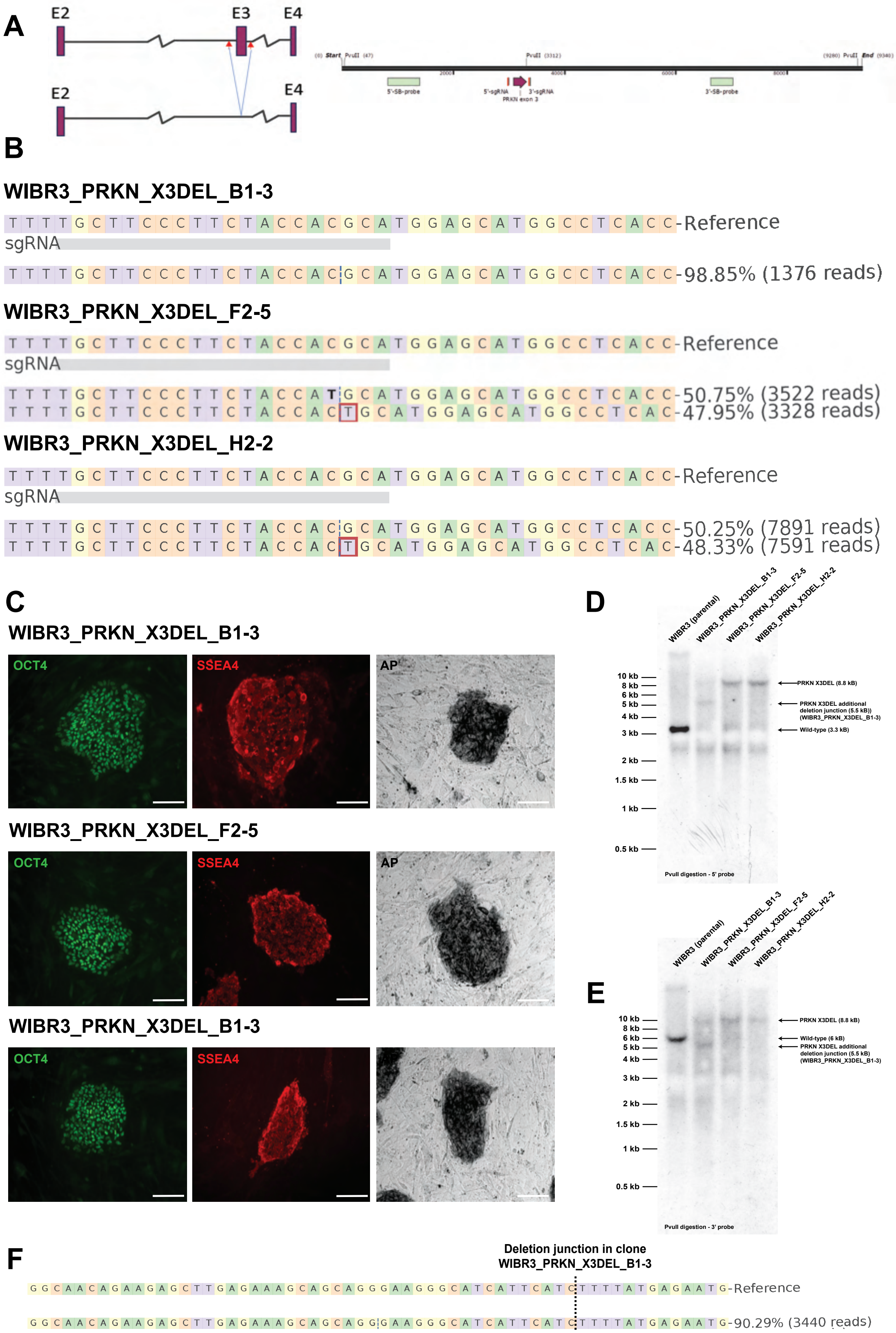

Supplemental Figure 8. Genome editing and quality control of WIBR3 hESCs carrying PD-associated deletions in *PRKN*.

(A) Schematic illustrating targeting strategy to generate PRKN X3del mutation by CRISPR/Cas9 dual guide strategy. Included are genomic location of sgRNAs, Southern blot (SB) probes and restriction enzymes used for southern blot.

(B) NGS-based genotyping to confirm correct editing for PRKN X3del in clones WIBR3_PRKN_X3DEL_B1-3, WIBR3_PRKN_X3DEL_F2-5 and WIBR3_PRKN_X3DEL_H2-2 (deletion junction). Red box indicates single base insertion. Bold bases indicate base substitution.

(C) Immunocytochemistry of hESC cultures for pluripotency markers OCT4 (green), SSEA4 (red) and alkaline phosphatase (black). Scale bar 100 µm.

(D, E) Southern blot analysis of WIBR3_PRKN_X3DEL cell lines to exclude LOH. Genomic DNA was digested with indicated enzymes and hybridized with 3' and 5' probes indicated in (A). Expected fragment size for wild type and PRKN_X3DEL allele are indicated for each digest. This analysis indicates that the clone WIBR3_PRKN_X3DEL_B1-3 carries a larger deletion on one allele.

(F) NGS analysis of the new deletion junction in the clone WIBR3_PRKN_X3DEL_B1-3.

Source data are provided as a Source Data file.

Supplementary Figure 9, Busquets et al.

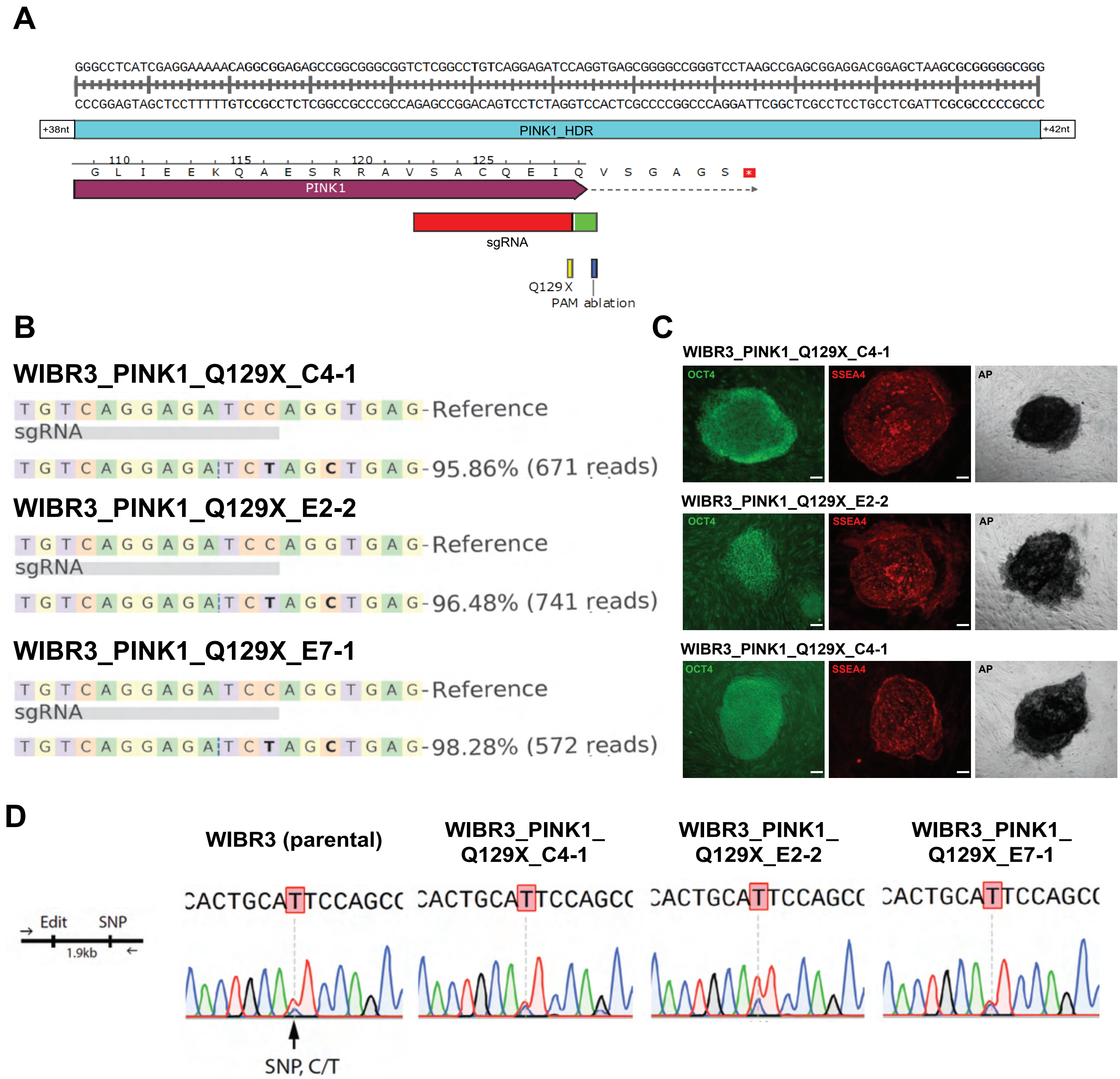

Supplementary Figure 9. Genome editing and quality control of WIBR3 hESCs carrying PD-associated mutations in *PINK1*.

(A) Schematic illustrating targeting strategy to generate PINK1 Q129X mutation by CRISPR/Cas9 facilitated HDR.

(B) NGS-based genotyping to confirm correct editing for PINK1 Q129X in clones WIBR3_PINK1_Q129X_C4-1, WIBR3_PINK1_Q129X_E2-2 and WIBR3_PINK1_Q129X_E7-1. Bold bases indicate base substitution.

(C) Immunocytochemistry of hESC cultures for pluripotency markers OCT4 (green), SSEA4 (red) and alkaline phosphatase (black). Scale bar 100 µm.

(D) Zygosity analysis using Sanger sequencing to detect heterozygous SNV to exclude LOH in any of the PINK1 Q129X clones.

Source data are provided as a Source Data file.

Supplementary Figure 10, Busquets et al.

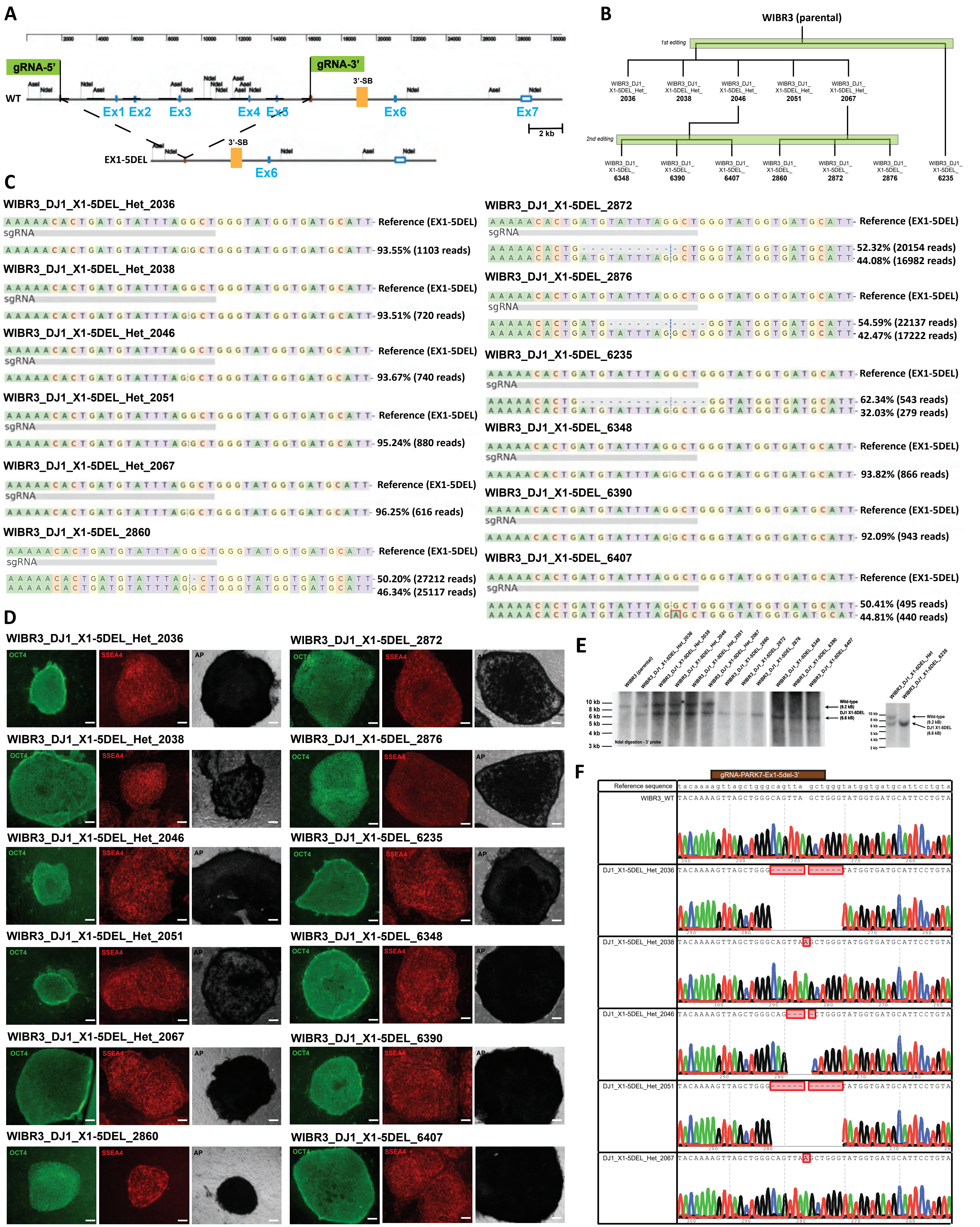

Supplementary Figure 10. Genome editing and quality control of WIBR3 hESCs carrying PD-associated deletions in DJ1/*PARK7*.

(A) Schematic illustrating targeting strategy to generate DJ1/PARK7 Ex1-5del mutation by CRISPR/Cas9 dual guide strategy. Included are genomic location of sgRNAs, Southern blot (SB) probe and restriction enzyme used for southern blot.

(B) Schematic representation of the parental lineage of heterozygous and homozygous clones carrying Ex1-5del mutation in the iSCORE-PD collection.

(C) NGS-based genotyping to confirm correct editing for DJ1/PARK7 Ex1-5del in heterozygous clones WIBR3_DJ1_X1-5DEL_Het_2036/2038/2046/2051/2067 and homozygous clones WIBR3_DJ1_X1-5DEL_2860/2872/2876/6235/6348/6390/6407 (deletion junction). “-“ indicates base deletion. Red box indicates single base insertion.

(D) Immunocytochemistry of hESC cultures for pluripotency markers OCT4 (green), SSEA4 (red), and alkaline phosphatase (black). Scale bar 100 µm.

(E) Southern blot analysis of homozygous and heterozygous WIBR3_DJ1_X1-5DEL cell lines to exclude LOH. Genomic DNA was digested with indicated enzymes and hybridized with 3' probes indicated in (A). Expected fragment size for wild type and DJ1_X1-5DEL alleles are indicated.

(F) Sanger sequencing results for secondary WT allele alteration in heterozygous cell lines after 1st editing experiment.

Source data are provided as a Source Data file.

Supplementary Figure 11, Busquets et al.

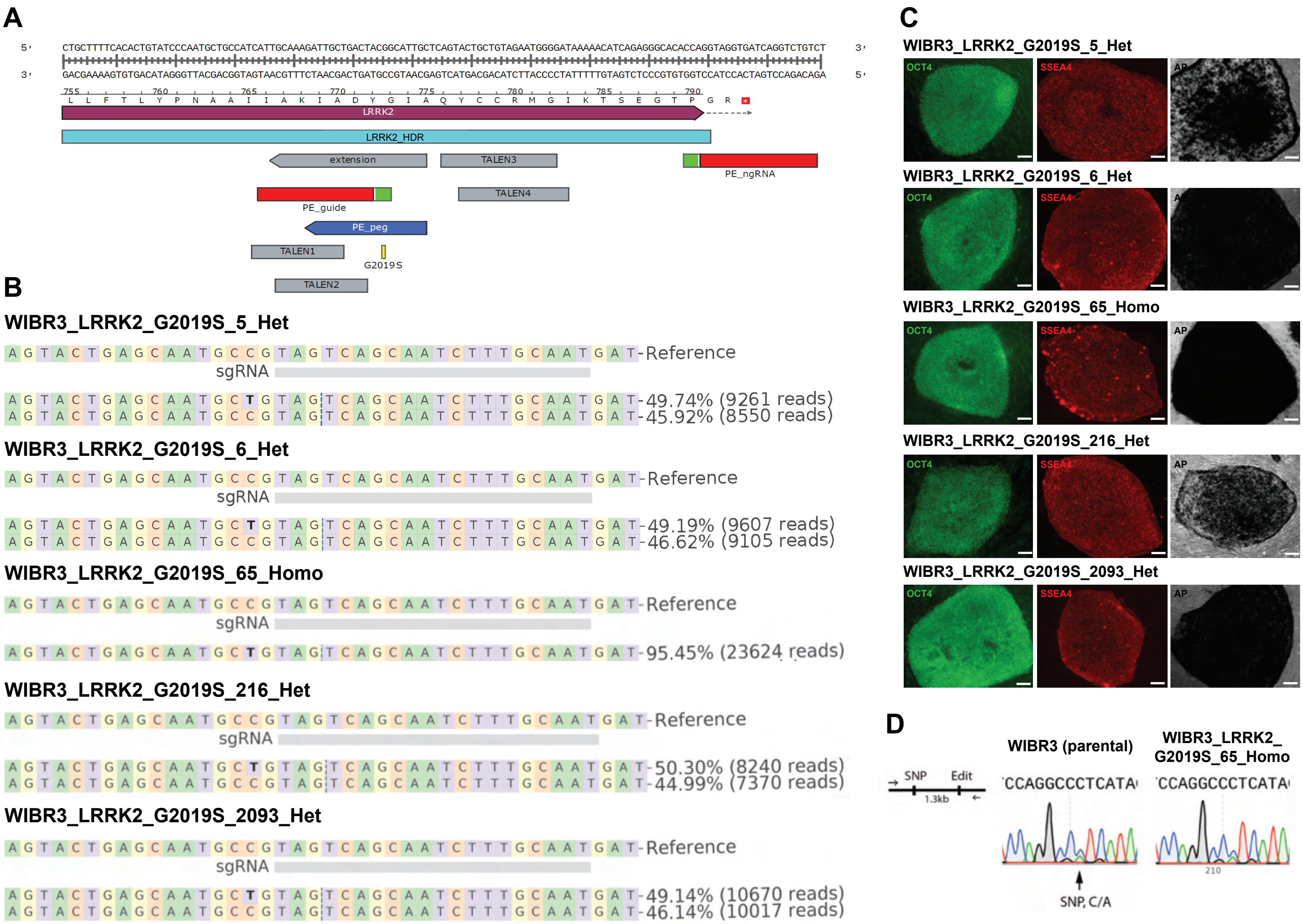

Supplementary Figure 11. Genome editing and quality control of WIBR3 hESCs carrying PD-associated mutations in *LRRK2*.

(A) Schematic illustrating targeting strategy to generate LRRK2 G2019S mutation by prime editing and TALEN or CRISPR/Cas9 facilitated HDR.

(B) NGS-based genotyping to confirm correct editing for LRRK2 G2019S in clones WIBR3_LRRK2_G2019S_5_Het, WIBR3_LRRK2_G2019S_6_Het, WIBR3_LRRK2_G2019S_65_Homo, WIBR3_LRRK2_G2019S_216_Het and WIBR3_LRRK2_G2019S_2093_Het. Bold bases indicate base substitution. The generation of the WIBR3_LRRK2_G2019S_5_Het and WIBR3_LRRK2_G2019S_6_Het cell lines was already reported[73].

(C) Immunocytochemistry of hESC cultures for pluripotency markers OCT4 (green), SSEA4 (red) and alkaline phosphatase (black). Scale bar 100 µm.

(D) Zygosity analysis using Sanger sequencing to detect heterozygous SNV to exclude LOH in cell line WIBR3_LRRK2_G2019S_65_Homo.

Source data are provided as a Source Data file.

Supplementary Figure 12, Busquets et al.

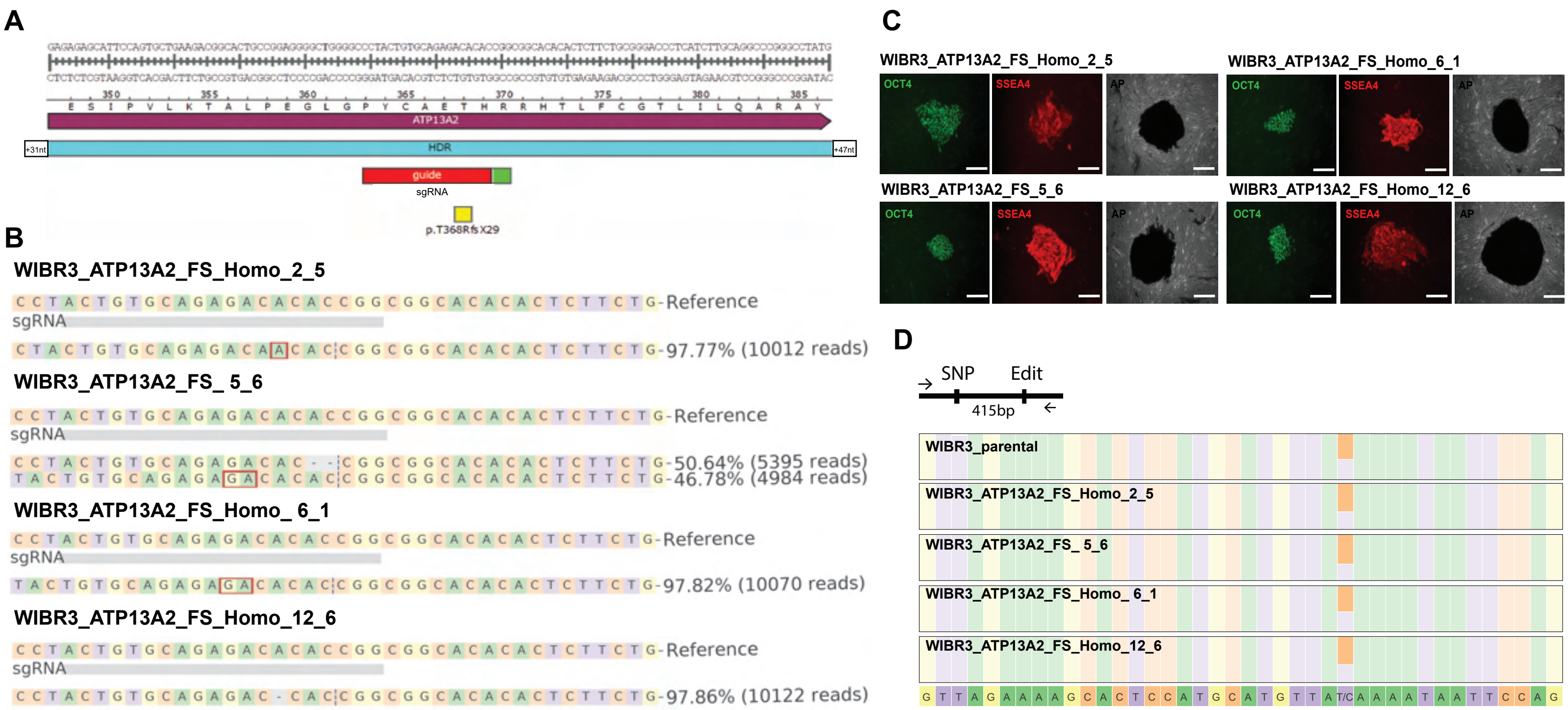

Supplementary Figure 12. Genome editing and quality control of WIBR3 hESCs carrying PD-associated alterations in *ATP13A2*.

(A) Schematic illustrating targeting strategy to generate CRISPR/Cas9 mediated frameshift mutation in ATP13A2.

(B) NGS-based genotyping to confirm a frameshift mutation in ATP13A2 in clones WIBR3_ATP13A2_FS_Homo_2_5, WIBR3_ATP13A2_FS_5_6, WIBR3_ATP13A2_FS_Homo_6_1, and WIBR3_ATP13A2_FS_Homo_12_6. Red box indicates base insertion. "-" indicates base deletion.

(C) Immunocytochemistry of hESC cultures for pluripotency markers OCT4 (green), SSEA4 (red) and alkaline phosphatase (black). Scale bar 100 µm.

(D) Zygosity analysis using NGS sequencing to detect heterozygous SNV to exclude LOH in all ATP13A2 clones.

Source data are provided as a Source Data file.

Supplementary Figure 13, Busquets et al.

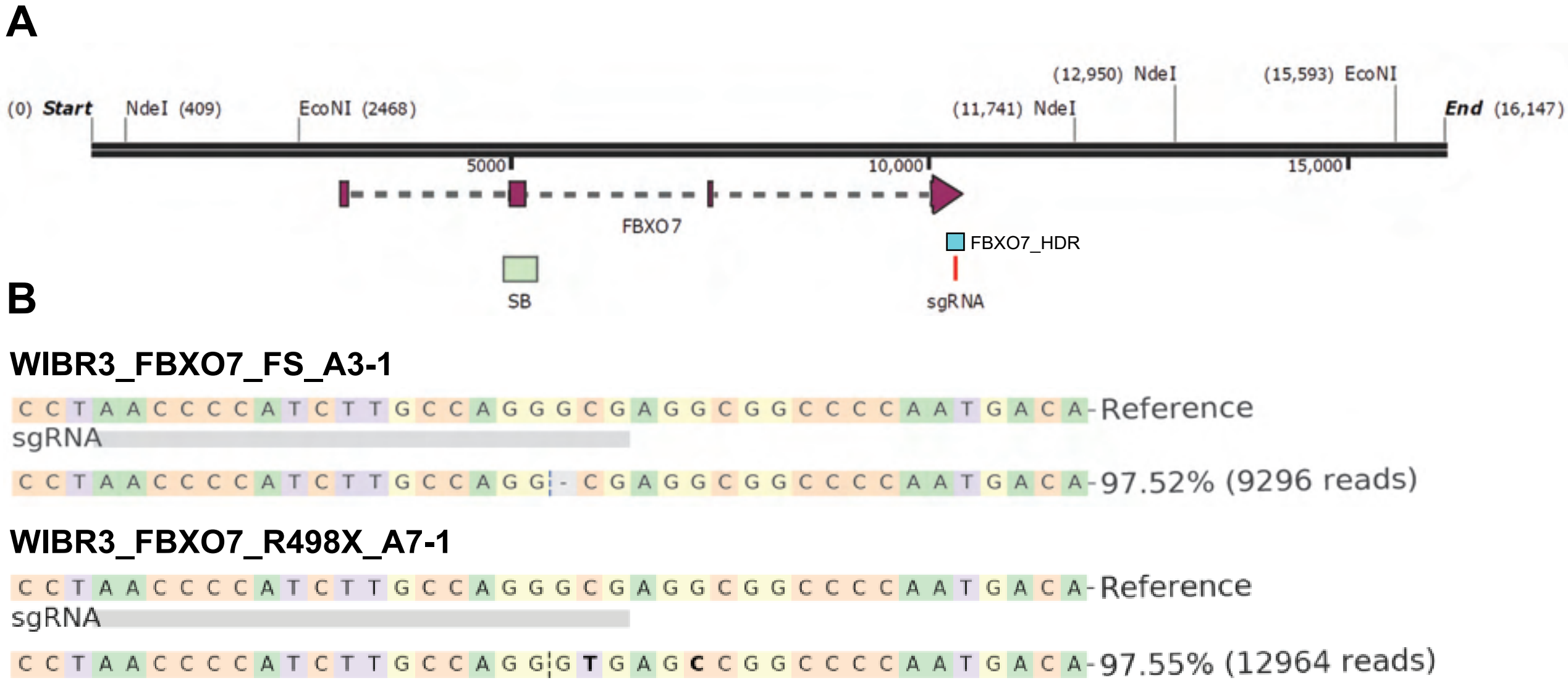

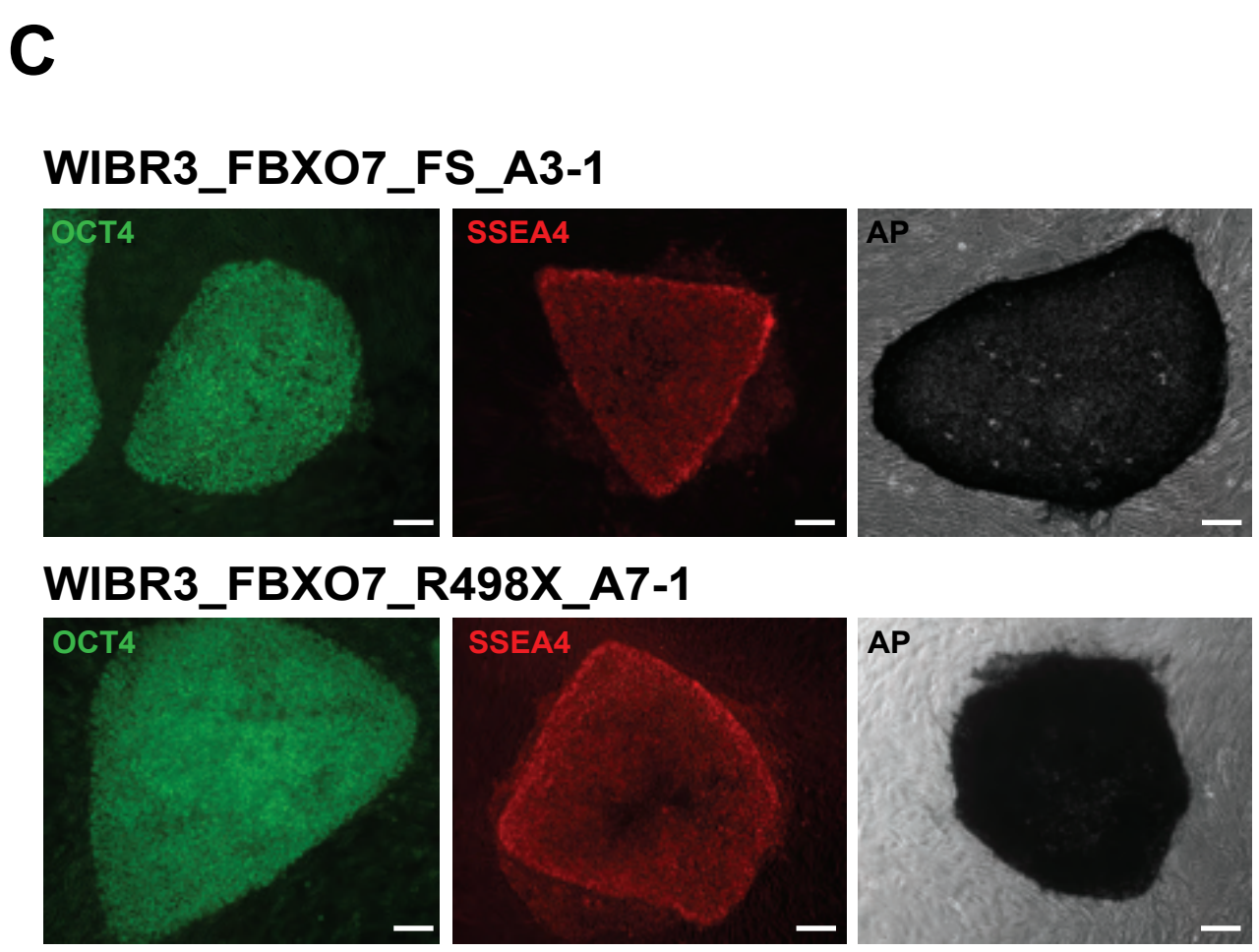

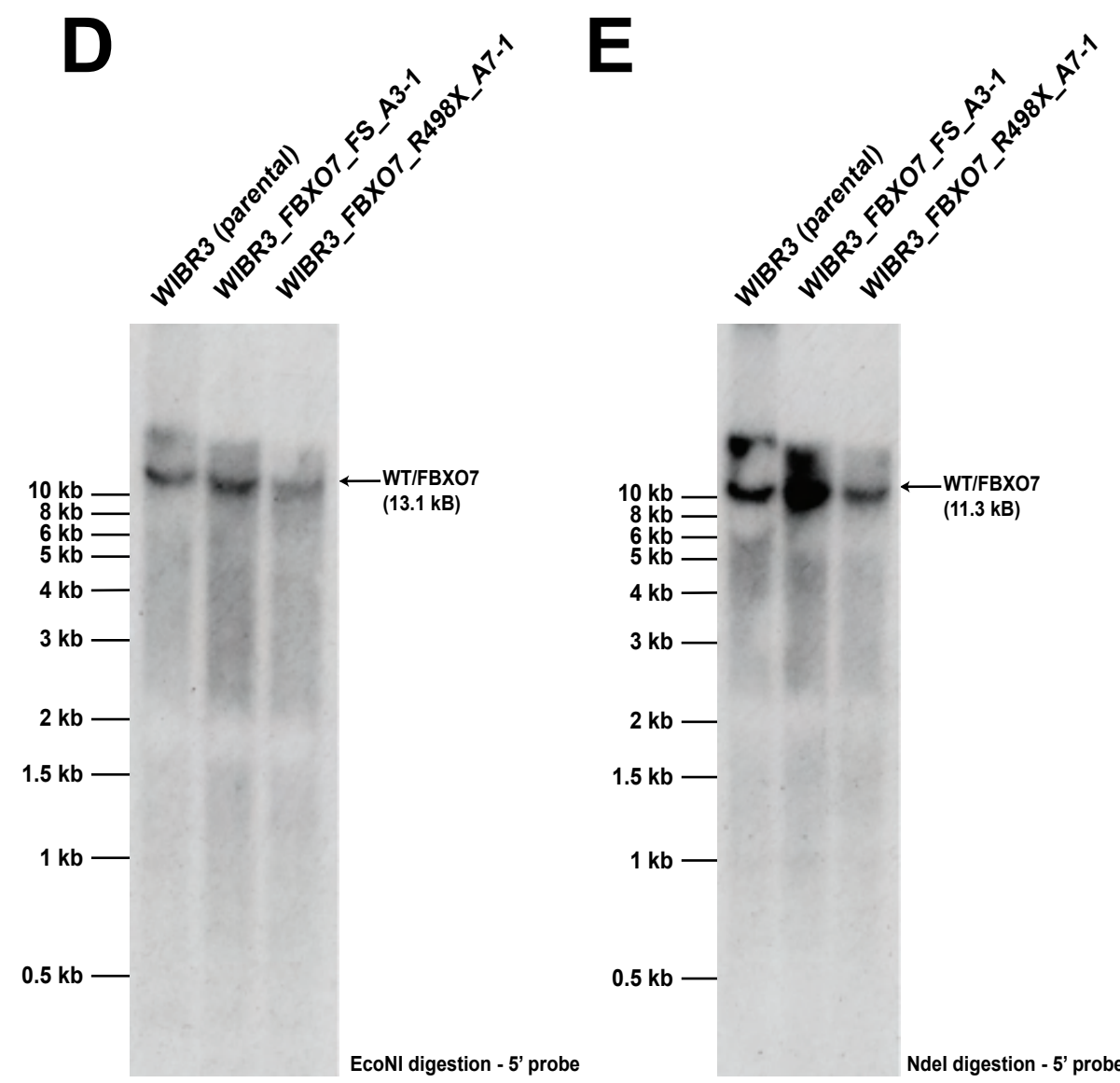

F

Cell line	Genotypes	Number of reads
**chr22:32498038, upstream closest SNP**		
WIBR3_parental	T/G	11,17
WIBR3_FBXO7_FS_A3-1	T/G	21,16
WIBR3_FBXO7_R498X_A7-1	T/G	12,11
**chr22:32500817, downstream closest SNP**		
WIBR3_parental	G/A	15,9
WIBR3_FBXO7_FS_A3-1	G/A	21,20
WIBR3_FBXO7_R498X_A7-1	G/A	9,18

Supplementary Figure 13. Genome editing and quality control of WIBR3 hESCs carrying PD-associated alterations in *FBXO7*.

(A) Schematic illustrating targeting strategy to generate CRISPR/Cas9-mediated R498X/frameshift mutation in FBXO7. Included are genomic location of sgRNA, Southern blot (SB) probe and restriction enzymes used for southern blot.

(B) NGS-based genotyping to confirm a frameshift in mutation in FBXO7 in clones WIBR3_FBXO7_FS_A3-1 and WIBR3_FBXO7_R498X_A7-1. “-“ indicates base deletion. Bold bases indicate base substitution.

(C) Immunocytochemistry of hESC cultures for pluripotency markers OCT4 (green), SSEA4 (red) and alkaline phosphatase (black). Scale bar 100 µm.

(D, E) Southern blot analysis to exclude LOH. Genomic DNA was digested with indicated enzymes and hybridized with a 5' probe indicated in (A). Expected fragment size for wild type and FBXO7 frameshift alleles are indicated for each digest.

(F) WGS data was used to exclude LOH of FBXO7 clones by assessing closest upstream and downstream SNVs. Source data are provided as a Source Data file.

Supplementary Figure 14, Busquets et al.

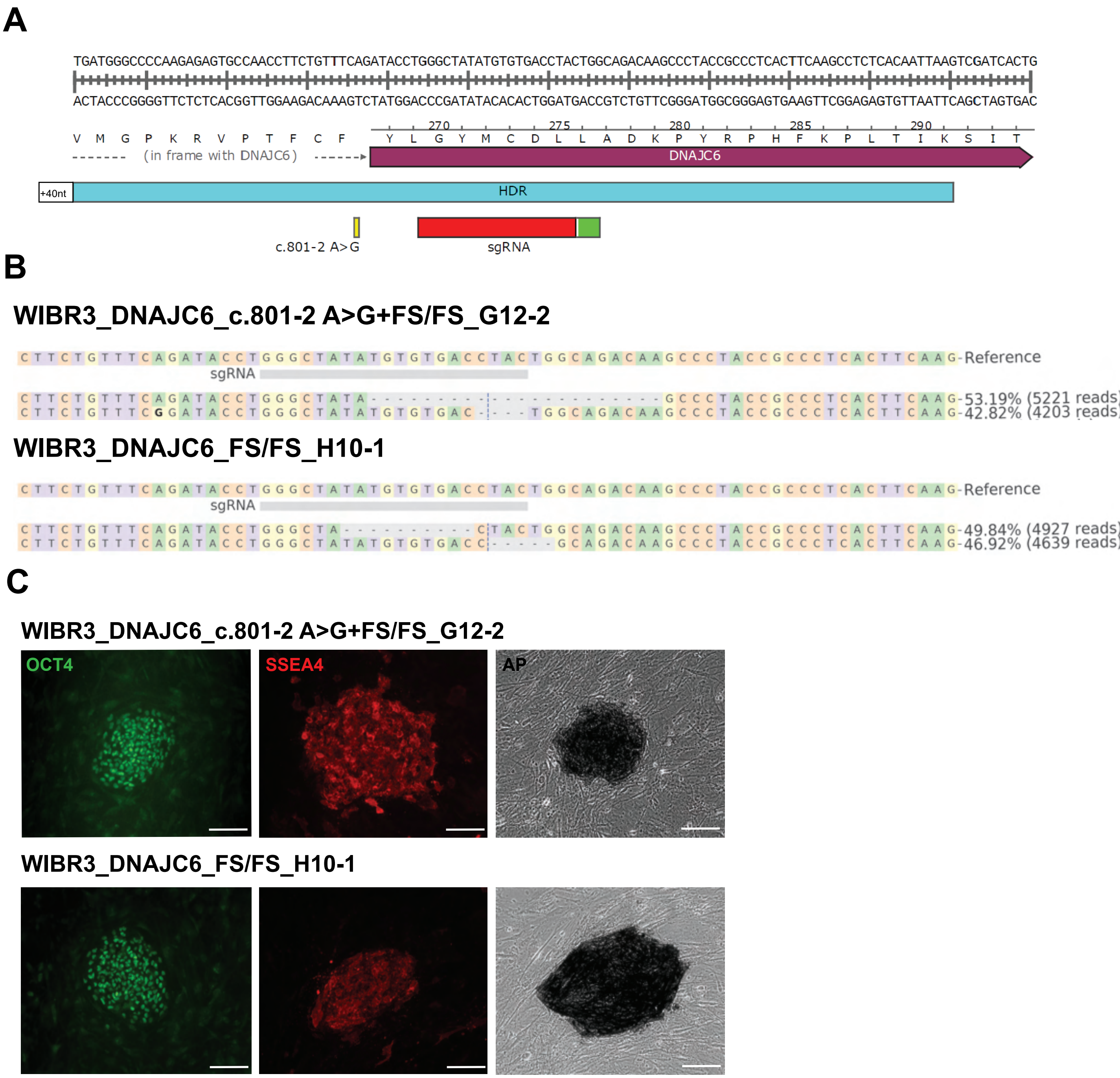

Supplementary Figure 14. Genome editing and quality control of WIBR3 hESCs carrying PD-associated alterations *DNAJC6*.

A) Schematic illustrating targeting strategy to generate DNAJC6 c.801-2 A>G/FS mutation by CRISPR/Cas9 facilitated HDR.

(B) NGS-based genotyping to confirm correct editing for DNAJC6 c.801-2 A>G and frameshift mutation in clones WIBR3_DNAJC6_c.801-2 A>G+FS/FS_G12-2 and WIBR3_DNAJC6_FS/FS_H10-1. “-“ indicates base deletion.

(C) Immunocytochemistry of hESC cultures for pluripotency markers OCT4 (green), SSEA4 (red) and alkaline phosphatase (black). Scale bar 100 µm.

Source data are provided as a Source Data file.

Supplementary Figure 15, Busquets et al.

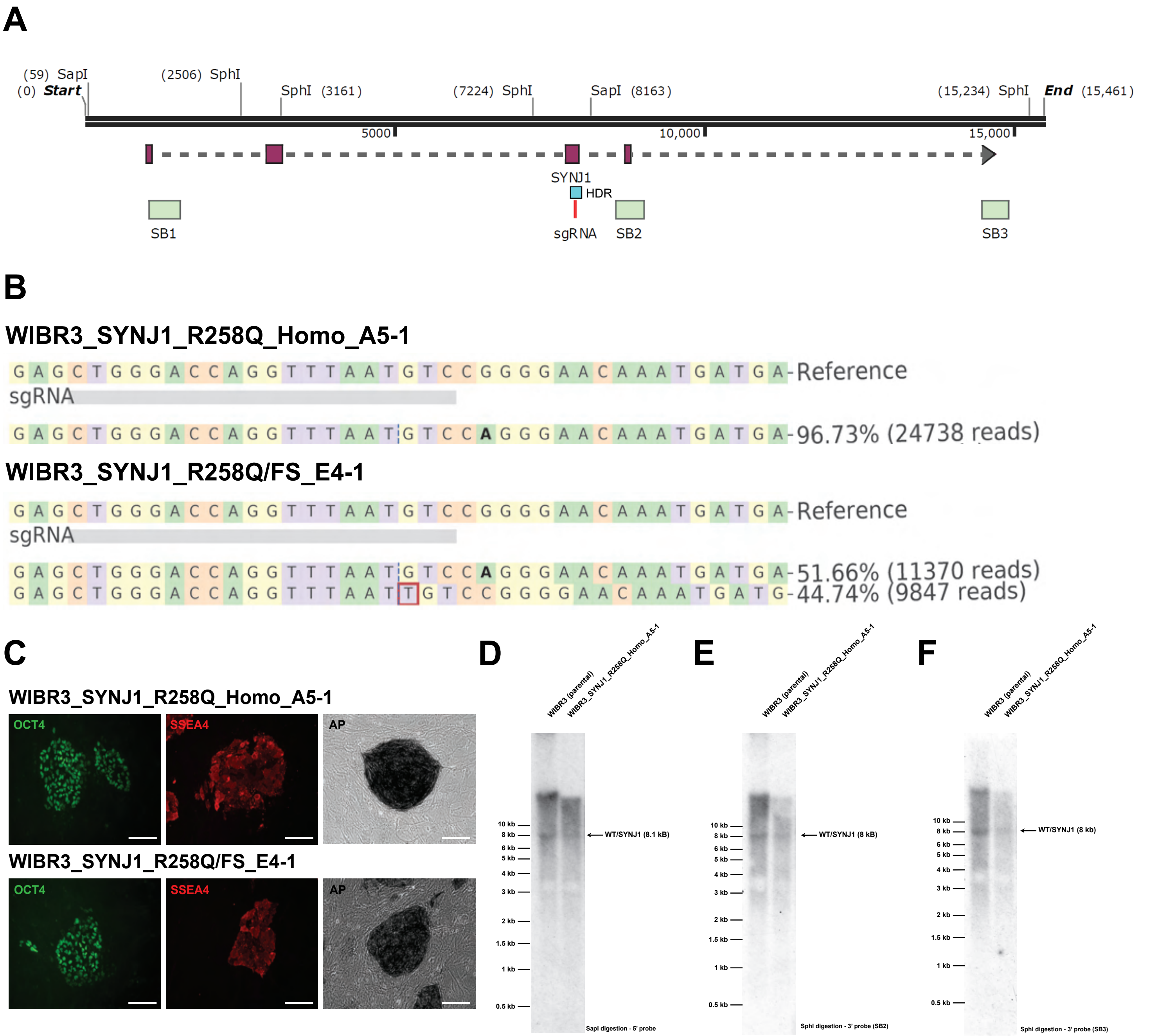

Supplementary Figure 15. Genome editing and quality control of WIBR3 hESCs carrying PD-associated alterations *SYNJ1*.

(A) Schematic illustrating targeting strategy to generate SYNJ1 R258Q/FS mutation by CRISPR/Cas9 facilitated HDR.

(B) NGS-based genotyping to confirm correct editing for SYNJ1 R258Q and frameshift mutation in clones WIBR3_SYNJ1_R258Q_Homo_A5-1 and WIBR3_SYNJ1_R258Q/FS_E4-1. Bold bases indicate base substitution. Red box indicates base insertion.

(C) Immunocytochemistry of hESC cultures for pluripotency markers OCT4 (green), SSEA4 (red) and alkaline phosphatase (black). Scale bar 100 µm.

(D-F) Southern blot analysis of WIBR3_SYNJ1 cell lines to excluded LOH. Genomic DNA was digested with indicated enzymes and hybridized with probes indicated in (A). Expected fragment size for wild-type and SYNJ1 alleles are indicated for each digest.

Source data are provided as a Source Data file.

Supplementary Figure 16, Busquets et al.

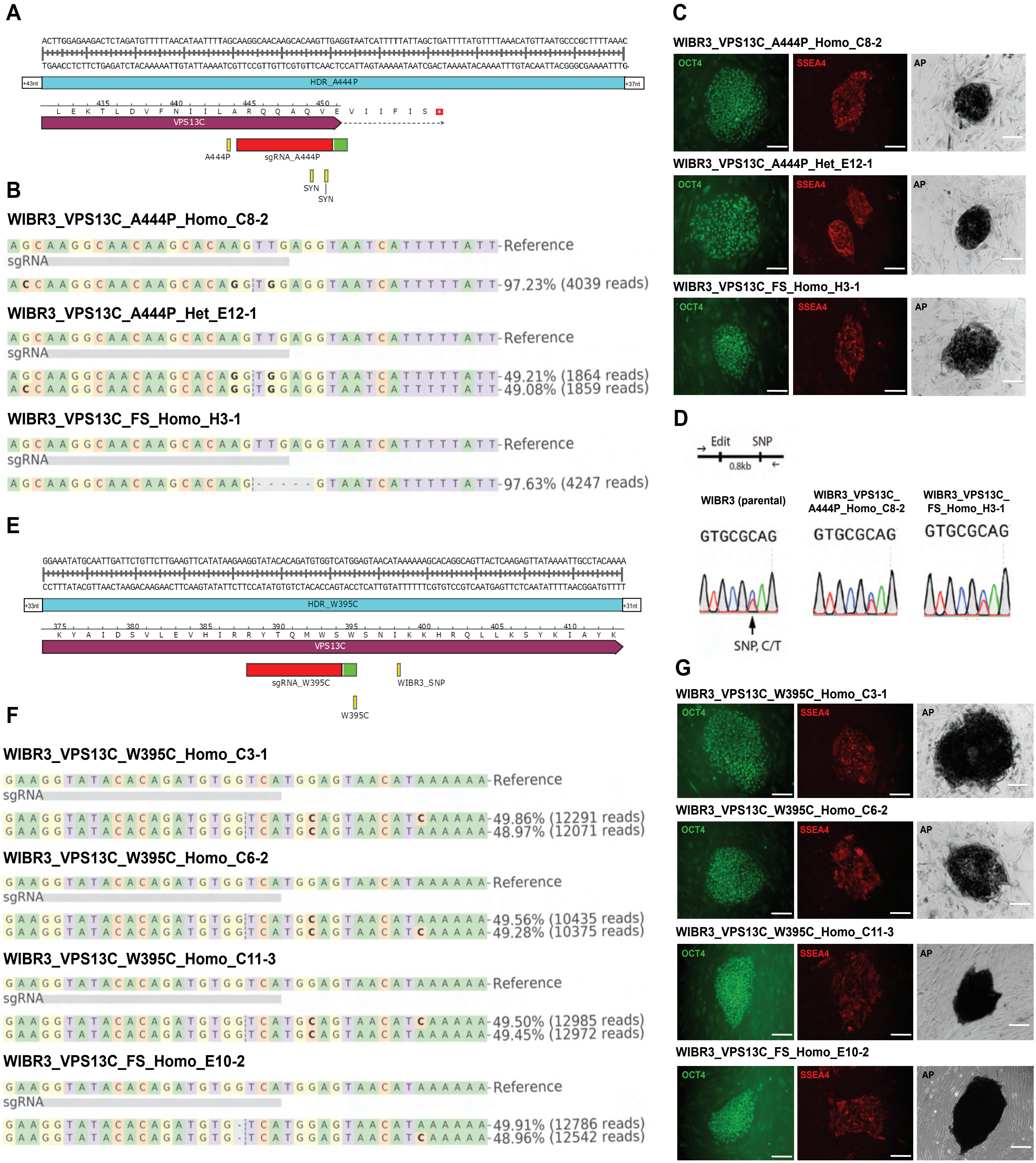

Supplemental Figure 16. Genome editing and quality control of WIBR3 hESCs carrying PD-associated alterations *VPS13C*.

(A) Schematic illustrating targeting strategy to generate VPS13C A444P mutation by CRISPR/Cas9 facilitated HDR. Included are genomic location of the A444P mutation, sgRNA, ssODN template including synonymous ssODN mutations (SYN) in sgRNA-target site to prevent re-cutting of edited alleles.

(B) NGS-based genotyping to confirm correct editing for A444P and frameshift mutation in VPS13C in the clones WIBR3_VPS13C_A444P_Homo_C8-2, WIBR3_VPS13C_A444P_Het_E12-1 and WIBR3_VPS13C_FS_Homo_H3-1. Bold bases indicate base substitution. "-" indicates base deletion.

(C) Immunocytochemistry of hESC cultures for pluripotency markers OCT4 (green), SSEA4 (red) and alkaline phosphatase (black). Scale bar 100 µm.

(D) Zygosity analysis using Sanger sequencing to detect heterozygous SNV to exclude LOH in clones WIBR3_VPS13C_A444P_Homo_C8-2 and WIBR3_VPS13C_A444P_Homo_H3-1.

(E) Schematic illustrating targeting strategy to generate VPS13C W395C and frameshift mutation by CRISPR/Cas9 facilitated HDR.

(F) NGS-based genotyping to confirm correct editing for VPS13C W395C and frameshift mutation in the clones WIBR3_VPS13C_W395C_Homo_C3-1, WIBR3_VPS13C_W395C_Homo_C6-2, WIBR3_VPS13C_W395C_Homo_C11-3 and WIBR3_VPS13C_FS_Homo_E10-2. Bold bases indicate base substitution. "-" indicates base deletion.

(G) Immunocytochemistry of hESC cultures for pluripotency markers OCT4 (green), SSEA4 (red) and alkaline phosphatase (black). Scale bar 100 µm.

Source data are provided as a Source Data file.

Supplementary Figure 17, Busquets et al.

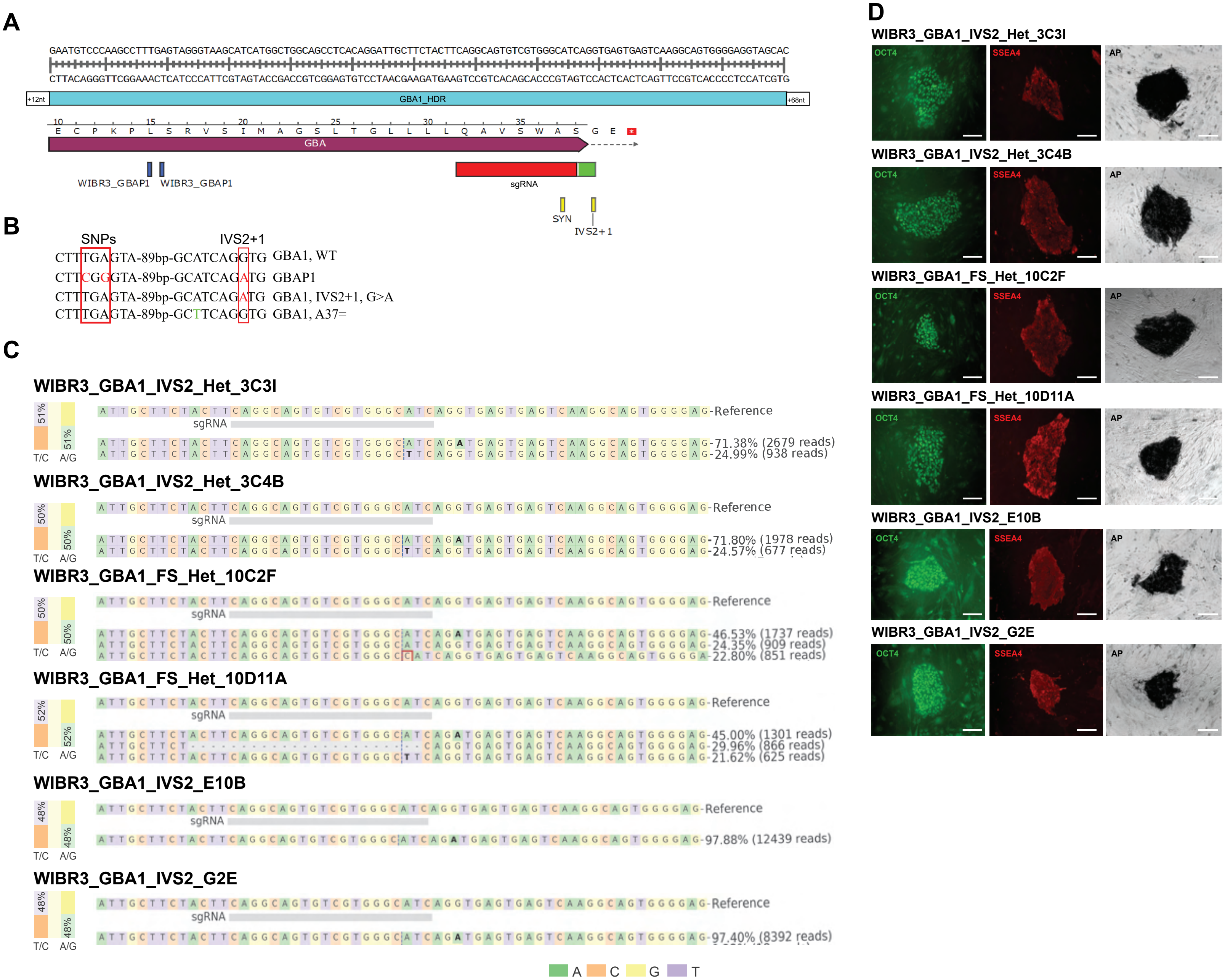

Supplementary Figure 17. Genome editing and quality control of WIBR3 hESCs carrying PD-associated alterations *GBA1*.

(A) Schematic illustrating targeting strategy to generate GBA1 IVS2+1 mutation by CRISPR/Cas9 facilitated HDR. To generate the heterozygous mutations a competing HDR template (ssODNs) containing synonymous mutations (SYN) in the sgRNA-target site was used.

(B) Sequences alignment of the wild type GBA1 gene (GBA1, WT), its pseudogene (GBAP1), the engineered GBA1 IVS2+1 mutation (GBA1, IVS2+1 G>A) and synonymous mutation (GBA1, A37=) in the targeted region. The mutated nucleotides and SNVs used to calculate the allelic balance between GBA1 and GBAP1 are highlighted in red boxes.

(C) NGS-based genotyping to confirm correct editing of the GBA IVS2+1 mutation and evaluate proper zygosity balance with GBAP1 for Clones WIBR3_GBA_IVS2_Het_3C3I, WIBR3_GBA_IVS2_Het_3C4B, WIBR3_GBA_FS_Het_10C2F, WIBR3_GBA_IVS2_E10B and WIBR3_GBA_IVS2_G2E. Bold bases indicate base substitution. Red box indicates base insertion.

(D) Immunocytochemistry of hESC cultures for pluripotency markers OCT4 (green), SSEA4 (red) and alkaline phosphatase (black). Scale bar 100 µm.

Source data are provided as a Source Data file.

Supplementary Figure 18, Busquets et al.

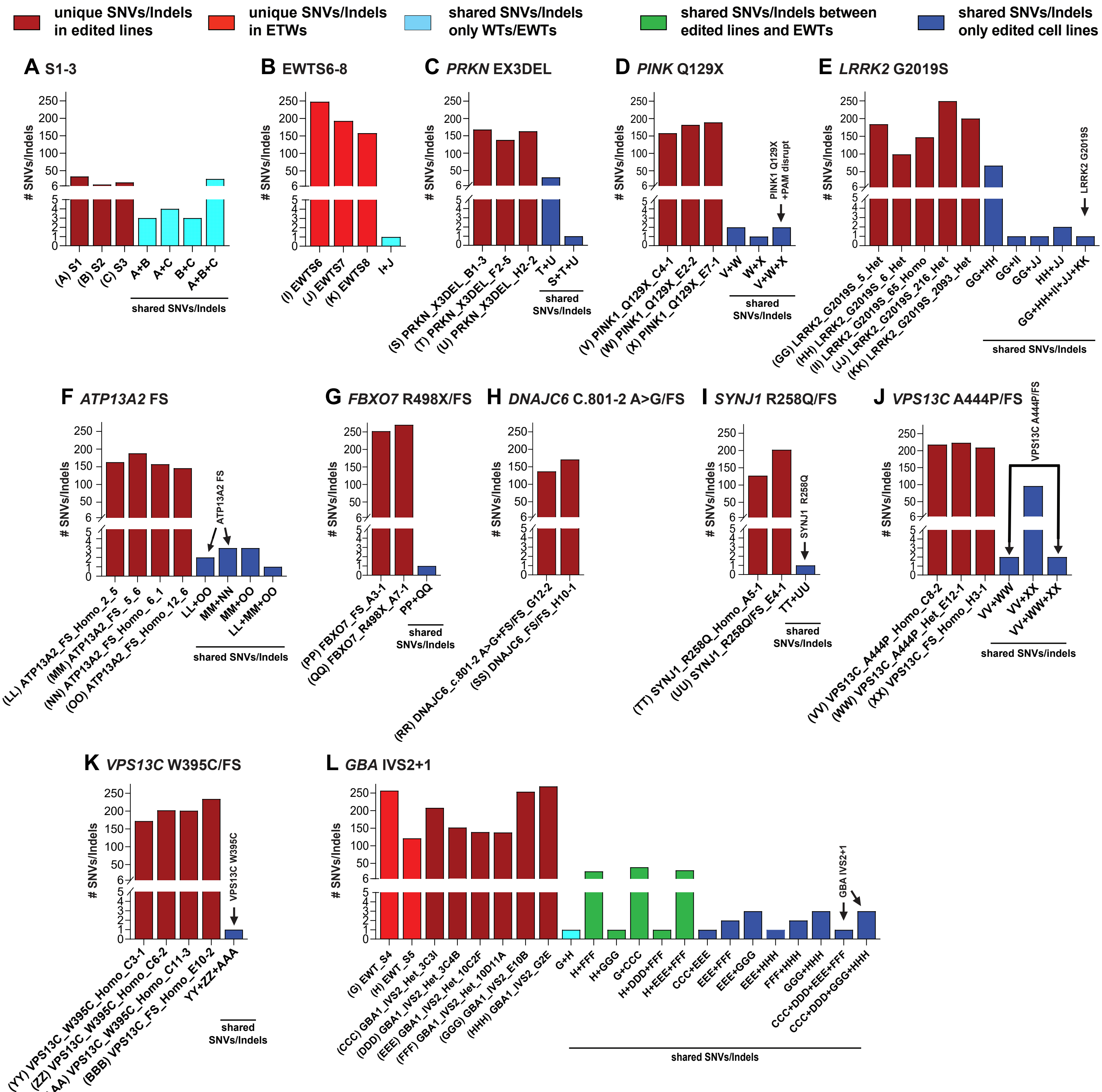

Supplementary Figure 18. Graph showing number of unique and shared SNVs/indels in each editing experimental group: (A) WIBR3-S1/S2/S3, (B) WIBR3_EWTS6/7/8, (C) PRKN EX3DEL, (D) PINK1 Q129X, (E) LRRK2 G2019S, (F) ATP13A2 FS, (G) FBXO7 R498X/FS, (H) DNAJC6 C.801-2 A>G/FS, (I) SYNJ1 R258Q/FS, (J) VPS13C A444P/FS, (K) VPS13C W395C/FS and (L) GBA IVS2+1. Different color bars indicate unique or shared SNVs/Indels between different cell lines. Dark red indicates unique SNVs/Indels in edited lines, light red indicates unique SNVs/Indels in EWT lines, light blue indicates shared SNVs/Indels only found on WTs/EWTs, green indicates shared SNVs/Indels between edited lines and EWTs and dark blue indicates shared SNVs/Indels only found in edited cell lines.
Source data are provided as a Source Data file.

Supplementary Note 1:

<u>iSCORE-PD collection of isogenic hPSC lines carrying PD-associated mutations.</u>

For our initial iSCORE-PD collection, we prioritized engineering cell lines carrying mutations in high confidence PD genes[1]. The specific modifications for each gene were selected based on information in the MDSgene database[2] (https://www.mdsgene.org) and the currently available literature as outlined for each gene below. Overall, 65 clonal cell lines carrying PD-associated mutations in 11 genes linked to PD along with isogenic control lines passed all the above-described quality control steps and have become part of the iSCORE-PD collection (Table 1, Supplemental Table 3). The current iteration of the iSCORE-PD includes the following cell lines:

Control cell lines

Together with the parental WIBR3 cell line, we provide a set of subclones with this collection (WIBR3-S1, S2, S3) (Figure 1, Supplemental figure 1). In addition, we included WIBR3 cell lines that were isolated as part of the standard genome editing pipeline but did not exhibit any genetic modifications at the targeted locus. We consider these as "edited wild-type" cells (EWT), which are the best experimental control to account for any non-specific changes caused by the gene editing process (EWT1-3: prime editing controls - Pipeline B, EWT4-5: CRISPR/Cas9 controls - Pipeline B and EWT6-8: prime editing controls - Pipeline A) (Table1, Supplemental Table 3, Supplemental Figure 6).

SNCA (PARK1)

The *SNCA* gene encodes the alpha-synuclein (α-Syn) protein. The discovery that mutations and copy number increases of the *SNCA* gene linked to familial forms of PD, along with the identification of non-coding variants in the *SNCA* locus as a risk factor for sporadic PD, indicate a central pathogenic role for this protein[3]. Moreover, fibrillar α-Syn is the major component of Lewy bodies, and changes in the dosage, aggregation, and clearance properties of α-Syn are thought to be central pathogenic drivers of PD[1,3-5]. We established prime editing reagents to efficiently introduce the A30P or A53T mutation in the *SNCA* gene, both of which are linked to autosomal dominant forms of PD[6-8]. Using this approach (Supplemental Figure 7A,D), we generated cell lines that include three cell lines carrying the A53T mutation (heterozygous) (Supplemental Figure 7B,C) and four cell lines carrying the A30P mutation (3 heterozygous and 1 homozygous) (Supplemental Figure 7E,F). All cell lines passed the described quality control steps. In addition, we performed SNV-PCR followed by Sanger sequencing-based zygosity analysis to exclude LOH in the homozygous *SNCA* A30P line (Supplemental Figure 7G). Based on the described WGS analysis, we recommend including EWT_S1-3 as controls in disease modeling experiments for the cell lines carrying the A30P mutation.

PRKN (PARK2)

Mutations in the *PRKN* gene, which encodes the E3 ubiquitin-protein ligase parkin (Parkin), are the most frequent cause for autosomal recessive PD[9]. The first genetic alterations in *PRKN* linked to PD were distinct large-scale deletions[10]. Since then, a wide range of deletions and point mutations have been identified[11]. Molecular alterations in Parkin impact a wide range of molecular and cellular functions

including responses to oxidative stress, mitochondrial membrane potential, mitochondrial motility, contact sites with the endoplasmic reticulum, and the regulation of inflammatory responses[12,13]. Homozygous and compound heterozygous deletions of Exon3 (Ex3del) are repeatedly found in families with PD, and Ex3del is now a confirmed pathogenic variant of *PRKN*[11,14]. We used a dual CRISPR/Cas9 approach (Figure 3C[v]) targeting each side of Exon3 to generate 3 cell lines that are homozygous for the Ex3del (Supplemental Figure 8A-C). Subsequent southern blot analysis to exclude LOH revealed that one cell line (WIBR3_PRKN_X3DEL_B1-3) carries a larger Ex3del (~3.8 kb, Supplemental Figure 8D,E), which we fully characterized by NGS sequencing (Supplemental Figure 8F). We included this cell line in the iSCORE-PD collection since the flanking exons 2 and 4 are not affected by the deletion and thus, no significant consequences are expected of the extended deletion. No heterozygous clones have been included in this collection since these genotypes are frequent in control populations and are not associated with a higher risk of PD[15].

***PINK1* (PARK6)**

Mutations in *PINK1* were first associated with PD in 2004 following earlier studies linking the PARK6 genomic region with increased risk for PD[16]. Subsequently, a large number of point mutations, frameshift mutations and deletions have been identified, predominantly affecting the activity of the kinase domain[11]. This indicates that *PINK1* loss-of-function is the cause for early-onset, autosomal recessive PD. The serine/threonine-protein kinase PINK1 plays a crucial role in mitochondrial quality control by regulating mitochondrial homeostasis and clearance[17]. Based on the segregation of the Q129X and a related Q129fsX157 mutation in *PINK1* observed in two large families with PD[18], we generated 3 cell lines that are homozygous for the Q129X mutation in *PINK1* (Supplemental Figure 9A-C). To exclude LOH, we performed SNV-PCR followed by Sanger sequencing based zygosity analysis (Supplemental Figure 9D). Heterozygous *PINK1* mutant cell lines were not included in the iSCORE-PD collection, as the heterozygous genotype is not considered a risk factor for PD.

***DJ1* (PARK7)**

Since its initial association with PD[19], multiple point mutations and genomic rearrangements in *PARK7* (*DJ1*), encoding the Parkinson disease protein 7, have been identified as a rare cause for autosomal recessive PD[11]. While the exact function of this enzyme remains largely unknown, Parkinson disease protein 7 is implicated in regulating transcription, cell growth and oxidative stress response pathways linked to cell survival and apoptosis[20-22]. Based on the identification of homozygous deletions of either exon 5[23] or exon 1 to 5[24] in families with PD, we used two step dual gRNA CRISPR/Cas9 approach to closely recapitulate the Exon 1 to 5 deletion (Supplemental Figure 10A-D). As outlined in detail above, the WGS analysis revealed a high number of shared SNVs/indels between the initially established clones (WIBR3_DJ1_X1-5DEL_2860/2872/2876).

To account for the potential impact of these shared variants on phenotypical analyses, we screened for an additional homozygous clone that was generated in a single targeting step (WIBR3_DJ1_X1-5DEL_6235). Additionally, we included three other homozygous DJ1/PARK7 clones (WIBR3_DJ1_EX1-5DEL_6348/6390/6407), which were generated by retargeting a second heterozygous cell line (WIBR3_DJ1_X1-5DEL_2046) that did not share SNVs/indels with the previously described

homozygous clones (WIBR3_DJ1_X1-5DEL_2860/2872/2876). As there is currently no evidence that heterozygous genotypes confer an increased risk of developing PD[25], we included several heterozygous DJ1/PARK7 lines as experimental controls (WIBR3_DJ1_X1-5DEL_Het_2036/2038/2046/2051/2067) that should account for the genetic variability of the homozygous targeted DJ1/PARK7 clones (Supplemental Figure 10). Southern blot analysis was performed to exclude LOH in the homozygous exon 1-5 deleted cell lines (Supplemental Figure 10E). Sanger sequencing of the wild-type allele in the heterozygous clones (WIBR3_DJ1_X1-5DEL_Het_2036/2038/2046/2051/2067) revealed additional SNVs/Indels at the target sites of the gRNAs used to generate these cell lines (Supplemental Figure 10F).

***LRRK2* (PARK8)**

Mutations in the *LRRK2* gene, encoding the leucine rich repeat serine/threonine-protein kinase 2, were first linked to PD in 2004[26]. Over the years, more than 100 different variants of the gene have been described[27], establishing *LRRK2* coding variants as the most frequently mutated gene linked to dominant and sporadic forms of PD. Among these mutations, G2019S is the most common substitution identified across populations[28], found in approximately 4% of dominantly inherited familial PD cases, in both heterozygous and homozygous forms, and in around 1% of sporadic PD cases[29]. This variant increases the kinase activity of leucine rich repeat serine/threonine-protein kinase 2, affecting a wide range of cellular and molecular processes including vesicular trafficking and cytoskeleton dynamics, autophagy and lysosomal degradation, neurotransmission, mitochondrial function, and immune and microglial responses[30,31]. We recently established CRISPR/Cas9, TALEN and prime editing reagents to efficiently introduce the G2019S mutation in the *LRRK2* gene[6]. Using this approach (Supplemental Figure 11A), we generated additional cell lines so that the iSCORE-PD collection now includes 5 clones carrying G2019S (4 heterozygous and 1 homozygous) (Supplemental Figure 11B-C). We performed SNV-PCR followed by Sanger sequencing based zygosity analysis to exclude LOH in the homozygous *LRRK2* G2019S line (Supplemental Figure 11D).

***ATP13A2* (PARK9)**

Mutations in the *ATP13A2* gene, encoding the polyamine-transporting ATPase 13A2 (ATP13A2) protein, were identified as a cause for PD in the PARK9 locus in 2006, with this genomic region previously associated with Kufor Rakeb disease[32]. Various mutations in the gene result in a truncated ATP13A2 protein causing its mis-localization from the lysosome to the endoplasmic reticulum, where it accumulates before being eventually targeted for proteasomal degradation in the cytoplasm[32,33]. Loss of ATP13A2 is linked to mitochondrial and lysosomal dysfunction, as well as to the accumulation of α-Syn due to the dysregulation of proteasomal and autophagy-mediated protein degradation[34-38]. Based on the identification of a homozygous frameshift mutation (NP_071372.1, T368RfsX29) in a family with recessive PD[38], we used CRISPR/Cas9-based genome editing to engineer the frameshift mutation around the amino acid T368 in the *ATP13A2* gene (Supplemental Figure 12A). Given the recessive inheritance pattern of the gene, our collection contains 4 clones with biallelic frameshift mutations, predicted to result in a truncated loss of function protein similar to that observed in PD patients (Supplemental Figure 12B,C). We performed SNV-PCR followed by NGS sequencing-based zygosity analysis to exclude LOH in the homozygous edited cell lines (Supplemental Figure 12D).

***FBXO7* (PARK15)**

Mutations in the *FBXO7* gene, encoding the F-box only protein 7 (FBOX7), were first linked to early onset, autosomal recessive PD in 2008[39]. One of the best-characterized variants, identified in multiple affected pedigrees, is the homozygous R498X nonsense mutation, which introduces a premature stop codon and produces a truncated protein lacking part of the C-terminal proline-rich region (PRR)[40]. This truncation leads to abnormal localization and reduced protein stability, suggesting that R498X behaves as a loss-of-function (LOF) allele. Early studies proposed that FBXO7 regulates proteasomal activity and facilitates the recruitment of PINK1 and Parkin to damaged mitochondria, thereby promoting mitophagy[41-43]. However, more recent studies have challenged this view, showing that FBXO7 is dispensable for PINK1/Parkin-mediated mitophagy and does not broadly impair proteasome function under basal conditions, suggesting a more context-dependent role[44]. We used CRISPR/Cas9-based genome editing to introduce the patient-associated R498X mutation and a frameshift mutation at the same position. While R498X produces a truncated protein, the frameshift mutation replaces the native C-terminus with a non-native sequence, both of which disrupt the C-terminal proline-rich region (PRR), predicted to be important for FBXO7 stability (Supplemental Figure 13A-C). We performed Southern blot, SNV-PCR followed by NGS sequencing and analysis of WGS data to exclude LOH (Supplemental Figure 13D-F).

***DNAJC6* (PARK19)**

The DNAJC6 gene encodes for the protein putative tyrosine-protein phosphatase AUXILIN, a member of the DNAJ/HSP40 family of proteins, which regulate molecular chaperone activity. DNAJC6 was initially linked to very early onset, autosomal recessive PD in 2012[45] through the identification of a mutation that affects splicing and the expression of Auxilin. Consistent with its role as a co-chaperone that recruits HSC70 to clathrin-coated vesicles, hESC models show that *DNAJC6* alterations result in loss of Auxilin protein. This leads to the accumulation of clathrin, reduced vesicular transport, and the degeneration of midbrain dopaminergic neurons[46]. Furthermore, these alterations are associated with α-Syn aggregation, mitochondrial and lysosomal dysfunction, and lipid defects[46-48]. We used a CRISPR/Cas9-based approach to recapitulate the effect of the c.801 -2A>G splice acceptor site mutation by either precise insertion of this mutation or a frameshift modification (Supplemental Figure 14). Similar frameshift modifications were previously shown to recapitulate the loss of *DNAJC6* expression in hESC-derived neuronal cells[46], consistent with observations in *DNAJC6* variant carriers[45].

***SYNJ1* (PARK20)**

SYNJ1 encodes Synaptojanin-1 and was first linked to early onset autosomal recessive PD in 2013[49,50]. Synaptojanin-1 is predominantly expressed in neurons and is concentrated in presynaptic terminals. Homozygous *SYNJ1* mutations are linked to alterations in lipid metabolism and vesicle trafficking[49,50], as well as defects in autophagosome maturation[51]. We used a CRISPR/Cas9 approach to insert the R258Q substitution, which was identified in a homozygous state in several independent families with PD[49,50,52] (Supplemental Figure 15A). The iSCORE-PD collection includes clones that are homozygous for the R258Q mutation in *SYNJ1* or compound heterozygous for the R258Q and a frameshift allele at the same location. Both genotypes are expected to recapitulate the modification in *SYNJ1* associated with

disease (Supplemental Figure 15B,C). We performed Southern blot based zygosity analysis to exclude LOH in the homozygous edited cell line (Supplemental Figure 15D-F).

***VPS13C* (PARK23)**

Mutations in *VPS13C* were initially identified as the cause of autosomal recessive early-onset PD in 2016[53]. The initial functional analysis revealed that disruption of intermembrane lipid transfer protein vacuolar protein sorting 13 homolog C (VPS13C) causes decreased mitochondrial membrane potential, mitochondrial fragmentation, increased respiration rates, exacerbated PINK1/Parkin-dependent mitophagy, and transcriptional upregulation of *PRKN* in response to mitochondrial damage[53]. As the VPS13C protein is critical for the transport of lipids between the ER and endosome/lysosome, as well as for lipid droplet formation[54,55], loss of VPS13C causes the accumulation of lysosomes and altered lipid profiles[56]. We used CRISPR/Cas9-based editing to introduce the W395C and A444P variants into the *VPS13C* gene (Supplemental Figure 16A,E), both of which are found as homozygous or compound heterozygous mutations in PD patients[57,58]. While *VPS13C* is thought to cause PD through a loss of function mechanism, we included additional frameshift alleles in the iSCORE-PD collection to allow further investigation of the loss of function mechanism (Supplemental Figure 16B,C,F,G). We performed SNV-PCR followed by Sanger sequencing based zygosity analysis to exclude LOH in the homozygous *VPS13C* A444P lines (Supplemental Figure 16D).

GBA1

The *GBA1* gene codes for the enzyme Lysosomal acid glucosylceramidase, which is essential for maintaining glycosphingolipid homeostasis. While homozygous or compound heterozygous pathogenic variants in *GBA1*, associated with reduced glucosylceramidase activity, cause autosomal recessive Gaucher disease, heterozygous carriers of pathogenic *GBA1* variants have an elevated risk of developing PD[59,60]. *GBA1* mutations are currently considered the strongest risk factor for PD and Lewy body dementia with odd ratios between 1.4 to >10[61]. Given that *GBA1* mutations are present in approximately 3-20% of sporadic PD patients across different populations, *GBA1* represents the most prevalent genetic risk factor for PD[61]. Over 300 mutations in *GBA1* with variable risks for developing PD have been reported[60]. Among them, the splice site IVS2+1 mutation, causing missplicing and loss of *GBA1* expression, represents one of the most pathogenic alleles for PD[59,60]. To insert the IVS2+1 in the *GBA1* gene, we devised a CRISPR/Cas9-based targeting strategy that allows specific targeting of the *GBA1* gene and not the nearby highly homologous *GBAP1* pseudogene (Supplemental Figure 17A). To identify correctly targeted clones, we used a genotyping strategy that can conclusively distinguish between the *GBA1* and *GBAP1* pseudogene based on small sequence variation (Supplemental Figure 17B-D). Using this approach, we generated 2 heterozygous and 2 homozygous edited cell lines carrying the IVS2+1 mutation in *GBA1*. In addition, to allow comparison between the IVS2+1 and a loss of function allele, we also included cell lines with a frameshift mutation at the same genomic location in the iSCORE-PD collection.

References.

1 Blauwendraat, C., Nalls, M. A. & Singleton, A. B. The genetic architecture of Parkinson's disease. *The Lancet Neurology* (2019). https://doi.org:10.1016/s1474-4422(19)30287-x
2 Lill, C. M. *et al.* Launching the movement disorders society genetic mutation database (MDSGene). *Mov Disord* **31**, 607-609 (2016). https://doi.org:10.1002/mds.26651
3 Devine, M. J., Gwinn, K., Singleton, A. & Hardy, J. Parkinson's disease and alpha-synuclein expression. *Mov Disord* **26**, 2160-2168 (2011). https://doi.org:10.1002/mds.23948
4 Henderson, M. X., Trojanowski, J. Q. & Lee, V. M. alpha-Synuclein pathology in Parkinson's disease and related alpha-synucleinopathies. *Neurosci Lett* **709**, 134316 (2019). https://doi.org:10.1016/j.neulet.2019.134316
5 Wong, Y. C. & Krainc, D. alpha-synuclein toxicity in neurodegeneration: mechanism and therapeutic strategies. *Nat Med* **23**, 1-13 (2017). https://doi.org:10.1038/nm.4269
6 Li, H. *et al.* Highly efficient generation of isogenic pluripotent stem cell models using prime editing. *Elife* **11** (2022). https://doi.org:10.7554/eLife.79208
7 Polymeropoulos, M. H. *et al.* Mutation in the alpha-synuclein gene identified in families with Parkinson's disease. *Science* **276**, 2045-2047 (1997). https://doi.org:10.1126/science.276.5321.2045
8 Krüger, R. *et al.* Ala30Pro mutation in the gene encoding α-synuclein in Parkinson's disease. *Nature Genetics* **18**, 106-108 (1998). https://doi.org:10.1038/ng0298-106
9 Klein, C., Lohmann-Hedrich, K., Rogaeva, E., Schlossmacher, M. G. & Lang, A. E. Deciphering the role of heterozygous mutations in genes associated with parkinsonism. *Lancet Neurol* **6**, 652-662 (2007). https://doi.org:10.1016/S1474-4422(07)70174-6
10 Kitada, T. *et al.* Mutations in the parkin gene cause autosomal recessive juvenile parkinsonism. *Nature* **392**, 605-608 (1998). https://doi.org:10.1038/33416
11 Kasten, M. *et al.* Genotype-Phenotype Relations for the Parkinson's Disease Genes Parkin, PINK1, DJ1: MDSGene Systematic Review. *Mov Disord* **33**, 730-741 (2018). https://doi.org:10.1002/mds.27352
12 Kamienieva, I., Duszynski, J. & Szczepanowska, J. Multitasking guardian of mitochondrial quality: Parkin function and Parkinson's disease. *Transl Neurodegener* **10**, 5 (2021). https://doi.org:10.1186/s40035-020-00229-8
13 Pickrell, A. M. & Youle, R. J. The roles of PINK1, parkin, and mitochondrial fidelity in Parkinson's disease. *Neuron* **85**, 257-273 (2015). https://doi.org:10.1016/j.neuron.2014.12.007
14 Lucking, C. B. *et al.* Homozygous deletions in parkin gene in European and North African families with autosomal recessive juvenile parkinsonism. The European Consortium on Genetic Susceptibility in Parkinson's Disease and the French Parkinson's Disease Genetics Study Group. *Lancet* **352**, 1355-1356 (1998). https://doi.org:10.1016/s0140-6736(05)60746-5
15 Zhu, W. *et al.* Heterozygous PRKN mutations are common but do not increase the risk of Parkinson's disease. *Brain* **145**, 2077-2091 (2022). https://doi.org:10.1093/brain/awab456
16 Valente, E. M. *et al.* Hereditary Early-Onset Parkinson's Disease Caused by Mutations in <i>PINK1</i>. *Science* **304**, 1158-1160 (2004). https://doi.org:doi:10.1126/science.1096284
17 Goncalves, F. B. & Morais, V. A. PINK1: A Bridge between Mitochondria and Parkinson's Disease. *Life (Basel)* **11** (2021). https://doi.org:10.3390/life11050371
18 Ishihara-Paul, L. *et al.* <i>PINK1</i> mutations and parkinsonism. *Neurology* **71**, 896-902 (2008). https://doi.org:doi:10.1212/01.wnl.0000323812.40708.1f
19 van Duijn, C. M. *et al.* Park7, a novel locus for autosomal recessive early-onset parkinsonism, on chromosome 1p36. *Am J Hum Genet* **69**, 629-634 (2001). https://doi.org:10.1086/322996
20 Macedo, M. G. *et al.* The DJ-1L166P mutant protein associated with early onset Parkinson's disease is unstable and forms higher-order protein complexes. *Hum Mol Genet* **12**, 2807-2816 (2003). https://doi.org:10.1093/hmg/ddg304

21 Biosa, A. *et al.* Recent findings on the physiological function of DJ-1: Beyond Parkinson's disease. *Neurobiol Dis* **108**, 65-72 (2017). https://doi.org:10.1016/j.nbd.2017.08.005
22 Niki, T. *et al.* DJ-1-binding compound B enhances Nrf2 activity through the PI3-kinase-Akt pathway by DJ-1-dependent inactivation of PTEN. *Brain Res* **1729**, 146641 (2020). https://doi.org:10.1016/j.brainres.2019.146641
23 Darvish, H. *et al.* Detection of copy number changes in genes associated with Parkinson's disease in Iranian patients. *Neurosci Lett* **551**, 75-78 (2013). https://doi.org:10.1016/j.neulet.2013.07.013
24 Bonifati, V. *et al.* Mutations in the <i>DJ-1</i> Gene Associated with Autosomal Recessive Early-Onset Parkinsonism. *Science* **299**, 256-259 (2003). https://doi.org:doi:10.1126/science.1077209
25 Hu, J. *et al.* Gene-based burden analysis of damaging private variants in PRKN, PARK7 and PINK1 in Parkinson's disease cohorts of European descent. *Neurobiol Aging* **119**, 136-138 (2022). https://doi.org:10.1016/j.neurobiolaging.2022.07.012
26 Zimprich, A. *et al.* Mutations in LRRK2 cause autosomal-dominant parkinsonism with pleomorphic pathology. *Neuron* **44**, 601-607 (2004). https://doi.org:10.1016/j.neuron.2004.11.005
27 Kalogeropulou, A. F. *et al.* Impact of 100 LRRK2 variants linked to Parkinson's disease on kinase activity and microtubule binding. *Biochem J* **479**, 1759-1783 (2022). https://doi.org:10.1042/BCJ20220161
28 Gilks, W. P. *et al.* A common LRRK2 mutation in idiopathic Parkinson's disease. *The Lancet* **365**, 415-416 (2005). https://doi.org:10.1016/s0140-6736(05)17830-1
29 Healy, D. G. *et al.* Phenotype, genotype, and worldwide genetic penetrance of LRRK2-associated Parkinson's disease: a case-control study. *The Lancet Neurology* **7**, 583-590 (2008). https://doi.org:10.1016/s1474-4422(08)70117-0
30 Bonet-Ponce, L. & Cookson, M. R. LRRK2 recruitment, activity, and function in organelles. *FEBS J* **289**, 6871-6890 (2022). https://doi.org:10.1111/febs.16099
31 Tolosa, E., Vila, M., Klein, C. & Rascol, O. LRRK2 in Parkinson disease: challenges of clinical trials. *Nat Rev Neurol* **16**, 97-107 (2020). https://doi.org:10.1038/s41582-019-0301-2
32 Ramirez, A. *et al.* Hereditary parkinsonism with dementia is caused by mutations in ATP13A2, encoding a lysosomal type 5 P-type ATPase. *Nat Genet* **38**, 1184-1191 (2006). https://doi.org:10.1038/ng1884
33 Williams, D. R., Hadeed, A., al-Din, A. S., Wreikat, A. L. & Lees, A. J. Kufor Rakeb disease: autosomal recessive, levodopa-responsive parkinsonism with pyramidal degeneration, supranuclear gaze palsy, and dementia. *Mov Disord* **20**, 1264-1271 (2005). https://doi.org:10.1002/mds.20511
34 Fujii, T. *et al.* Parkinson's disease-associated ATP13A2/PARK9 functions as a lysosomal H(+),K(+)-ATPase. *Nat Commun* **14**, 2174 (2023). https://doi.org:10.1038/s41467-023-37815-z
35 Hatori, Y., Kanda, Y., Nonaka, S., Nakanishi, H. & Kitazawa, T. ATP13A2 modifies mitochondrial localization of overexpressed TOM20 to autolysosomal pathway. *PLoS One* **17**, e0276823 (2022). https://doi.org:10.1371/journal.pone.0276823
36 Dhanushkodi, N. R., Abul Khair, S. B., Ardah, M. T. & Haque, M. E. ATP13A2 Gene Silencing in Drosophila Affects Autophagic Degradation of A53T Mutant alpha-Synuclein. *Int J Mol Sci* **24** (2023). https://doi.org:10.3390/ijms24021775
37 Murphy, K. E., Cottle, L., Gysbers, A. M., Cooper, A. A. & Halliday, G. M. ATP13A2 (PARK9) protein levels are reduced in brain tissue of cases with Lewy bodies. *Acta Neuropathologica Communications* **1**, 11 (2013). https://doi.org:10.1186/2051-5960-1-11
38 Paisan-Ruiz, C. *et al.* Early-onset L-dopa-responsive parkinsonism with pyramidal signs due to ATP13A2, PLA2G6, FBXO7 and spatacsin mutations. *Mov Disord* **25**, 1791-1800 (2010). https://doi.org:10.1002/mds.23221

39 Shojaee, S. *et al.* Genome-wide linkage analysis of a Parkinsonian-pyramidal syndrome pedigree by 500 K SNP arrays. *Am J Hum Genet* **82**, 1375-1384 (2008). https://doi.org:10.1016/j.ajhg.2008.05.005

40 Conedera, S. *et al.* FBXO7 mutations in Parkinson's disease and multiple system atrophy. *Neurobiol Aging* **40**, 192 e191-192 e195 (2016). https://doi.org:10.1016/j.neurobiolaging.2016.01.003

41 Zhao, T. *et al.* Loss of nuclear activity of the FBXO7 protein in patients with parkinsonian-pyramidal syndrome (PARK15). *PLoS One* **6**, e16983 (2011). https://doi.org:10.1371/journal.pone.0016983

42 Zhou, Z. D. *et al.* F-box protein 7 mutations promote protein aggregation in mitochondria and inhibit mitophagy. *Hum Mol Genet* **24**, 6314-6330 (2015). https://doi.org:10.1093/hmg/ddv340

43 Burchell, V. S. *et al.* The Parkinson's disease-linked proteins Fbxo7 and Parkin interact to mediate mitophagy. *Nat Neurosci* **16**, 1257-1265 (2013). https://doi.org:10.1038/nn.3489

44 Kraus, F. *et al.* PARK15/FBXO7 is dispensable for PINK1/Parkin mitophagy in iNeurons and HeLa cell systems. *EMBO Rep* **24**, e56399 (2023). https://doi.org:10.15252/embr.202256399

45 Edvardson, S. *et al.* A deleterious mutation in DNAJC6 encoding the neuronal-specific clathrin-uncoating co-chaperone auxilin, is associated with juvenile parkinsonism. *PLoS One* **7**, e36458 (2012). https://doi.org:10.1371/journal.pone.0036458

46 Wulansari, N. *et al.* Neurodevelopmental defects and neurodegenerative phenotypes in human brain organoids carrying Parkinson’s disease-linked <i>DNAJC6</i> mutations. *Science Advances* **7**, eabb1540 (2021). https://doi.org:doi:10.1126/sciadv.abb1540

47 Roosen, D. A., Blauwendraat, C., Cookson, M. R. & Lewis, P. A. DNAJC proteins and pathways to parkinsonism. *FEBS J* **286**, 3080-3094 (2019). https://doi.org:10.1111/febs.14936

48 Jacquemyn, J. *et al.* Parkinsonism mutations in DNAJC6 cause lipid defects and neurodegeneration that are rescued by Synj1. *NPJ Parkinsons Dis* **9**, 19 (2023). https://doi.org:10.1038/s41531-023-00459-3

49 Quadri, M. *et al.* Mutation in the SYNJ1 gene associated with autosomal recessive, early-onset Parkinsonism. *Hum Mutat* **34**, 1208-1215 (2013). https://doi.org:10.1002/humu.22373

50 Krebs, C. E. *et al.* The Sac1 domain of SYNJ1 identified mutated in a family with early-onset progressive Parkinsonism with generalized seizures. *Hum Mutat* **34**, 1200-1207 (2013). https://doi.org:10.1002/humu.22372

51 Vanhauwaert, R. *et al.* The SAC1 domain in synaptojanin is required for autophagosome maturation at presynaptic terminals. *EMBO J* **36**, 1392-1411 (2017). https://doi.org:10.15252/embj.201695773

52 Olgiati, S. *et al.* PARK20 caused by SYNJ1 homozygous Arg258Gln mutation in a new Italian family. *Neurogenetics* **15**, 183-188 (2014). https://doi.org:10.1007/s10048-014-0406-0

53 Lesage, S. *et al.* Loss of VPS13C Function in Autosomal-Recessive Parkinsonism Causes Mitochondrial Dysfunction and Increases PINK1/Parkin-Dependent Mitophagy. *Am J Hum Genet* **98**, 500-513 (2016). https://doi.org:10.1016/j.ajhg.2016.01.014

54 Kumar, N. *et al.* VPS13A and VPS13C are lipid transport proteins differentially localized at ER contact sites. *J Cell Biol* **217**, 3625-3639 (2018). https://doi.org:10.1083/jcb.201807019

55 Cai, S. *et al.* In situ architecture of the lipid transport protein VPS13C at ER-lysosome membrane contacts. *Proc Natl Acad Sci U S A* **119**, e2203769119 (2022). https://doi.org:10.1073/pnas.2203769119

56 Hancock-Cerutti, W. *et al.* ER-lysosome lipid transfer protein VPS13C/PARK23 prevents aberrant mtDNA-dependent STING signaling. *J Cell Biol* **221** (2022). https://doi.org:10.1083/jcb.202106046

57 Smolders, S. *et al.* Contribution of rare homozygous and compound heterozygous VPS13C missense mutations to dementia with Lewy bodies and Parkinson's disease. *Acta Neuropathol Commun* **9**, 25 (2021). https://doi.org:10.1186/s40478-021-01121-w

58 Li, W., Fu, Y., Halliday, G. M. & Sue, C. M. PARK Genes Link Mitochondrial Dysfunction and Alpha-Synuclein Pathology in Sporadic Parkinson's Disease. *Front Cell Dev Biol* **9**, 612476 (2021). https://doi.org:10.3389/fcell.2021.612476

59 Aharon-Peretz, J., Rosenbaum, H. & Gershoni-Baruch, R. Mutations in the Glucocerebrosidase Gene and Parkinson's Disease in Ashkenazi Jews. *New England Journal of Medicine* **351**, 1972-1977 (2004). https://doi.org:10.1056/NEJMoa033277

60 Gan-Or, Z. *et al.* Differential effects of severe vs mild <i>GBA</i> mutations on Parkinson disease. *Neurology* **84**, 880-887 (2015). https://doi.org:doi:10.1212/WNL.0000000000001315

61 Blauwendraat, C. *et al.* Genetic modifiers of risk and age at onset in GBA associated Parkinson's disease and Lewy body dementia. *Brain* **143**, 234-248 (2020). https://doi.org:10.1093/brain/awz350
