# Supplementary material for: iSCORE-PD: an isogenic stem cell collection to research Parkinson’s disease
Source: Nat Commun. 2026 Jun 17;17:7682. doi: 10.1038/s41467-026-74355-8 (PMC13434110; doi:10.1038/s41467-026-74355-8)
Supplement: Supplementary file 4 [file 41467_2026_74355_MOESM2_ESM.pdf]

Description of Additional Supplementary Files

Supplementary Data 1. List of structural variants in parental WIBR3 hESC line.

Supplementary Data 2. List of coding variants in parental WIBR3 hESC line. The data table includes separate tabs for: (i) complete of ANNOVAR annotations for all variants; (ii) variants filtered for ClinVar pathogenicity; (iii) variants filtered for REVEL score ≥ 0.5; (iv) variants filtered for CADD-phred score ≥ 20; (v) variants filtered for ClinGen Dosage Sensitivity; and (vi) summary of pathogenic variants overlapping with genes from MSigDB related to Parkinson's, Alzheimer's and neurodegenerative diseases (KEGG_ALZHEIMERS_DISEASE, KEGG_PARKINSONS_DISEASE, HP_ALZHEIMER_DISEASE, HP_NEURODEGENERATION, or HP_PARKINSONISM).

Supplementary Data 3. Full description of all cell lines included in iSCORE-PD cell collection (control wild type, edited wild-types, *SNCA* A53T and A30P, *PRKN* EX3DEL, *PINK1* Q129X, *DJ1/PARK7* EX1-5DEL, *LRRK2* G2019S, *ATP13A2* FS, *FBXO7* R498X/FS, *DNAJC6* c.801-2 A>G/FS, *SYNJ1* R258Q/FS, *VPS13C* A444P/FS and W395C/FS and *GBA1* IVS2+1 G>A/FS). Details include Full cell line name, gene targeted, editing strategy, locus genetic description, rs-number, chromosome:basepair (hg38), allelic dosage, DNA annotation, predicted protein, Cellosaurus ID, editing pipeline (A/B).

Supplementary Data 4. Quality control calculation overview for iSCORE-PD cell line generation.

Supplementary Data 5. Complete description of unique and shared SNVs/Indels interactions between all analyzed cell lines. Red indicates on-target SNVs/Indels, green indicates SNVs/Indels shared within the editing group and black indicates SNVs/Indels detected between edited lines and EWTs.

Supplementary Data 6. Annotation of all SNVs/indels with an impact in coding DNA sequence (CDS). Intended gene edited alterations are highlighted in yellow, shared CDS mutation between correctly gene edited cell lines of the same editing group are highlighted in blue. The information for each editing group is separated in tabs.

Supplementary Data 7. Complete description of all SNVs/Indels in the protein coding region (splice sites, promoter region, introns, coding exons, 5' UTR and 3'UTR) or each cell line in the iSCORE-PD collection. Intended gene edited alterations are highlighted in yellow, shared CDS mutation between correctly gene edited cell lines of the same editing group are highlighted in blue. The information for each editing group is separated in tabs.

Supplementary Data 8. List of off-target hits detected by Cas-OFFinder. Tab1: shows final list post application of exclusion criteria. Tab2: contains a full list of hits. Information in this table: Editing group, implicated editing guide, chromosome affected, genomic location of off-target start, PAM sequence, modified sequence, number of mismatches, distance to PAM, genomic location of SNV, reference and alternative sequences, zygosity and affected cell line. Tab2 also includes Yes/No acceptance in the final list and reason for exclusion.

Supplementary Data 9. Collection of all oligonucleotide sequences used for genome engineering and QC steps. Primer sequences for Sanger and NGS genotyping, HDR oligonucleotide sequences, pegRNA sequences, sgRNA sequences, TALEN arm sequences, southern blot probe sequences and primers for SNV detection (zygosity evaluation).
